# The gut microbiome dysbiosis and regulation by fecal microbiota transplantation: umbrella review

**DOI:** 10.3389/fmicb.2023.1286429

**Published:** 2023-11-03

**Authors:** Xianzhuo Zhang, Xufei Luo, Liang Tian, Ping Yue, Mengyao Li, Kefeng Liu, Daoming Zhu, Chongfei Huang, Qianling Shi, Liping Yang, Zhili Xia, Jinyu Zhao, Zelong Ma, Jianlong Li, Joseph W. Leung, Yanyan Lin, Jinqiu Yuan, Wenbo Meng, Xun Li, Yaolong Chen

**Affiliations:** ^1^The First School of Clinical Medicine, Lanzhou University, Lanzhou, China; ^2^Evidence-Based Medicine Center, School of Basic Medical Sciences, Lanzhou University, Lanzhou, China; ^3^Department of General Surgery, The First Hospital of Lanzhou University, Lanzhou, China; ^4^The Second Clinical Medical College, Lanzhou University, Lanzhou, China; ^5^Department of Pharmacy, First Affiliated Hospital of Zhengzhou University, Zhengzhou, China; ^6^Department of Radiology, The Central Hospital of Enshi Tujia and Miao Autonomous Prefecture, Enshi, China; ^7^Division of Gastroenterology and Hepatology, UC Davis Medical Center and Sacramento VA Medical Center, Sacramento, CA, United States; ^8^Clinical Research Center, Big Data Center, The Seventh Affiliated Hospital, Sun Yat-sen University, Shenzhen, China; ^9^Research Unit of Evidence-Based Evaluation and Guidelines, Chinese Academy of Medical Sciences, School of Basic Medical Sciences, Lanzhou University, Lanzhou, China; ^10^Institute of Health Data Science, Lanzhou University, Lanzhou, China; ^11^WHO Collaborating Centre for Guideline Implementation and Knowledge Translation, Lanzhou, China

**Keywords:** gut microbiome, dysbiosis, fecal microbiota transplantation, meta-analyses, umbrella review

## Abstract

**Background:**

Gut microbiome dysbiosis has been implicated in various gastrointestinal and extra-gastrointestinal diseases, but evidence on the efficacy and safety of fecal microbiota transplantation (FMT) for therapeutic indications remains unclear.

**Methods:**

The gutMDisorder database was used to summarize the associations between gut microbiome dysbiosis and diseases. We performed an umbrella review of published meta-analyses to determine the evidence synthesis on the efficacy and safety of FMT in treating various diseases. Our study was registered in PROSPERO (CRD42022301226).

**Results:**

Gut microbiome dysbiosis was associated with 117 gastrointestinal and extra-gastrointestinal. Colorectal cancer was associated with 92 dysbiosis. Dysbiosis involving *Firmicutes* (phylum) was associated with 34 diseases. We identified 62 published meta-analyses of FMT. FMT was found to be effective for 13 diseases, with a 95.56% cure rate (95% CI: 93.88–97.05%) for recurrent *Chloridoids difficile* infection (rCDI). Evidence was high quality for rCDI and moderate to high quality for ulcerative colitis and Crohn’s disease but low to very low quality for other diseases.

**Conclusion:**

Gut microbiome dysbiosis may be implicated in numerous diseases. Substantial evidence suggests FMT improves clinical outcomes for certain indications, but evidence quality varies greatly depending on the specific indication, route of administration, frequency of instillation, fecal preparation, and donor type. This variability should inform clinical, policy, and implementation decisions regarding FMT.

## Introduction

Gut microbes, including commensal and pathogenic microbes, colonize in the gastrointestinal (GI) tract and significantly affect gut homeostasis and host health by producing various metabolites that influence the gut barrier and immunity (Guarner and Malagelada, 2003). Pathogenic or commensal microbes are frequently associated with disease development and progression (Guarner and Malagelada, 2003). Under normal conditions, gut microbes co-exist symbiotically with the host, maintain a dynamic balance, and play major roles in metabolism, nutrition, protection, immune regulation, hematopoiesis, anti-inflammation and anti-tumor functions (Guarner and Malagelada, 2003). However, when this balance is disrupted, normal bodily functions become impaired and GI and extra-GI diseases can emerge, including recurrent *Clostridioides difficile* infection (rCDI), inflammatory bowel disease (IBD), Crohn’s disease (CD), obesity and metabolic syndrome, among others (Borody and Khoruts, 2011; Allegretti et al., 2019; Lloyd-Price et al., 2019). Several databases catalog associations between the gut microbiome and disease but data analysis and visualization remain limited (Janssens et al., 2018; Cheng et al., 2020). Elucidating complex links between the gut microbiome and disease could enable novel therapies.

Fecal microbiota transplantation (FMT), which restores microbiome diversity and function by transferring screened feces from healthy donors to patient GI tracts, is gaining popularity for research and clinical use (Allegretti et al., 2019; Oka and Sartor, 2020; van der Lelie et al., 2021; Sorbara and Pamer, 2022). Numerous meta-analyses have examined FMT for specific diseases and subgroups (Allegretti et al., 2019; Green et al., 2020), but no study has quantitatively synthesized efficacy and safety across research or evaluated evidence quality and strength.

We aimed to systematically review relationships between gut microbiome dysbiosis and diseases and determine evidence on FMT efficacy and safety for various indications.

## Methods

### Database analysis

We used gutMDisorder (Jun 10, 2019, v1.0), a database cataloging relationships between diseases and the gut microbiome in humans, to examine associations (Cheng et al., 2020). The gutMDisorder contains 2,263 curated relationships between 579 gut microbes and 123 disorders as well as 77 interventions presented for humans. Each entry in the gutMDisorder database contains detailed information about an association, including the intestinal microbe, disorder name, intervention measures, and experimental technology. As the first manually curated resource for annotating gut microbiota functions, gutMDisorder enables the exploration of novel microbiota functions by leveraging previous computational methods and tools for predicting molecular functions (Cheng et al., 2020). Compared to other databases, gutMDisorder aggregates literature data from more sources and contains more detailed information, fulfilling the requirements of this study regarding data volume and information completeness. We analyzed and visualized these complex associations to identify key microbes linked to human diseases. For data collection, cluster analysis, and graphing code, we used Microsoft Excel (Microsoft Corporation, Redmond, WA, United States). In analyzing disease and dysbiosis in the database, we excluded post-intervention changes in microbes and only analyzed microbes directly related to the diseases. We generated graphs using ChiPlot, Origin 2021 (Origin Lab, Northampton) and Adobe Illustrator CS6 (Adobe, San Jose, CA, United States).

### Umbrella review

We searched ClinicalTrials.gov for ongoing FMT trials and conducted an umbrella review following Cochrane living review guidelines (Elliott et al., 2017; Hunt et al., 2018). An umbrella review provides an overview of evidence from systematic reviews and meta-analyses; a living review is continually updated (Aromataris et al., 2015; Neuenschwander et al., 2019; Zhang et al., 2020). As FMT develops, new evidence could emerge affecting findings, so we will update our review. We followed the Preferred Reporting Items for Systematic Reviews and Meta-Analysis (PRISMA) 2020 guideline (Page et al., 2021; Gates et al., 2022) and registered our protocol (CRD42022301226).

### Search strategy and selection criteria

We searched MEDLINE via PubMed, Embase, Cochrane Database of Systematic Reviews, Web of Science, China National Knowledge Infrastructure, Chinese Biomedical Literature Database and Wanfang Database for meta-analyses of randomized clinical trials (RCTs) or observational studies on FMT through October 2021, updating in June 2022 without language restrictions. We searched reference lists of identified studies (Appendix 1).

Studies were eligible if: (1) study design: meta-analyses of RCTs and/or observational studies; (2) participants: any condition or subtype of the disease; (3) intervention: FMT by any route of administration, donor type, infusion frequency or bacterial fluid status; and (4) outcomes: clinical remission, response or adverse events. We excluded: (1) systematic reviews without meta-analysis; (2) pre/probiotic research; and (3) animal research.

### Study selection

After eliminating duplicates, two reviewers independently screened the titles and abstracts and the full texts. Disagreements were resolved by consensus or appeal to a senior reviewer. We recorded reasons for exclusion.

### Data extraction

Two reviewers extracted data independently and a third checked accuracy. For each study, we extracted: (1) characteristics: author, publication year, country, disease, studies, participants, age, sex, follow-up, adverse events; and (2) FMT details: participants, outcome measures including relative risk (RR), odds ratio (OR), mean difference (MD), weighted mean difference (WMD) and standardized mean difference (SMD), and heterogeneity (*I*^2^, *P*).

### Quality assessment

Two reviewers independently assessed meta-analysis quality using A Measurement Tool to Assess Systematic Reviews (AMSTAR-2) (16 items; Shea et al., 2017). We used the Grading of Recommendations Assessment Development and Evaluation (GRADE) approach to assess evidence quality as high, moderate, low or very low (Guyatt et al., 2011).

### Data synthesis

We synthesized data from the most recent meta-analysis for each disease, estimating heterogeneity (*I*^2^ > 50%, *p* < 0.10). If no remission rate was reported, we used another outcome. We chose the study with the highest number of RCTs or cohort studies if two or more studies for the same disease category and outcome were published within the same 24-month period. If two or more studies published in the same period had the same number of RCTs or cohort studies, we included the study with the highest AMSTAR-2 score. When both cohort studies and case series were included in a meta-analysis, and subgroup analysis was stratified by study design, the cohort design sub-analysis was selected for inclusion in the summary forest plots. We summarized subgroup analyses by administration route, donor type, frequency or bacterial fluid status.

## Results

### Search results

**Figure 1:**
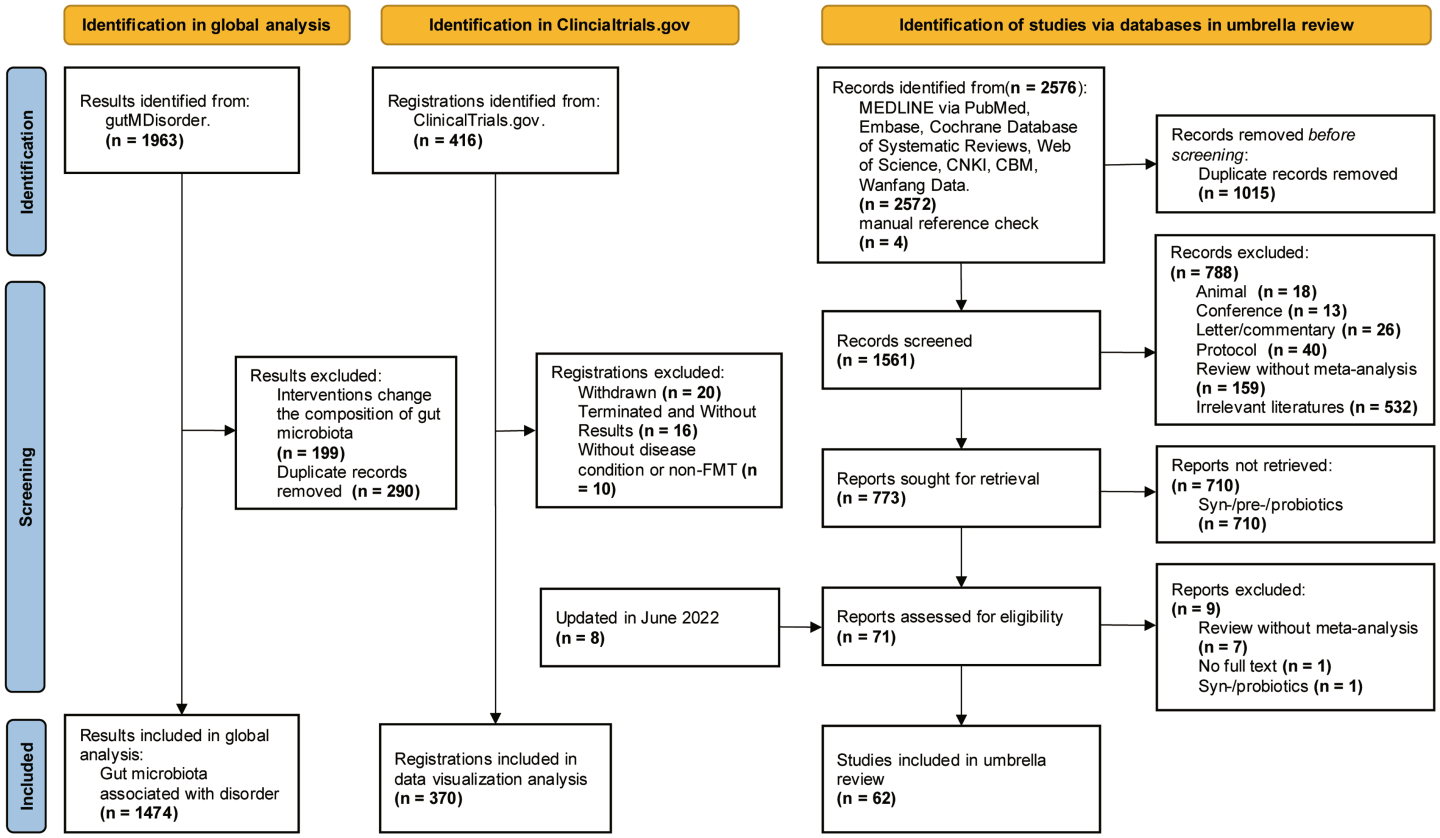
Flow diagram depicting results for inclusion.

We conducted a systematic search of gutMDisorder, trial registers and databases, identifying 1,474 microbe-disorder associations for analysis and 370 trial registrations for data visualization (Figure 1). Our umbrella review yielded 2,576 publications, of which 62 meta-analyses (Supplementary Table S1) met inclusion criteria.

### Gut microbiota associations with disorders

**Figure 2:**
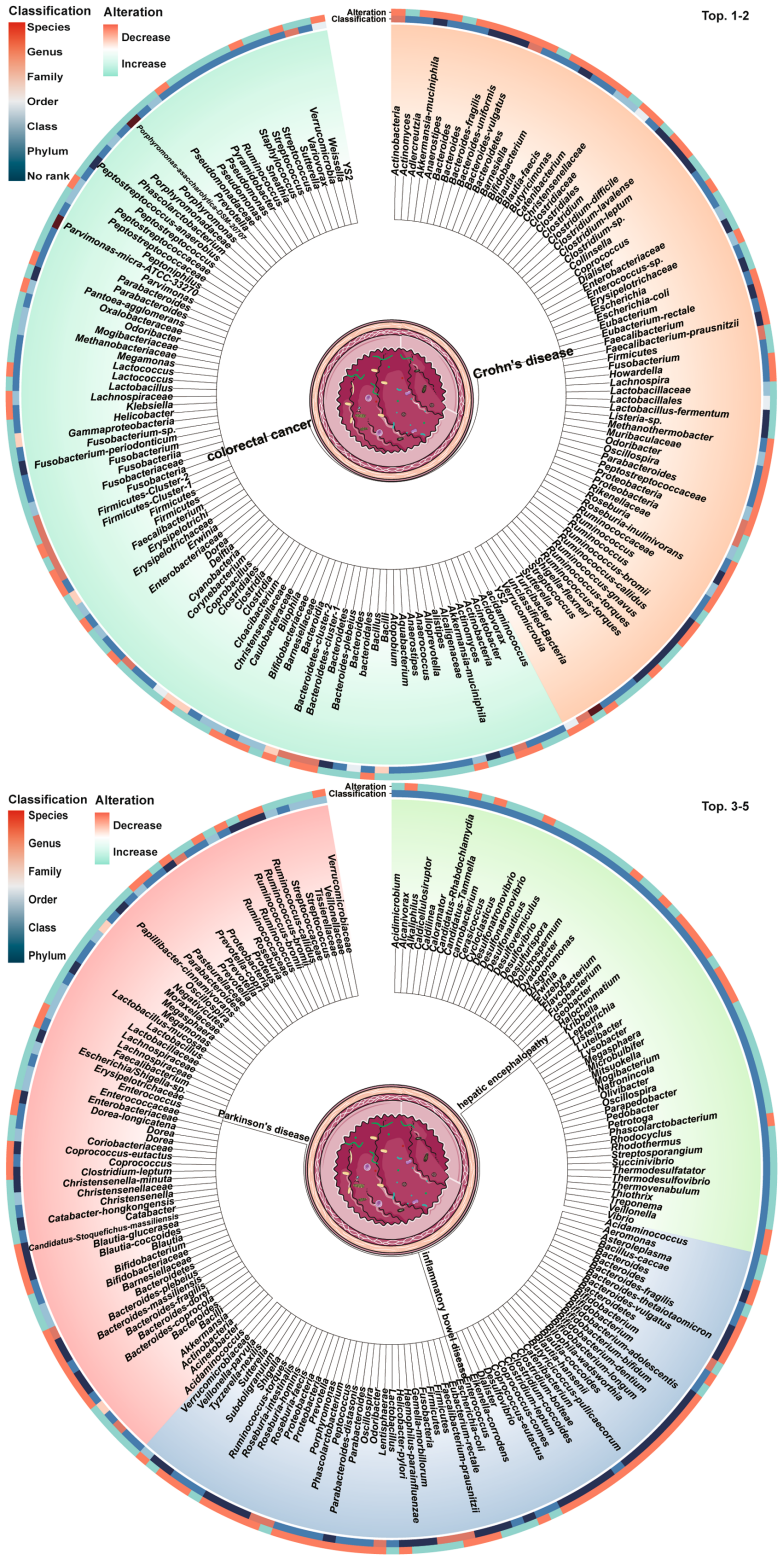
Top 5 diseases and their associated gut microbes dysbioses. The tree trunks represent the diseases while the leaves show the specific microbial dysbioses linked to each disease. The inner circle heatmap indicates the taxonomic classification of the microbes, and the outer circle heatmap reveals the over- and under-abundance of particular microbes for each disease cluster. Full radial dendrograms were in Supplementary Figure S1. Microbe names use hyphens in place of spaces.

**Figure 3:**
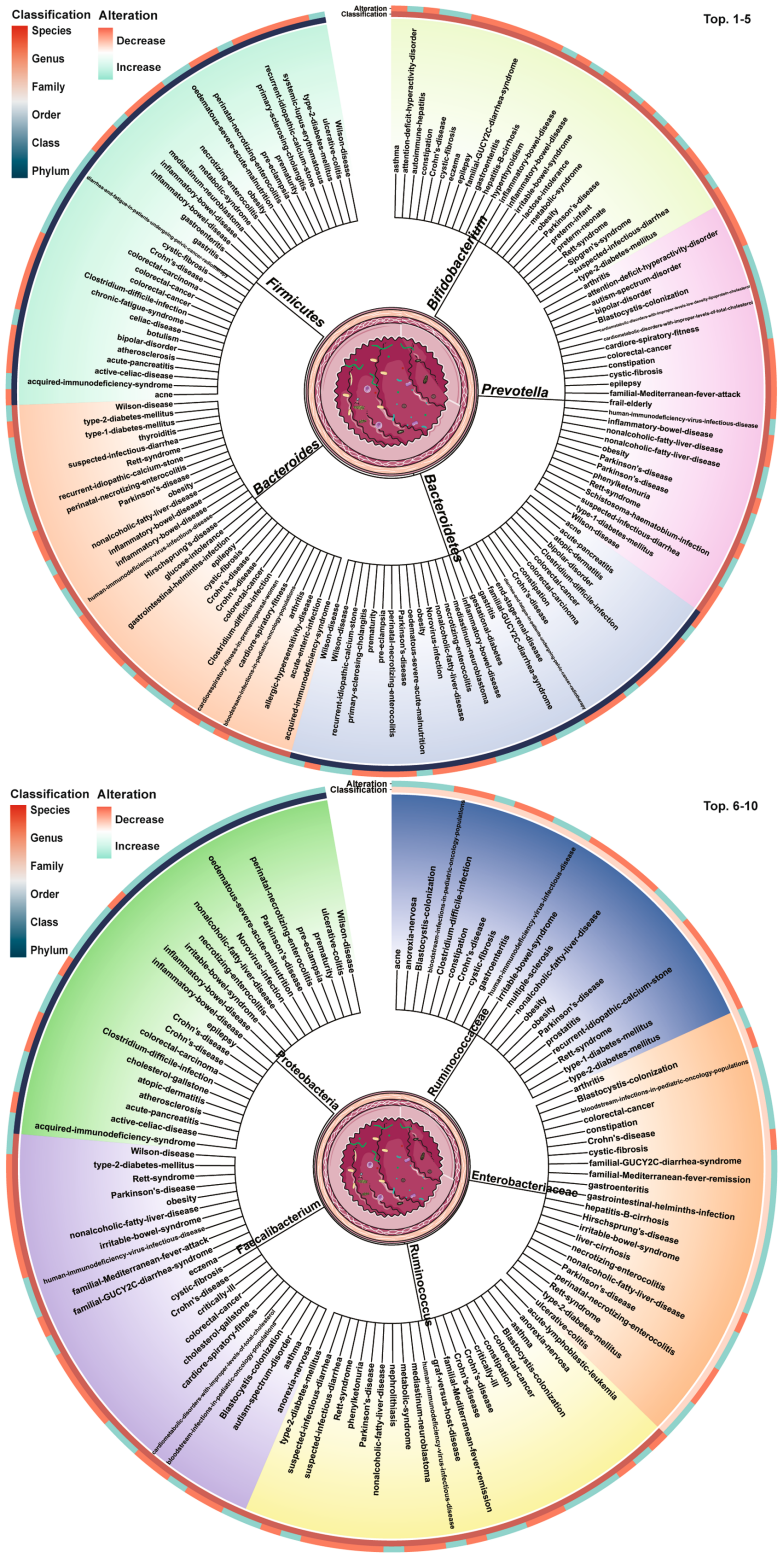
Top 10 gut microbes dysbioses and their associated diseases. The tree trunks denote the microbes and the leaves present the diseases related to dysbioses of each microbe. The inner circle heatmap indicates the taxonomic classification of the microbes, and the outer circle heatmap reveals the over- and under-abundance of particular microbes for each disease cluster. Full radial dendrograms were in Supplementary Figure S2. Microbe names use hyphens in place of spaces.

**Figure 4:**
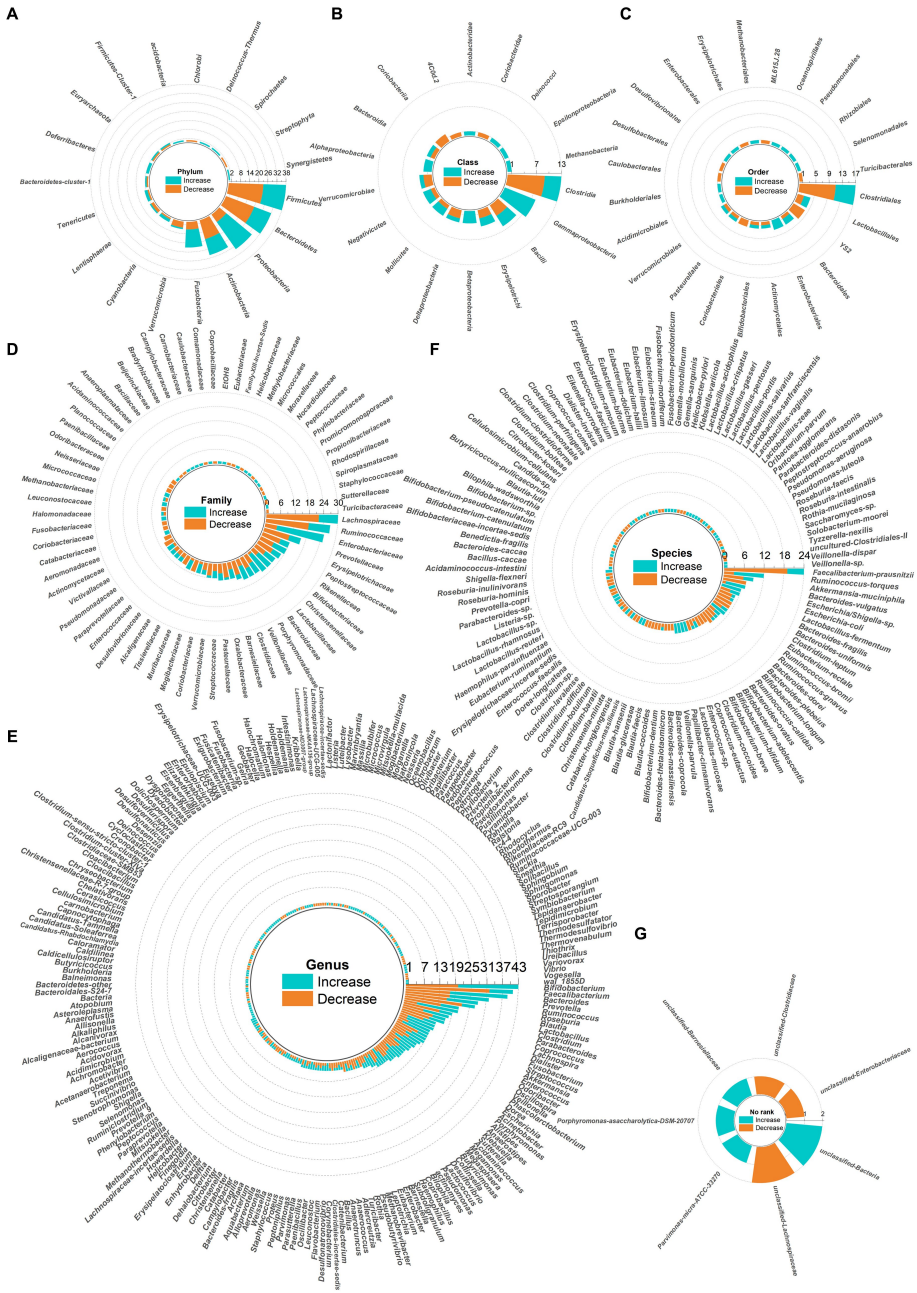
Radial bar charts showing relative abundances of key microbes grouped by taxonomic classification. Microbe names use hyphens in place of spaces. **(A)** Fhylum; **(B)** Class; **(C)** Order; **(D)** Family; **(E)** Genus; **(F)** Species; **(G)** No rank.

Dysbiosis of 479 gut microbes is associated with 117 gastrointestinal and extra-gastrointestinal diseases (Supplementary Figure S1). Figure 2 used a radial dendrogram to display the top 5 diseases associated with gut microbiome dysbiosis. Colorectal cancer associated with the most (92) dysbioses, followed by CD (71), Parkinson’s disease (66) and IBD (61). Patients with COVID-19 had decreased anti-inflammatory *Lachnospiraceae*, Roseburia, *Eubacterium* and *Faecalibacterium prausnitzii*, but increased opportunistic pathogens like *Clostridium hathewayi*, *Enterobacteriaceae*, *Enterococcus*, *Actinomyces viscosus* and *Bacteroides nordii* (Zuo et al., 2020). Figure 3 employed a similar radial dendrogram to present the top 10 gut microbes exhibiting dysbiosis and their associated diseases. *Firmicutes* (phylum) associated with the most (34) diseases, followed by *Bacteroides* (genus, 30), *Bacteroidetes* (phylum, 29) and *Prevotella* (genus, 27). Figure 4 used radial bar charts to display the prevalence of critical microbes across taxonomy. Segmenting by taxonomic classification allowed identification of influential microbe groups at different levels associated with diseases. Key dysregulated microbes spanned phylum to species: *Firmicutes* (34), *Clostridiales* (14), *Enterobacteriaceae* (22), *Bacteroides* (30), *Faecalibacterium prausnitzii* (13) and *unclassified Lachnospiraceae* (2).

### Registered clinical trials using fecal microbiota transplantation

**Figure 5:**
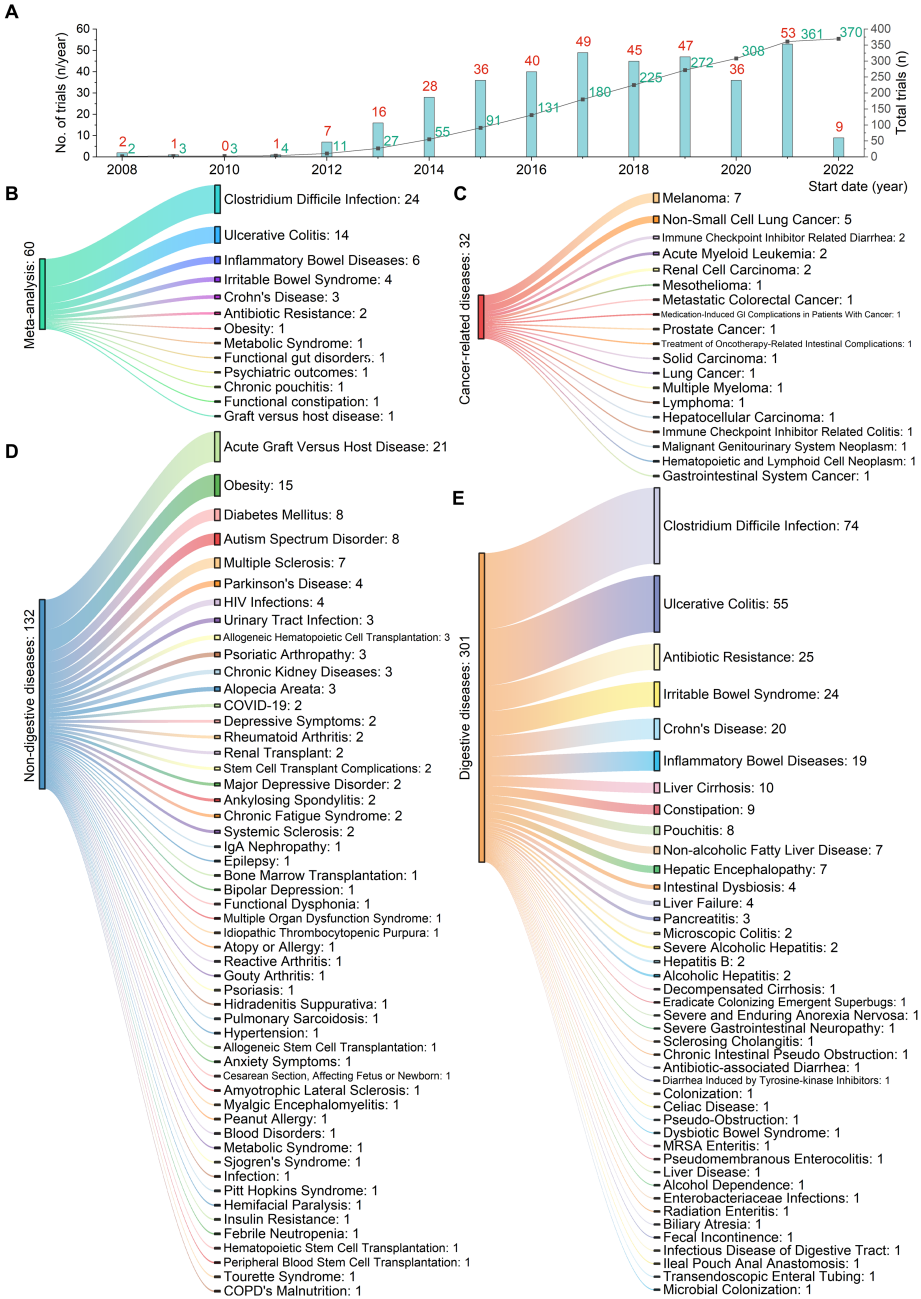
Registered clinical trials using fecal microbiota transplantation for 114 digestive or non-digestive diseases from 2008 to 2022. **(A)** Number of trials (red) and cumulative total (green) per year. **(B)** 13 conditions from 62 meta-analyses. **(C)** 19 cancer-related conditions. FMT has been applied not only to treat gastrointestinal cancers themselves, but also cancers of other organ systems and gastrointestinal side effects arising from other cancer treatments. **(D)** 53 non-digestive conditions. **(E)** 42 digestive conditions.

Analysis of 370 registered clinical trial entries presented an overview of FMT used for treating various digestive and non-digestive diseases since 2008 (Figure 5). Figure 5A showed the number of registered trials per year (red) and the cumulative total number (green). Figure 5C illustrated 19 cancer-related conditions, where FMT had been applied not only to treat gastrointestinal cancers themselves, but also cancers of other organ systems and gastrointestinal side effects arising from other cancer treatments. Figure 5D presented 53 non-digestive diseases. Figure 5E demonstrated 42 digestive diseases.

### Study characteristics and quality assessment

**Figure 6:**
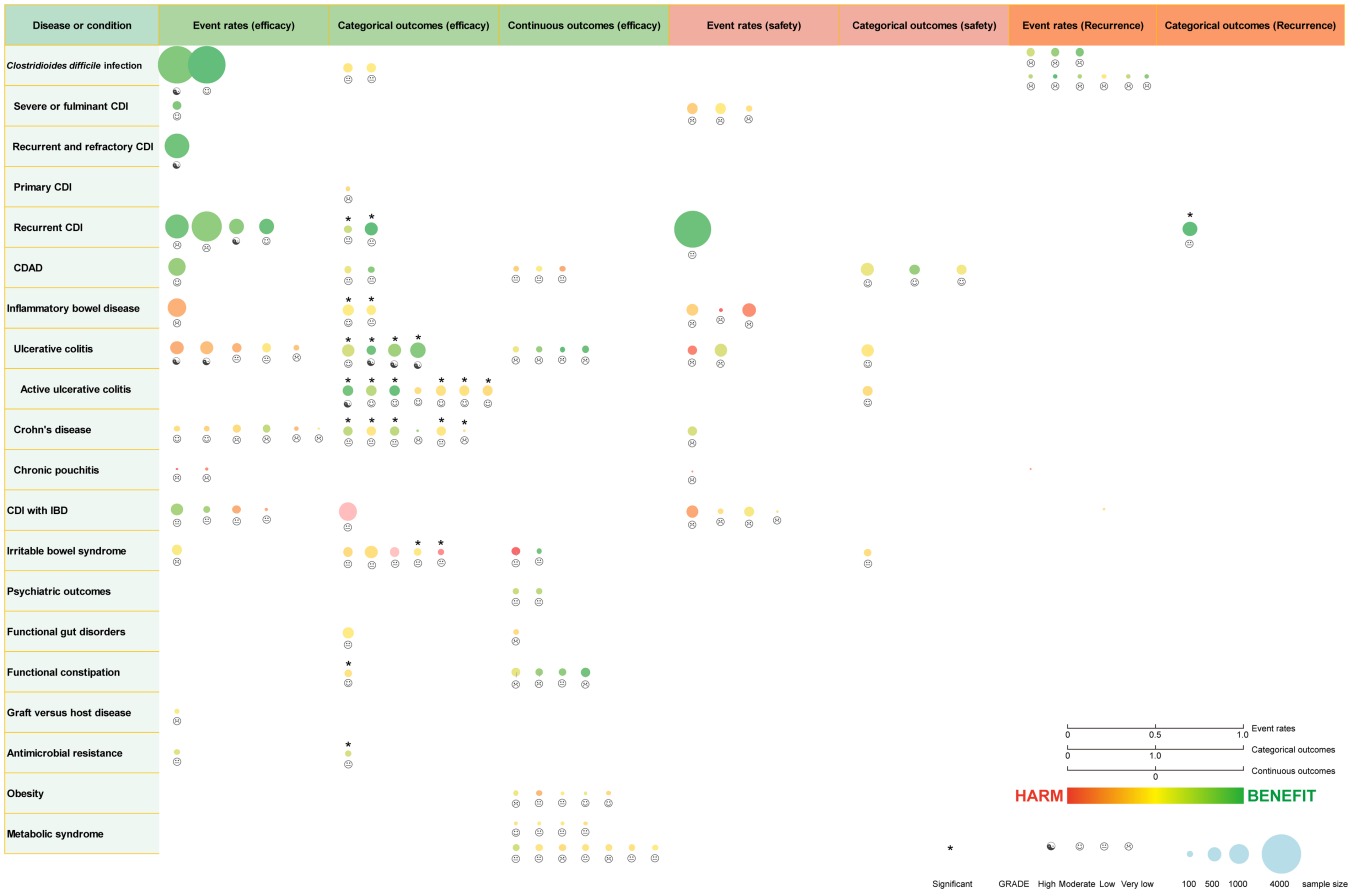
Evidence mapping of reported efficacy, safety and recurrence outcomes of fecal microbiota transplantation in treating various diseases. CDI, *Clostridioides difficile* infection; CDAD, *Clostridioides difficile*-associated diarrhea; IBD, inflammatory bowel disease. GRADE, Grading of Recommendations Assessment Development and Evaluation.

**Figure 7:**
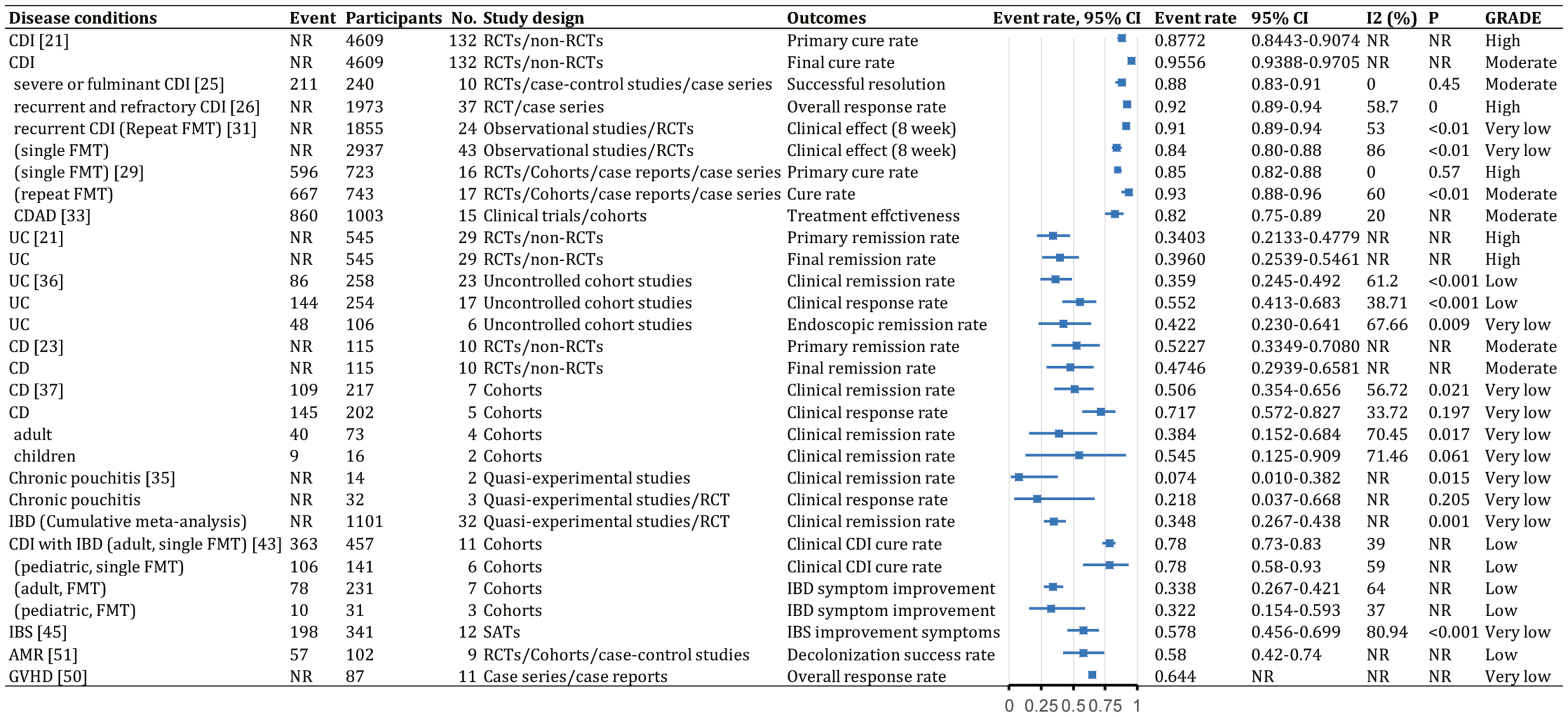
Event rates (efficacy) of fecal microbiota transplantation in the treatment of various diseases. 95% CI, 95% confidence interval; RCDI, recurrent *Clostridioides difficile* infection; IBD, inflammatory bowel disease; UC, ulcerative colitis; AUC, active UC; CD, Crohn’s disease; AMR, antimicrobial resistance; IBS, irritable bowel syndrome; CDAD, *Clostridioides difficile*-associated diarrhea; GVHD, graft versus host disease; RCTs, randomized clinical trials; SATs, single-arm trials; NR, not reported; NA, not applicable.

**Figure 8:**
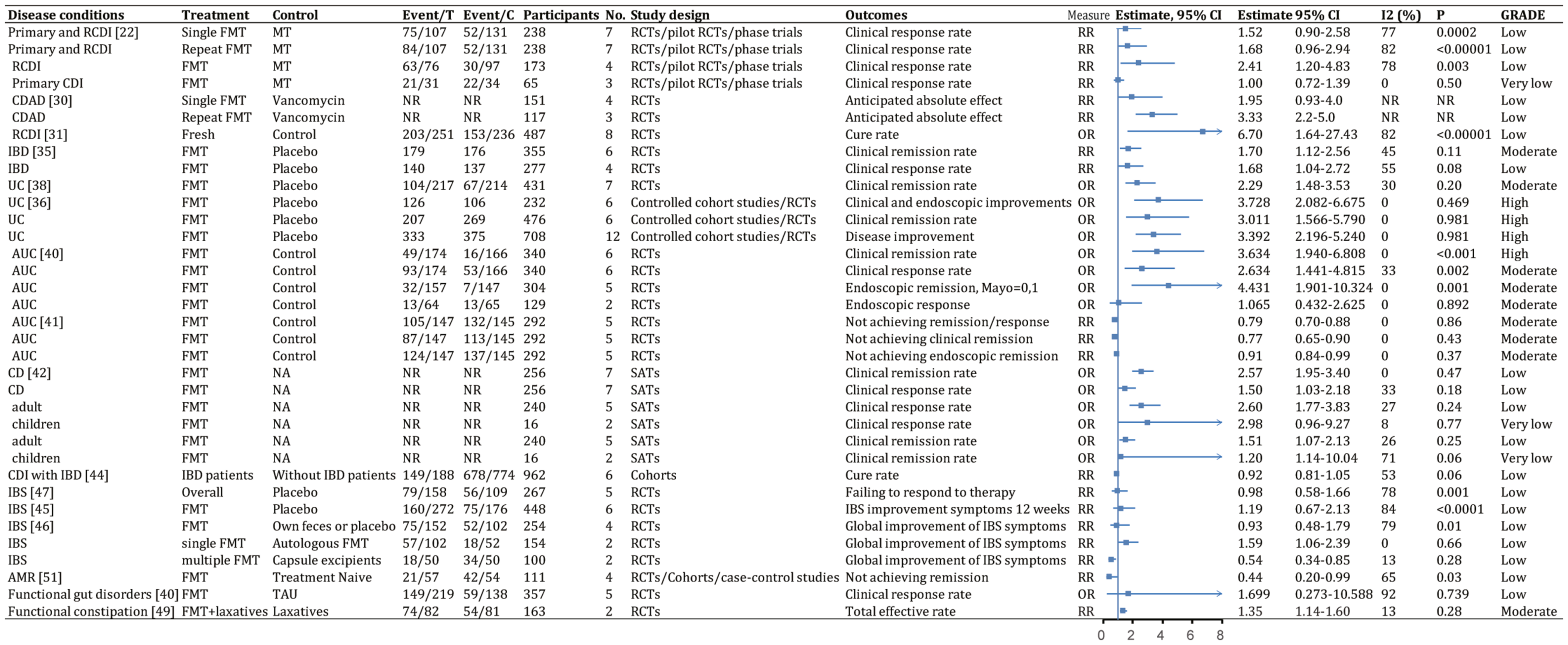
Categorical outcomes (efficacy) of fecal microbiota transplantation in the treatment of various diseases. 95% CI, 95% confidence interval; RCDI, recurrent *Clostridioides difficile* infection; IBD, inflammatory bowel disease; UC, ulcerative colitis; AUC, active UC; CD, Crohn’s disease; AMR, antimicrobial resistance; IBS, irritable bowel syndrome; CDAD, *Clostridioides difficile*-associated diarrhea; GVHD, graft versus host disease; RCTs, randomized clinical trials; SATs, single-arm trials; NR, not reported; NA, not applicable.

**Figure 9:**
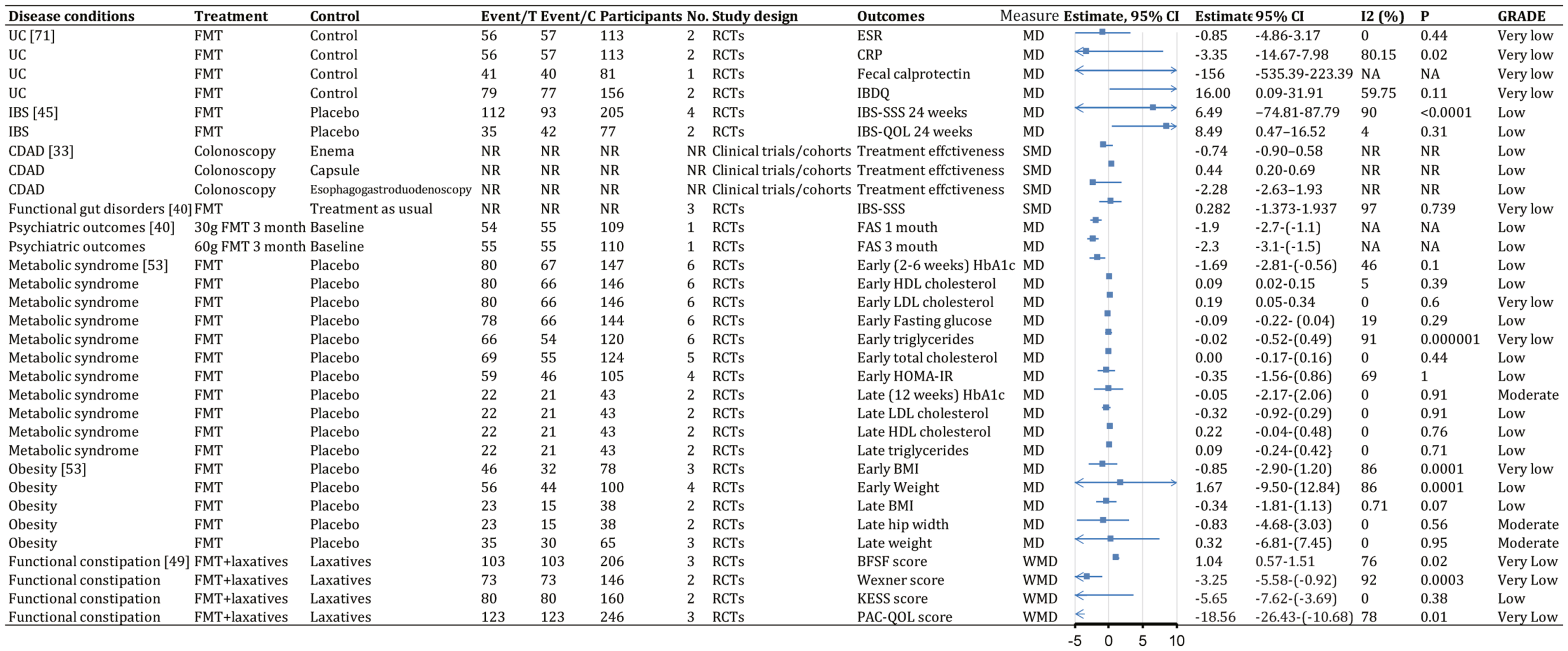
Continuous outcomes (efficacy) of fecal microbiota transplantation in the treatment of various diseases. 95% CI, 95% confidence interval; RCDI, recurrent *Clostridioides difficile* infection; IBD, inflammatory bowel disease; UC, ulcerative colitis; AUC, active UC; CD, Crohn’s disease; AMR, antimicrobial resistance; IBS, irritable bowel syndrome; CDAD, *Clostridioides difficile*-associated diarrhea; GVHD, graft versus host disease; RCTs, randomized clinical trials; SATs, single-arm trials; NR, not reported; NA, not applicable.

**Figure 10:**
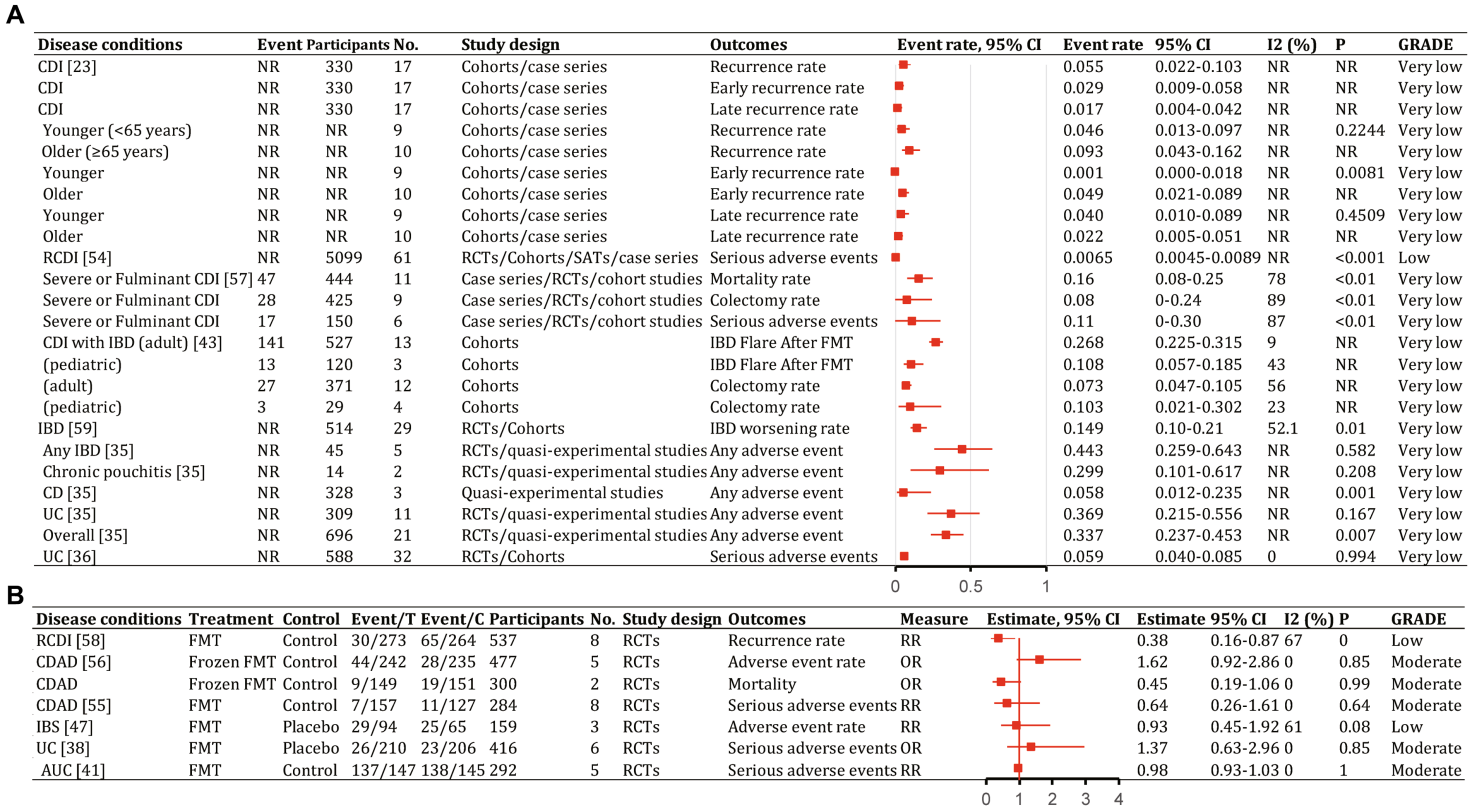
Recurrence and safety of fecal microbiota transplantation in the treatment of various diseases. 95% CI, 95% confidence interval; RCDI, recurrent *Clostridioides difficile* infection; IBD, inflammatory bowel disease; UC, ulcerative colitis; AUC, active UC; CD, Crohn’s disease; AMR, antimicrobial resistance; IBS, irritable bowel syndrome; CDAD, *Clostridioides difficile*-associated diarrhea; GVHD, graft versus host disease; RCTs, randomized clinical trials; SATs, single-arm trials; NR, not reported; NA, not applicable.

We identified 62 meta-analyses examining FMT for clinical outcomes (Supplementary Table S1 in details). Excluding duplicates left 13 conditions (Figure 5B): CDI (*n* = 24), UC (*n* = 14), IBD (*n* = 6), IBS (*n* = 4), CD (*n* = 3), AMR (*n* = 2), obesity (*n* = 1), metabolic syndrome (*n* = 1), functional gut disorders (*n* = 1), psychiatric outcomes (*n* = 1), chronic pouchitis (*n* = 1), functional constipation (*n* = 1) and GVHD (*n* = 1). We utilized an evidence mapping approach to visually present the reported efficacy, safety, and recurrence outcomes of FMT across 20 disease conditions after screening (Figure 6). In the map, the bubble size represents sample size, the emoji denotes evidence quality rating, and color ranging from red to yellow to green indicates the degree of FMT treatment efficacy on diseases, corresponding to the effect size values. Detailed outcome measures are shown in forest plots (Figures 7–10). Subgroup analyses (route of administration, frequency of instillation, fecal preparation, and donor type) and more information are presented in Supplementary Table S2.

The quality scores of included studies were presented in Supplementary Table S1. GRADE evidence ratings for each outcome were displayed in forest plots (Figures 7–10). Overall, the evidence was high quality for CDI and moderate to high quality for UC and CD, but low to very low quality for other diseases.

### Gastrointestinal diseases

#### *Clostridioides difficile* infection

FMT was consistently found to be one of the most effective treatments for CDI across the meta-analyses. A recent comprehensive meta-analysis of 132 studies involving 4,609 participants found that fecal microbiota transplantation (FMT) had an overall final cure rate of 95.56% (95% CI, 93.88–97.05%; moderate quality evidence) for rCDI (Figure 7; Lai et al., 2019). Compared with medical therapy alone, FMT had a greater treatment effect for rCDI (RR, 2.41; 95% CI, 1.20–4.83; low) than for primary CDI (RR, 1.00; 95% CI, 0.72–1.39; very low; Figure 8; Singh et al., 2021). With stratification by age, FMT cure rates were higher in younger patients (<65 years) compared with older patients (≥65 years) [99.4% (95% CI, 96.9–100%) vs. 87.0% (95% CI, 81.6–91.6%)] (Supplementary Table S2; Li et al., 2016). Repeated FMT (RR, 1.68; 95% CI, 0.96–2.94) showed higher response rate than a single FMT (RR, 1.52; 95% CI, 0.90–2.58; Figure 8; Singh et al., 2021). By delivery route, colonoscopy to the lower gastrointestinal tract achieved the highest cure rate (0.95; 95% CI, 0.92–0.97; Supplementary Table S2; Ramai et al., 2021). Studies from Asia and Europe reported that FMT tended to be more effective when delivered through the upper gastrointestinal tract (58.6 and 45.2%, respectively) rather than the lower gastrointestinal tract (20.7 and 40.5%, respectively), whereas North American studies preferred administering FMT through the lower gastrointestinal tract (41.0% vs. 23.9%; Lai et al., 2019).

FMT also seemed beneficial for CDI subtypes. For severe or fulminant CDI, a single FMT had an overall successful resolution rate of 0.88 (95% CI, 0.83–0.91; moderate; Figure 7; Song et al., 2022). For recurrent and refractory CDI, the overall response rate was 0.92 (95% CI, 0.89–0.94; high; Figure 7), with multiple infusions via the lower gastrointestinal tract achieving the best response (0.95; 95% CI, 0.92–0.97; Supplementary Table S2; Quraishi et al., 2017).

A network meta-analysis of several antibiotics found that FMT after vancomycin therapy was likely the most effective approach for clinical cure (surface under the cumulative ranking curve score, 83%) and preventing multiple rCDI recurrence (75%), while tolevamer also prevented recurrence (87%) (Dembrovszky et al., 2021). Another network meta-analysis showed FMT was optimal, especially compared with vancomycin or fidaxomicin (Rokkas et al., 2019). For rCDI, repeated FMT had higher cure rate than a single FMT (0.93; 95% CI, 0.88–0.96, high vs. 0.85; 95% CI, 0.82–0.88 moderate; Figure 7; Cold et al., 2021). Repeated FMT vs. vancomycin had a more significant effect (RR, 3.33; 95% CI, 2.2–5.0; Figure 8; Baunwall et al., 2020). This finding was supported by another meta-analysis (cure rate with single vs. repeat therapy OR, 0.09; 95% CI, 0.04–3.13; Supplementary Table S2; Yang et al., 2021).

Studies largely found consistent results for FMT delivery via colonoscopy to the lower vs. upper gastrointestinal tract for outcomes such as clinical remission (Ianiro et al., 2018; Tariq et al., 2019; Baunwall et al., 2020; Pomares Bascuñana et al., 2021). Fresh FMT vs. control significantly cured CDAD (OR, 6.70; 95% CI, 1.64–27.43; low-quality evidence; Figure 8; Yang et al., 2021). Two storage temperatures for frozen FMT, −20°C (0.889; 95% CI, 0.684–1.000) and − 70°C (0.901; 95% CI, 0.832–0.971), had similar cure rates (Supplementary Table S2; Cold et al., 2021). Bowel cleansing before FMT was more effective than without it (0.879; 95% CI, 0.812–0.947 vs. 0.822; 95% CI, 0.787–0.856). Cure rates after aerobic and anaerobic processing were 0.844 and 0.803, respectively (Supplementary Table S2; Cold et al., 2021).

### Inflammatory bowel disease (including ulcerative colitis, Crohn’s disease and chronic pouchitis)

There was evidence that FMT vs. placebo improved clinical outcomes in IBD (clinical remission: RR, 1.70; 95% CI, 1.12–2.56; moderate; Figure 8; Scaldaferri et al., 2018). A meta-analysis found 37% of patients achieved clinical remission, and 54% showed clinical response (Scaldaferri et al., 2018). Efficacy outcomes were better with frozen fecal material and universal donors. Furthermore, CD patients appeared to benefit more than UC and chronic pouchitis patients, based on primary remission rate (UC: 34.03% vs. CD: 52.27%), final remission rate (UC: 39.60% vs. CD: 47.46%), clinical remission rate (UC: 35.9% vs. CD: 50.6% vs. chronic pouchitis: 7.4%), and clinical response rate (UC: 55.2% vs. CD: 71.7% vs. chronic pouchitis: 21.8%; Supplementary Table S2; Scaldaferri et al., 2018; Lai et al., 2019; Zhao et al., 2020; Wu et al., 2021). Cumulative meta-analyses from 2013 to 2020 showed increasing clinical remission rates over time (0.348, 95% CI, 0.267–0.438) owing to improvements in FMT or novel FMT-based therapeutics (Supplementary Table S2; Scaldaferri et al., 2018).

### Ulcerative colitis

Studies of FMT for UC showed significant improvement vs. placebo (OR, 3.392; 95% CI, 2.196–5.240; high; Figure 8; Tang et al., 2020; Zhao et al., 2020). The final remission rate for UC with FMT was 39.6% (95% CI, 25.39–54.61%; high; Figure 7; Lai et al., 2019). Furthermore, FMT via the lower vs. upper gastrointestinal tract led to greater clinical remission (44.0% vs. 31.7%; Zhao et al., 2020). Higher clinical remission rate occurred with total stool dosage over 275 g vs. less than 275 g (51.9% vs. 29.5%; Zhao et al., 2020). There were no significant differences in clinical remission based on patient age, single vs. repeated infusions, fresh vs. frozen FMT, or antibiotic pretreatment (Paramsothy et al., 2017; Zhao et al., 2020).

There was strong evidence that FMT for active UC improved clinical remission (OR, 3.634; 95% CI, 1.940–6.808; high), clinical response (OR, 2.634; 95% CI, 1.441–4.815; moderate), and endoscopic remission (OR, 4.431; 95% CI, 1.901–10.324; moderate; Figure 8; Green et al., 2020). However, endoscopic response showed no significant improvement (OR, 1.065; 95% CI, 0.432–2.625; moderate) in a small subgroup analysis (Figure 8; Green et al., 2020; Liu et al., 2021). FMT vs. control was associated with a higher combined rate of clinical and endoscopic remission (RR, 0.79; 95% CI, 0.70–0.88; moderate; Figure 8; Liu et al., 2021). FMT via the lower gastrointestinal tract led to a greater combination of clinical remission and endoscopic response (RR, 0.79; 95% CI, 0.70–0.89; Supplementary Table S2; Liu et al., 2021). Clinical remission rates were slightly higher with pooled donor stool (2–7 donors) vs. placebo or autologous FMT (RR, 0.69; 95% CI, 0.56–0.85) and with multiple infusions vs. control (RR, 0.76; 95% CI, 0.62–0.93; Supplementary Table S2; Liu et al., 2021).

### Crohn’s disease

Summary estimates found a 47.46% (95% CI, 29.39–65.81%; moderate) final remission rate and 71.7% (95% CI, 57.2–82.7%; very low) clinical response rate after FMT for CD (Figure 7; Lai et al., 2019; Wu et al., 2021). FMT improved clinical response (OR, 2.57; 95% CI, 1.95–3.40; low) and clinical remission (OR 1.50; 95% CI, 1.03–2.18; low) in CD (Figure 8; Ye et al., 2020). Subgroup analysis found 54.5% (95% CI, 12.5–90.9%) of children and 38.4% (95% CI, 15.2–68.4%) of adults achieved clinical remission (Supplementary Table S2; Wu et al., 2021). The clinical remission rate was 38.7% (95% CI, 1.5–96.3%) for fecal infusion in the upper gastrointestinal tract vs. 32.2% (95% CI, 1.16–63.2%) for the lower gastrointestinal tract (Supplementary Table S2; Wu et al., 2021). Fresh stool infusion was associated with a better clinical response rate (50.0%; 95% CI, 21.8–78.2%) than frozen stool infusion (29.9%; 95% CI, 4.4–79.8%; Supplementary Table S2; Wu et al., 2021). The clinical response rate was higher without antibiotic pretreatment (56.4%; 95% CI, 4.37–80.5%) than with pretreatment (36.8%; 95% CI, 7.6–80.5%; Supplementary Table S2; Wu et al., 2021).

### Chronic pouchitis

Few studies have examined FMT for chronic pouchitis. A meta-analysis of two studies with 14 patients reported a clinical remission rate of 7.4% (95% CI, 1.0–38.2%; very low; Figure 7; Scaldaferri et al., 2018). Three studies evaluated 32 patients, with a clinical response rate of 21.8% (95% CI, 3.7–66.8%; very low; Figure 7; Scaldaferri et al., 2018).

#### *Clostridioides difficile* infection patients with inflammatory bowel disease

For patients with IBD and CDI, FMT was an effective therapy. The overall clinical cure rate for CDI in adults with IBD was 78% (95% CI, 73–83%; low) and for pediatric patients was 78% (95% CI, 58–93%; low) (Figure 7; Tariq et al., 2021). CDI cure rates did not differ significantly between patients with and without IBD (RR, 0.92; 95% CI, 0.81–1.05; low; Figure 8; Chen et al., 2018). Subgroup analysis found similar initial CDI cure rates for CD (0.78; 95% CI, 0.70–0.84; low) and UC (0.85; 95% CI, 0.77–0.91; low; Supplementary Table S2; Chen et al., 2018). For IBD symptom improvement, 78 of 231 adults (33.8%; 95% CI, 26.7–42.1%; low) and 10 of 31 children (32.2%; 95% CI, 15.4–59.3%; low) showed improvement (Figure 7; Tariq et al., 2021).

### Irritable bowel syndrome

Among patients with IBS, 57.8% (95% CI, 45.6–69.9%; very low) showed significant improvement (Figure 7; Xu et al., 2019; Elhusein and Fadlalmola, 2022). Fresh or frozen donor stool administered via colonoscopy or nasojejunal tube all achieved significant improvements (Ianiro et al., 2019; Xu et al., 2019). However, FMT vs. placebo did not significantly improve IBS symptoms at 12 weeks (RR, 1.19; 95% CI, 0.67–2.13; low; Figure 8; Xu et al., 2019; Elhusein and Fadlalmola, 2022). Furthermore, single FMT via colonoscopy or nasojejunal tube vs. autologous FMT or placebo was associated with improvement (RR, 1.59; 95% CI, 1.06–2.39; low), whereas FMT with multiple-dose capsules vs. capsule excipients as placebo was associated with a lower likelihood of global improvement (RR, 0.54; 95% CI, 0.34–0.85; low; Figure 8; Xu et al., 2019). The response rate was 33.7% for nonoral placebo vs. 67.8% for capsule treatment (Xu et al., 2019). FMT vs. control was associated with a significantly higher IBS Quality of Life score at 4, 8, 12, and 24 weeks but no difference in IBS Severity Scoring System score (Myneedu et al., 2019; Elhusein and Fadlalmola, 2022).

### Psychiatric outcomes

Although the gut-brain axis suggests microbes could affect the central nervous system and neurological diseases, no meta-analysis has explored the clinical efficacy of FMT for these conditions. Available studies evaluating psychiatric outcomes have focused on patients with IBS. For the Fatigue Assessment Scale mental health subscale at 1 month, patients receiving 30 g of FMT vs. placebo showed significant improvement (MD, −1.9; 95% CI, −2.7 to −1.1; low; Figure 9). Similarly, significance was seen at 3 months for patients receiving 60 g of FMT vs. placebo (MD, −2.3; 95% CI, −3.1 to −1.5; low; Figure 9; Green et al., 2020). Anxiety and depression symptoms measured using the Hospital Anxiety and Depression Scale did not differ significantly (Green et al., 2020).

### Functional gut disorders

For functional gut disorders, symptom improvement is also assessed by clinical response and IBS Severity Scoring System score. FMT vs. control showed no significant differences in clinical response (OR, 1.699; 95% CI, 0.273–10.588; low; Figure 8) or IBS Severity Scoring System score (Hedge’s G, 0.282; 95% CI, −1.373 to 1.937; very low; Figure 9), with significant heterogeneity (Green et al., 2020).

### Functional constipation

Combined FMT and laxative therapy was more effective than laxatives alone for improving total effective rate (RR, 1.35; 95% CI, 1.14–1.60; moderate; Figure 8), Bristol Stool Form Scale score (WMD, 1.04; 95% CI, 0.57–1.51; very low), Knowles-Eccersley-Scott-Symptom score (WMD, −5.65; 95% CI, −7.62 to −3.69; low), Wexner score (WMD, −3.25; 95% CI, −5.58 to −0.92; very low), and patient assessment of constipation quality of life score (WMD, −18.56; 95% CI, −26.43 to −10.68; very low; Figure 9; Fang et al., 2021).

### Graft versus host disease

The overall response rate was 64.4% (54/87), including 43.7% complete response and 20.7% partial response. *Clostridioides* emerged as the species with the greatest value for preventing graft versus host disease recurrence. Most patients had no major complications after FMT (Hm et al., 2021).

### Antimicrobial resistance

FMT vs. conventional treatment was associated with a lower rate of failing to achieve remission for decolonization of AMR bacteria (RR, 0.44; 95% CI, 0.20–0.99; low; Figure 8; Dharmaratne et al., 2021). FMT was associated with better clinical remission (RR, 0.37; 95% CI, 0.18–0.79; Supplementary Table S2; Dharmaratne et al., 2021). The one-month decolonization success rate was 0.58 (95% CI, 0.40–0.74; low; Figure 7; Dharmaratne et al., 2021), higher than in a previous study (0.45) (Tavoukjian, 2019).

### Extra-gastrointestinal diseases

#### Obesity and metabolic syndrome

FMT was consistently associated with beneficial changes in early-stage (2–6 weeks) hemoglobin A1c (MD, −1.69 mmol/L; 95% CI, −2.81 to −0.56; low) and early-stage high-density lipoprotein cholesterol (MD, 0.09 mmol/L; 95% CI, 0.02–0.15; low; Figure 9; Proença et al., 2020). FMT was associated with lower early-stage fasting glucose, early-stage triglycerides, early-stage total cholesterol, and Homeostatic Model Assessment for Insulin Resistance score but did not reach significance (Proença et al., 2020). Similarly, FMT was associated with beneficial changes in late-stage (12 weeks) hemoglobin A1c, late-stage low-density lipoprotein cholesterol, late-stage high-density lipoprotein cholesterol, and late-stage triglycerides but did not reach significance (Proença et al., 2020). FMT also seemed associated with beneficial changes in early-stage body mass index, late-stage body mass index reduction, and late-stage hip width reduction but did not reach significance (Proença et al., 2020).

### Disease recurrence and safety

In patients with CDI receiving FMT, an earlier meta-analysis (Li et al., 2016) found overall disease recurrence rates of 5.5% (95% CI, 2.2–10.3%), with early (<90 days) recurrence of 2.9% (95% CI, 0.009–0.058%) and late (≥90 days) recurrence of 1.7% (95% CI, 0.4–4.2%; Figure 10A). Total severe adverse events were 0.65% (95% CI, 0.45–0.89%; Supplementary Table S2; Rapoport et al., 2022). Antibiotic use was associated with most recurrences. Early and late recurrence rates did not differ significantly. Younger patients had significantly lower early recurrence than older patients (*p* = 0.0081; Li et al., 2016). Donor type, delivery route, and number of prior CDI episodes were not associated with statistically significant between-group differences (Li et al., 2016). Frozen FMT resulted in lower incidences of adverse events, severe adverse events, and mortality for CDI than antibiotics, but differences were not statistically significant (Moayyedi et al., 2017; Han et al., 2021). The pooled colectomy rate after FMT for severe and fulminant CDI was 8% (95% CI, 0–24%), with 16% (95% CI, 8–25%) all-cause mortality and 11% (95% CI, 0–30%) severe adverse events (Figure 10A; Tixier et al., 2021). Most adverse events with FMT were mild to moderate and self-limiting (Hui et al., 2019).

For CDI in patients with IBD, recurrence was 19% (95% CI, 13–27%; Supplementary Table S2; Chen et al., 2018). In adults, 26.8% (141/527; 95% CI, 22.5–31.5%) had an IBD flare after FMT and 7.3% (95% CI, 4.7–10.5%) required colectomy (Figure 10A). In children, 10.8% (13/120; 95% CI, 5.7–18.5%) had an IBD flare after FMT and 10.3% (95% CI, 2.1–30.2%) required colectomy (Figure 10A; Tariq et al., 2021).

Overall, 14.9% (95% CI, 10–21%) of patients with IBD had worsening symptoms after FMT (Figure 10A; Qazi et al., 2017). Rates of any adverse events were 29.9% (95% CI, 10.1–61.7%) for chronic pouchitis, 5.8% (95% CI, 1.2–23.5%) for CD, and 36.9% (95% CI, 21.5–55.6%) for UC (Figure 10A; Scaldaferri et al., 2018). Severe adverse events occurred in 5.9% (95% CI, 4.0–8.5%) of patients with ulcerative colitis (Figure 10A; Zhao et al., 2020). Severe adverse event rates did not differ significantly between FMT and control groups (OR, 1.37; 95% CI, 0.63–2.96%; Figure 10B; Tang et al., 2020; Liu et al., 2021).

Of 94 patients with IBS receiving FMT, 29 (30.9%) reported at least one adverse event compared with 25 of 65 (38.5%) receiving placebo (RR, 0.93; 95% CI, 0.45–1.92; low; Figure 10B; Ianiro et al., 2019). No significant between-group differences were found for other adverse symptoms (Myneedu et al., 2019; Xu et al., 2019).

Some patients decolonizing AMR bacteria experienced mild, temporary symptoms like vomiting, diarrhea, abdominal cramps, and bloating (Tavoukjian, 2019). During 6 months of follow-up, four of 18 patients were recolonized, a few spontaneously decolonized, and six died of underlying disease.

For patients with metabolic syndrome and obesity, RCTs found FMT to be safe (Proença et al., 2020). No severe adverse events were reported.

Four studies (Baxter and Colville, 2016; Lin et al., 2016; Lai et al., 2019; Rapoport et al., 2022) reported incidences of specific symptoms after FMT for CDI, IBD, and IBS, including abdominal pain, diarrhea, fever, and procedure- or disease-related complications.

## Discussion

### Principal findings

Previous studies showed gut microbiota relate to disease progression. Carding et al. summarized previous studies elucidating some disease-associated gut microbiota dysbioses (Carding et al., 2015). We have built on this work by summarizing a larger body of current evidence, expanding microbe-disease associations, and identifying key microbes implicated across multiple studies. In 2008, Jia et al. proposed strategies targeting gut microbes, pioneering rationale (Jia et al., 2008). Since then, microbial-targeted therapeutics (MMT) emerged, e.g., FMT, prebiotics, biologics, microbiome-inspired biotherapeutics. Many published studies have examined gut microbiome dysbiosis in disease and the efficacy and safety of FMT for various diseases. In this database analysis, we analyzed associations between 117 gastrointestinal and extra-gastrointestinal diseases and 479 gut microbes. Researchers could use these results to determine disease-microbiota associations and identify key pathogenic bacteria at different taxonomic levels.

In this umbrella review, we identified high-quality meta-analyses assessing FMT for 13 diseases. For rCDI, FMT had high-quality evidence of benefit. However, evidence was very low quality for primary CDI. For IBD, evidence was moderate quality overall, high or moderate for UC, moderate to low or very low for CD, and very low for chronic pouchitis. Low-quality evidence supported FMT for IBD-related CDI and IBS. Low-quality evidence supported FMT for decolonizing AMR bacteria. FMT may help obesity and metabolic syndrome, but evidence was low or very low quality.

In a recent study (Martinez et al., 2022), Hospitalized elderly individuals with CDI had significantly lower abundances of *Lachnospiraceae*, *Ruminococcaceae*, *Blautia* spp., *Prevotella* spp., *Dialister* spp., *Bifidobacterium* spp., *Roseburia* spp., *Anaerostipes* spp., *Faecalibacterium* spp., and *Coprococcus* spp. compared with healthy controls, and higher *Enterococcaceae* and *Enterococcus* spp. Some microbial alterations from this study were observed to be consistent with our radial dendrogram. FMT restores bile acid metabolism and colonic bile acid composition, creating an unfavorable environment for *Clostridioides difficile* spore germination and facilitating rCDI recovery (Weingarden et al., 2014; Brown et al., 2018). The inflammatory to noninflammatory fatty acid ratio decreased, and total fatty acids normalized (Brown et al., 2018). FMT was associated with normalized fecal microbiota and restored alpha diversity (Kelly et al., 2016). Given the current evidence, we recommend FMT be used mainly for CDI patients, especially those with recurrent CDI who stand to benefit the most. This application is endorsed by current guidelines (Appendix 6 showed the current clinical practice guidelines and consensus on FMT). European guideline recommended FMT or bezlotoxumab for rCDI in addition to antibiotics (weak, moderate). However, a multidisciplinary risk assessment was mandatory for FMT, and the preparation and screening of the products should be standardized (weak, moderate; Van Prehn et al., 2021). United Kingdom guideline recommend FMT for rCDI and potential other indications, specifying patient selection, donor screening, preparation, administration, follow-up, and service configuration (strong; Mullish et al., 2018). United Kingdom guideline included an antibiotic strategy for CDI that supported FMT for CDI treating in adults, young people, and children (≥72 h) in community and hospital settings (National Institute for Health and Care Excellence, 2021). United States guideline recommended FMT for multiple rCDI recurrences after appropriate antibiotics (adults: strong, moderate; children: weak, very low; McDonald et al., 2018). We found fresh FMT with multiple infusions via colonoscopy most effective for rCDI. Frozen (−20°C or − 70°C) and lyophilized FMT can also work. Bowel cleansing and aerobic processing may improve outcomes.

For IBD, reduced alpha diversity and dysbiosis are found in active disease. Decreased facultative anaerobes like *E. coli* and increased obligate anaerobes like *Faecalibacterium prausnitzii* occur in CD (Lloyd-Price et al., 2019). *Ruminococcus torques* and *Ruminococcus gnavus* decreased in CD and UC dysbiosis, respectively (Lloyd-Price et al., 2019). For UC, lower gastrointestinal FMT and higher total stool dosage were associated with greater clinical remission. Further study should examine pediatric and adult patients, single or repeated infusions, fresh or frozen FMT, and antibiotics before FMT. For CD, fresh upper gastrointestinal FMT without antibiotics pretreatment may achieve better clinical remission but needs confirmation.

For IBS, especially diarrhea-predominant IBS, decreased *Lactobacillus*, *Bifidobacterium*, and *F. prausnitzii* occur (Liu et al., 2017). After FMT, symptoms improved and the gut microbiome repaired over time (Holvoet et al., 2021). FMT may help IBS but further study should examine routes of administration, donor types, infusion frequency, FMT types, and long-term use.

A trial found FMT significantly decreased AMR bacteria abundance for 2 months, indicating donor engraftment extent directly related to decreased fecal antimicrobial resistance gene carriage (Isgut et al., 2021). FMT was considered to decolonize regimens targeting multidrug-resistant Gram-negative bacteria (Tacconelli et al., 2019). However, guideline argued insufficient evidence to recommend for or against FMT.

For other conditions, evidence is insufficient to recommend for or against FMT. Further study should examine FMT routes of administration, donor types, infusion frequency, and FMT types for clinical use.

Our findings align with and build upon those from several current studies. Borody and Khoruts (2011) proposed FMT may be beneficial across diseases but also noted that treatment protocols require further optimization. Our study aligns with this perspective, emphasizing the need to elucidate the optimal FMT protocols for modulating different diseases. Specifically, Borody and Khoruts (2011) discussed outstanding questions regarding antibiotic pretreatment necessity and dosing frequency, while our study calls for optimizing these details to improve efficacy and summarizes some current evidence. Sorbara and Pamer (2022) explored the potential of FMT for graft-versus-host disease, metabolic syndrome and diabetes, inflammatory bowel disease, among others. Similarly, our study indicates cautious assessment is still needed when applying FMT beyond CDI. Although current literature (Allegretti et al., 2019) also resembles our discussion on the status of FMT for inflammatory bowel disease, hepatological indications, irritable bowel syndrome and functional disorders, metabolic syndrome, and next-generation FMT for recurrent CDI, their conclusion also highlighted FMT is only recommended for recurrent CDI despite many ongoing trials exploring other indications. However, through umbrella review, our study provides evidentiary support for expanding FMT applications and details route of administration, frequency of instillation, fecal preparation, donor type and other parameters. Overall, precision application of FMT for other diseases still requires accumulating high-quality evidence. Our study offers a microbe-disease mapping reference to facilitate designing individualized FMT protocols.

### Potential mechanisms

Multiple mechanisms interact to cause disease and its progression. For many diseases, dysbiosis is an important etiology. Healthy donor feces contain species-rich microbiomes, viromes and mycobiomes that increase diversity, enhance microbial networks and core microbiota, treating symptoms (Jalanka et al., 2016).

**Figure 11:**
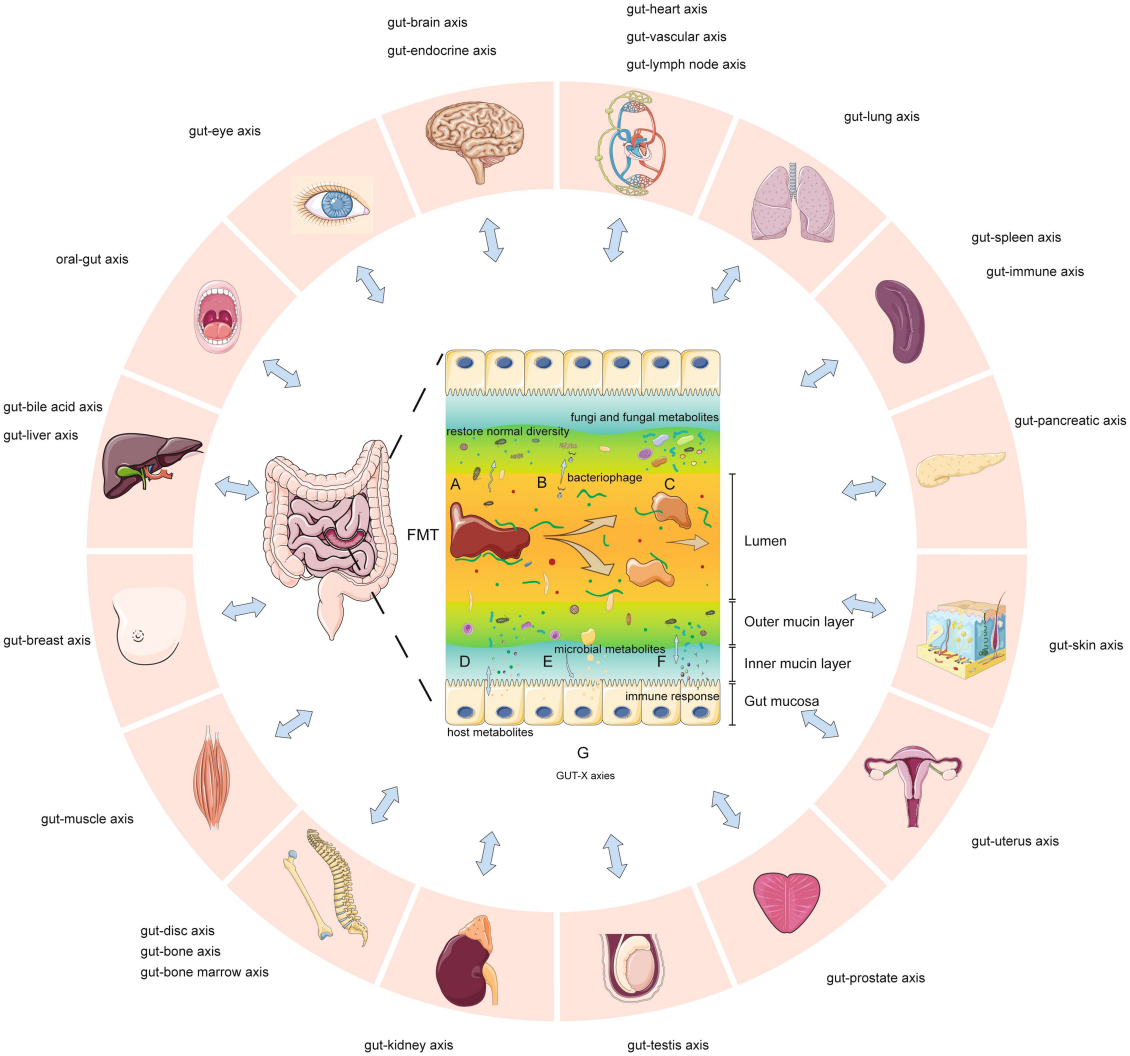
Key mechanisms of fecal microbiota transplantation and potential gut-organ network effects (GUT-X axies). **(A)** Increased microbial diversity. **(B)** Bacteriophage transduction influences transcription and survival of gut bacteria. **(C)** Fungi and fungal metabolites are partly transferred. **(D)** Altered production of metabolites from host. **(E)** Increased production of certain microbial metabolites. **(F)** Altered immune response through metabolites, epitopes or other means. **(G)** Gut microbes interact with multiple organs through structures, metabolites or other means (so-called “GUT-X axies”). FMT may modulate these potential networks.

Recent studies (Hanssen et al., 2021; De Vos et al., 2022; Lam et al., 2022; Leonardi et al., 2022) suggest seven key mechanisms by which FMT may modify human and microbial physiology (Figure 11): A. FMT can rapidly restore normal diversity and enhance microbial networks and core microbes in dysbiosis (Jalanka et al., 2016). B. In CDI, sterile fecal filtrate (containing viruses, metabolites, not bacteria) achieved remission, showing gut viruses’ significance (Ott et al., 2017). Recent studies used sterile feces and fecal viral transplantation to study roles in obesity, diabetes, bacterial overgrowth, necrotizing enterocolitis, post-antibiotic dysbiosis (Lam et al., 2022). C. Reducing *Candida albicans* may improve CDI/IBD outcomes with FMT (Lam et al., 2022). Changes in the gut mycobiome were associated with symptom improvement in IBS/GVHD (Lam et al., 2022). Mucosa-associated fungi activate mucosal immunity (Leonardi et al., 2022). D. FMT alters microbial metabolites acting on hosts (e.g., liver/immune cell metabolism; Hanssen et al., 2021). Metabolites may be from new or existing bacteria. E. Microbial metabolites interact with host cells, activating/inhibiting signaling and health. Bacterial metabolites include short-chain fatty acids (nourishing intestine, altering permeability), peptidoglycan, lipopolysaccharides (De Vos et al., 2022). F. By altering metabolites, antigens, or other mechanisms, FMT influences the host immune response. G. Gut microbiomes connect with organs/systems through structure/metabolites, possibly altering tissue metabolism (GUT-X axies). FMT changes microbiomes, affecting these potential axies. The concept of “GUT-X axies” outlining putative interactions between the gut microbiota and distal organs/systems mediated through structural motifs or bioactive metabolites was formulated based on pertinent publications identified in our PubMed search. This acts as a provisional framework delineating putative microbiota-organ interplay that warrants further verification and interrogation through empirical research to develop additional axies.

### Limitations

This review has some limitations. First, some findings relied on observational studies or small studies, introducing bias and heterogeneity risks and limiting evidence quality. Also, FMT parameters (e.g., delivery route, donor type, dosing frequency, commensal preparation) for non-CDI indications need refinement. Finally, ClinicalTrials.gov lists numerous ongoing FMT studies across diseases; as new evidence emerges, it may alter our conclusions. We will update this review to incorporate impactful new findings when published.

## Conclusion

In summary, gut dysbiosis associates with at least 117 gastrointestinal and extra-gastrointestinal diseases. FMT provides a new modality to reshape the gut microbiome, virome, mycobiome and metabolome, benefitting patients with said diseases. Meta-analyses suggest FMT efficacy and safety in clinical practice, but higher-quality evidence is needed to strengthen recommendations.

Future research should explore gut-disease causality, measure dysbiosis, identify disease mechanisms and FMT’s molecular effects. Improving FMT standardization, innovation and delivery will enhance its efficacy, safety and indications. However, large, rigorous studies are still needed to support expanded use.

Several avenues remain unexplored: (1) Determine if dysbiosis causes or results from certain diseases. New techniques can quantify dysbiosis and its disease-specific nature. (2) Identify disease-associated microbes and FMT’s mechanisms of action. This can illuminate new therapeutic targets and applications (Chen et al., 2022; Lloréns-Rico et al., 2022). (3) Optimize FMT preparation, delivery and dosing through standardization and innovation. Engineered bacterial groups or bacteriophage-based therapy with more precise delivery and control can be developed through FMT (Yang et al., 2022; Airola et al., 2023). Engineered bacteria combined with gut bacteria and functional magnetic nanoparticles provide new ideas for precise regulation of gut bacteria. Using external magnetic field control, engineered bacteria can be successfully and precisely aggregated and colonized in the gut. The external magnetic field also can control the gene expression of the engineered bacteria to remotely regulate genetic functions. This enables more precise and convenient control of engineered bacterial functions. (4) Conduct large RCTs and prospective studies on FMT for complex, non-CDI diseases. Robust evidence will enable guideline recommendations and insurance coverage. (5) Integrate multi-omics datasets to capture the gut microbiome’s immense complexity. Mapping genomes, metabolites and the host interactome may transform disease understanding and precision editing of the gut microbiota.

## Author contributions

XZ: Conceptualization, Data curation, Formal analysis, Investigation, Methodology, Project administration, Resources, Software, Supervision, Validation, Visualization, Writing – original draft, Writing – review & editing. XLu: Conceptualization, Data curation, Formal analysis, Investigation, Methodology, Software, Validation, Visualization, Writing – original draft, Writing – review & editing. LT: Conceptualization, Data curation, Investigation, Validation, Visualization, Writing – review & editing. PY: Data curation, Formal analysis, Investigation, Resources, Validation, Visualization, Writing – review & editing. ML: Data curation, Formal analysis, Investigation, Validation, Visualization, Writing – review & editing. KL: Data curation, Investigation, Visualization, Writing – review & editing. DZ: Data curation, Visualization, Writing – review & editing. CH: Data curation, Writing – review & editing. QS: Data curation, Writing – review & editing. LY: Data curation, Writing – review & editing. ZX: Data curation, Writing – review & editing. JZ: Data curation, Writing – review & editing. ZM: Data curation, Writing – review & editing. JL: Data curation, Writing – review & editing. JWL: Conceptualization, Data curation, Formal analysis, Methodology, Resources, Visualization, Writing – review & editing. YL: Conceptualization, Data curation, Funding acquisition, Methodology, Project administration, Resources, Writing – review & editing. JY: Conceptualization, Data curation, Formal analysis, Methodology, Project administration, Visualization, Writing – review & editing. WM: Conceptualization, Data curation, Formal analysis, Funding acquisition, Investigation, Methodology, Project administration, Resources, Supervision, Validation, Visualization, Writing – review & editing. XLi: Resources, Supervision, Writing – review & editing. YC: Conceptualization, Methodology, Project administration, Resources, Writing – review & editing.
