# Supplementary material for: The gut microbiome dysbiosis and regulation by fecal microbiota transplantation: umbrella review
Source: Front Microbiol. 2023 Nov 3;14:1286429. doi: 10.3389/fmicb.2023.1286429 (PMC10655098; doi:10.3389/fmicb.2023.1286429)
Supplement: Supplementary file 1 [file Presentation_1.pdf]

Supplementary information
Appendix 1. Search Strategy and results
The searched databases include: MEDLINE via PubMed, Embase, the Cochrane Database of Systematic Reviews, Web of Science, China National Knowledge Infrastructure (CNKI), China Biology Medicine (CBM), and Wanfang Data.
Date limits: until October 29, 2021 (Update: June 30, 2022)

1.1 Search strategy for MEDLINE via PubMed
Results: 1052
(((("**meta-analysis**"[Title/Abstract]) OR ("**meta analysis**"[Title/Abstract])) OR ("**meta analyses**"[Title/Abstract])) OR ("**Meta-Analysis**"[Publication Type] OR "**Meta-Analysis as Topic**"[Mesh])) AND (((((((""[Title/Abstract]) OR ("**prebiotic***"[Title/Abstract])) OR ("**synbiotic***"[Title/Abstract])) OR ("**fecal microbiota transplantation**"[Title/Abstract])) OR ("**microbiota transplantation***"[Title/Abstract])) OR (**bacteriotherapy**[Title/Abstract])) OR ("**microbiota transfer***"[Title/Abstract]) OR (((("**Probiotics**"[Mesh]) OR "**Prebiotics**"[Mesh]) OR "**Synbiotics**"[Mesh]) OR "**Fecal Microbiota Transplantation**"[Mesh]))
Filters applied: Journal Article, Humans.

1.2 Search strategy for Embase
Results: 398
('**probiotic agent**'/exp OR '**probiotic**':ti,ab,kw OR '**probiotic agent**':ti,ab,kw OR '**probiotics**':ti,ab,kw OR '**fecal microbiota transplantation**'/exp OR '**fmt (fecal microbiota transplantation)**':ti,ab,kw OR '**imt (intestinal microbiota transplantation)**':ti,ab,kw OR '**bacteriotherapy (feces)**':ti,ab,kw OR '**faecal bacteriotherapy**':ti,ab,kw OR '**faecal enema**':ti,ab,kw OR '**faecal infusion**':ti,ab,kw OR '**faecal matter transplant**':ti,ab,kw OR '**faecal microbial transplant**':ti,ab,kw OR '**faecal microbial transplantation**':ti,ab,kw OR '**faecal microbiome transplant**':ti,ab,kw OR '**faecal microbiome transplantation**':ti,ab,kw OR '**faecal microbiota transplant**':ti,ab,kw OR '**faecal microbiota transplantation**':ti,ab,kw OR '**faecal transplant**':ti,ab,kw OR '**faecal transplantation**':ti,ab,kw OR '**fecal bacterial transplant**':ti,ab,kw OR '**fecal bacterial transplantation**':ti,ab,kw OR '**fecal bacteriotherapy**':ti,ab,kw OR '**fecal enema**':ti,ab,kw OR '**fecal infusion**':ti,ab,kw OR '**fecal instillation**':ti,ab,kw OR '**fecal matter transplant**':ti,ab,kw OR '**fecal matter transplantation**':ti,ab,kw OR '**fecal microbe transplant**':ti,ab,kw OR '**fecal microbial transplant**':ti,ab,kw OR '**fecal microbial transplantation**':ti,ab,kw OR '**fecal microbiome transplant**':ti,ab,kw OR '**fecal microbiome transplantation**':ti,ab,kw OR '**fecal microbiota transplant**':ti,ab,kw OR '**fecal microbiota transplantation**':ti,ab,kw OR '**fecal microbiotal transplant**':ti,ab,kw OR '**fecal microflora transplantation**':ti,ab,kw OR '**fecal transfusion**':ti,ab,kw OR '**fecal transplant**':ti,ab,kw OR '**fecal transplantation**':ti,ab,kw OR '**feces bacteriotherapy**':ti,ab,kw OR '**feces microbe transplantation**':ti,ab,kw OR '**feces microbiota transplantation**':ti,ab,kw OR '**feces microflora transplant**':ti,ab,kw OR '**feces microflora transplantation**':ti,ab,kw OR '**gut microbial transplant**':ti,ab,kw OR '**gut microbial transplantation**':ti,ab,kw OR '**gut microbiome transplant**':ti,ab,kw OR '**gut microbiome transplantation**':ti,ab,kw OR '**gut microbiota transplant**':ti,ab,kw OR '**gut microbiota transplantation**':ti,ab,kw OR '**gut**

microflora transplantation':ti,ab,kw OR **'intestinal microbe transplantation'**:ti,ab,kw OR **'intestinal microbiota transplant'**:ti,ab,kw OR **'intestinal microbiota transplantation'**:ti,ab,kw OR **'intestinal microflora transplantation'**:ti,ab,kw OR **'rectal bacteriotherapy'**:ti,ab,kw OR **'stool enema'**:ti,ab,kw OR **'stool infusion'**:ti,ab,kw OR **'stool instillation'**:ti,ab,kw OR **'stool microbial transplantation'**:ti,ab,kw OR **'stool transplant'**:ti,ab,kw OR **'stool transplantation'**:ti,ab,kw OR **'prebiotic agent'**/exp OR **'prebiotic'**:ti,ab,kw OR **'prebiotic agent'**:ti,ab,kw OR **'prebiotics'**:ti,ab,kw OR **'synbiotic agent'**/exp) AND (**'systematic review'**/exp OR **'review, systematic'**:ti,ab,kw OR **'systematic review'**:ti,ab,kw OR **'systematic review (topic)'**/exp OR **'systematic review (topic)'**:ti,ab,kw OR **'systematic reviews'**:ti,ab,kw OR **'systematic reviews as topic'**:ti,ab,kw OR **'meta analysis'**/exp OR **'analysis, meta'**:ti,ab,kw OR **'meta analysis'**:ti,ab,kw OR **'meta-analysis'**:ti,ab,kw OR **'metaanalysis'**:ti,ab,kw OR **'meta analysis (topic)'**/exp OR **'meta analysis (topic)'**:ti,ab,kw OR **'meta-analysis as topic'**:ti,ab,kw OR **'metaanalyses'**:ti,ab,kw) AND [humans]/lim AND [article]/lim AND [meta analysis]/lim AND [embase]/lim

1.3 Search strategy for the Cochrane Database of Systematic Reviews

Results: 31

ID Search Hits

#1 MeSH descriptor: [**Probiotics**] explode all trees

#2 MeSH descriptor: [**Prebiotics**] explode all trees

#3 MeSH descriptor: [**Synbiotics**] explode all trees

#4 MeSH descriptor: [**Fecal Microbiota Transplantation**] explode all trees

#5 (**probiotic***):ti,ab,kw

#6 (**prebiotic***):ti,ab,kw

#7 (**synbiotic***):ti,ab,kw

#8 (**microbiota transplantation***):ti,ab,kw

#9 #1 OR #2 OR #3 OR #4 OR #5 OR #6 OR #7 OR #8

#10 MeSH descriptor: [**Meta-Analysis as Topic**] explode all trees

#11 (**meta analysis**):ti,ab,kw

#12 (**meta-analysis**):ti,ab,kw

#13 #10 OR #11 OR #12

#14 #9 AND #13

1.4 Search strategy for Web of Science

Results: 595

(TI=(**probiotic***) OR TI=(**prebiotic***) OR TI=(**synbiotic***) OR TI=(**"Microbiota Transplantation*"**)) AND (TI=(**meta analysis**) OR TI=(**meta-analysis**))

Document Types: Articles or Review Articles or Other or Unspecified or Early Access

1.5 Search strategy for CNKI

Results: 179

(TI=(probiotic*) OR TI=(prebiotic*) OR TI=(synbiotic*) OR TI=("Microbiota Transplantation*") OR TI=("fecal microbiota transplantation") AND (TI=(meta analysis) OR TI=(meta-analysis))

1.6 Search strategy for CBM

Results: 166

(TI=(probiotic*) OR TI=(prebiotic*) OR TI=(synbiotic*) OR TI=("Microbiota Transplantation*") OR TI=("fecal microbiota transplantation") AND (TI=(meta analysis) OR TI=(meta-analysis))

1.7 Search strategy for Wanfang Data

Results: 151

(TI=(probiotic*) OR TI=(prebiotic*) OR TI=(synbiotic*) OR TI=("Microbiota Transplantation*") OR TI=("fecal microbiota transplantation") AND (TI=(meta analysis) OR TI=(meta-analysis))

1.8 manual reference check of included articles

Results: 4

Baxter, M. & Colville, A. Adverse events in faecal microbiota transplant: a review of the literature. *The Journal of hospital infection* **92**, 117-127, doi:10.1016/j.jhin.2015.10.024 (2016).

Gough, E., Shaikh, H. & Manges, A. R. Systematic review of intestinal microbiota transplantation (fecal bacteriotherapy) for recurrent Clostridium difficile infection. *Clinical infectious diseases : an official publication of the Infectious Diseases Society of America* **53**, 994-1002, doi:10.1093/cid/cir632 (2011).

Guo, B., Harstall, C., Louie, T., Veldhuyzen van Zanten, S. & Dieleman, L. A. Systematic review: faecal transplantation for the treatment of Clostridium difficile-associated disease. *Alimentary pharmacology & therapeutics* **35**, 865-875, doi:10.1111/j.1365-2036.2012.05033.x (2012).

Sha, S. *et al*. Systematic review: faecal microbiota transplantation therapy for digestive and nondigestive disorders in adults and children. *Alimentary pharmacology & therapeutics* **39**, 1003-1032, doi:10.1111/apt.12699 (2014).

Search strategy for ClinicalTrials.gov (until 1/1/2022)

Results: 416

fecal microbiota transplantation OR intestinal microbiota transplantation OR faecal microbiome transplant OR fecal microbiota therapy OR FMT

Withdrawn: 20

Terminated and Without Results： 16

Without disease condition or non-FMT: 4+6

Included: 370

FMT guideline pubmed (until 4/5/2022)

Results: 64

(((guideline[Title]) OR (consensus[Title])) OR ("Practice Guidelines as Topic"[Mesh] OR "Practice Guideline" [Publication Type])) AND (((("Fecal Microbiota Transplantation"[Mesh]) OR ("fecal microbiota transplantation"[Title/Abstract])) OR ("microbiota transplantation*"[Title/Abstract])) OR ("microbiota transfer*"[Title/Abstract]))

Appendix 2. Table S1. Study characteristics and quality assessment (details).

No.	Author	Pulication year	Country	Disease conditions	Design of included studies	No. of studies	participants	Age (year)	Female(%)	Follow-up	Safety reported	AMSTAR 2
1	Baunwall	2020	Denmark	RCDI	RCTs	9	3768	65.8	66	≥ 8 weeks, w	No	15
					Cohort studies	36						
2	Caldeira	2020	Brazil	IBD	Quasi-experimental studies	27	1169	NR	NR	NR	Yes	12
					RCTs	9						
3	Cao	2018	China	UC	RCTs	4	446	Children and adults	NR	1-12 months, m	No	12
					Cohort studies	14						
4	Chen	2018	China	CDI patients with IBD	Cohort studies	9	346	8-93	56.8	1 w-1 years, y	Yes	12
5	Cheng	2021	China	CD	RCTs	1	107	NR	NR	4 w-15 m	Yes	12
					Cohort studies	7						
					Case reports	4						
6	Cold	2021	Denmark	RCDI	RCTs	3	755	63.7(18-94)	66	39-204 days, d	Yes	14
					Cohort studies	7						
					Case series	7						
					Case reports	1						
7	Colman	2014	America	IBD	Cohort studies	9	70	NR	NR	4 w-1 y	Yes	12
8	Costello	2017	Australia	Active UC	Cohort studies	14	445	NR	NR	1-72 m	Yes	10
					RCTs	4						
9	Dembrovszky	2021	Hungary	multiple RCDI	RCTs	6	310	NR	NR	6-10 w	No	13
10	Dharmaratne	2021	China	AMR	Case reports	7	111	18-89	42.3	3-7 m	Yes	12
					RCTs	2						
					Cohort studies/case-control studies	3						
11	Du	2021	America	RCDI	Case series	12	763	41-77	NR	8-26 w	Yes	14
					RCTs	3						
12	Green	2020	Australia	Diseases other than CDI	RCTs	20	1149	NR	NR	2 w-12 m	Yes	15
					Non-RCTs	3						
					Case-control studies	2						
					Cohort studies	1						
13	Hui	2019	China	RCDI	RCTs	8	541	48-75.7	67.1	8-17 w	Yes	13
14	Ianiro	2019	Italy	IBS	RCTs	5	267	≥18	58.7	≥7 d	Yes	15

15	Ianiro	2018	Italy	RCDI	RCTs<br>Retrospective case series<br>Prospective case series	2<br>9<br>4	1150	67(6-96)	59	≥8 w	No	13
16	Imdad	2018	America	IBD	RCTs	4	277	35-40	41-45	7-12 w	Yes	15
17	Kassam	2013	Canada	CDI	Case series	11	273	65	65	2 w-8 y	Yes	12
18	Lai	2019	China	CDI, IBD	RCTs<br>Non-RCTs	20<br>148	5958	34.25(5-94)	41.65	0-364 m	Yes	12
19	Li	2016	China	CDI	Cohort studies<br>Case series	3<br>15	611	1-95	62.7	1-48 m	Yes	11
20	Liu	2021	China	Active UC	RCTs	5	292	NR	NR	7-12 w	Yes	13
21	Mo	2019	China	UC	Non-RCTs<br>RCTs	19<br>4	536	7-80	NR	1-12 m	Yes	12
22	Moayyedi	2017	Canada	CDAD	RCTs	10	657	NR	NR	8 w-5 m	Yes	12
23	Myneedu	2019	America	IBS	Single arm trial, SATs<br>RCTs	8<br>5	346	18-75	NR	1 w-12 m	Yes	10
24	Narula	2017	Canada	Active UC	RCTs	4	277	NR	NR	7-14 w	Yes	13
25	Paramsothy	2017	America	IBD	Case reports/case series<br>Cohort studies<br>RCTs	14/5<br>34<br>4	555 UC, 83 CD, 23 pouchitis	NR	NR	1 w-13 y	Yes	13
26	Pomares	2021	Spain	CDAD	Clinical trials<br>Cohort studies	12<br>3	1168	≥18	NR	8-13 w	Yes	14
27	Proença	2020	Brazil	Obesity and metabolic syndrome	RCTs	6	154	NR	NR	early 2-18 w	Yes	13
28	Qazi	2017	America	IBD	Case series<br>RCTs<br>Cohort studies	5<br>3<br>21	75<br>92<br>338	NR	NR	4 w-3 y	No	15
29	Quraishi	2017	UK	Recurrent and refractory CDI	RCTs<br>Case series	7<br>30	428<br>1545	5.4-81	NR	NR	Yes	13
30	Ramai	2021	America	CDI	Retrospective studies<br>Prospective studies	13<br>13	1309	7-95	NR	NR	Yes	14
31	Rokkas	2019	Germany	RCDI	RCTs	6	348	NR	NR	8-17 w	Yes	15
32	Scaldaferri	2018	Italy	UC	RCTs	2	NR	NR	NR	NR	Yes	13

33	Shi	2016	China	UC	RCTs<br>Cohort studies<br>Case studies	2<br>15<br>8	234	7-70	NR	4-72w	Yes	14
34	Singh	2021	America	CDI	Open label pilot RCTs<br>Open label RCTs<br>Open label phase trial	7	238	39.7-45.7	NR	NR	Yes	14
35	Sun	2016	China	UC	RCTs<br>Open-label case-control study<br>Cohort studies	2<br>1<br>8	133	NR	NR	1-18 m	Yes	12
36	Tang	2017	China	Recurrent or refractory CDI	RCTs	6	258	11-89	60	10-24 w	Yes	13
37	Tariq	2021	America	RCDI	Prospective studies<br>Retrospective studies	2<br>10	2671	62.5	NR	2-7.7 m	Yes	13
38	Tariq	2019	America	RCDI	RCTs	13	589	NR	NR	8-17 w	Yes	13
39	Tavoukjia n	2019	UK	Antibiotic-resistant bacteria	Case series	3	52	21-80	43	4-24 m	Yes	14
40	Tixier	2021	America	Severe or Fulminant CDI	Case series<br>Cohort studies<br>RCTs	11<br>4<br>1	676	61-82	42-81.3	NR	Yes	15
41	Vuyyuru	2021	India	UC	RCTs	19	5870	NR	NR	8-14 w	Yes	14
42	Xu	2019	America	IBS	RCTs	4	254	NR	NR	6-12 m	Yes	14
43	Zhang	2019	China	UC	RCTs<br>Non-RCTs	4<br>7	277(RCTs)	NR	NR	6-12 w	Yes	11
44	Zhao	2020	China	UC	RCTs<br>Uncontrolled cohort studies<br>Controlled cohort studies	7<br>5<br>25	959	NR	NR	2-72 w	Yes	14
45	Han	2021	China	CDAD	RCTs	8	665	20-95	NR	NR	Yes	11
46	Lin	2016	China	IBD	Non-RCTs	11	104	Children and adults	NR	≥4 w	Yes	11
47	Tang	2017	China	UC	Cohort studies	11	133	7-70	NR	NR	No	10
48	Wu	2021	China	CD	Cohort studies<br>Pilot RCTs	7<br>1	234	Children and adults	NR	8 w-5 y	Yes	13
49	Yang	2021	China	RCDI	RCTs	7	543	NR	NR	NR	Yes	14
50	Ye	2020	china	CD	SATs	7	256	16.2-38	NR	>1 m	Yes	11

51	Yu	2020	China	IBS	RCTs	5	288	33-54	NR	≥4 w	Yes	9
52	Zhang	2019	China	UC	RCTs	4	277	NR	NR	6-12 w	Yes	10
53	Zheng	2016	China	Severe CDI	SATs (Case series)	24	743	5.4-85.3	NR	1-108 m	Yes	11
					Cases							
54	Baxter	2016	China	Adverse events of CDI and IBD	Uncontrolled cohort studies	109	1555	NR	NR	NR	Yes	4
					RCTs							
					RCTs	16						
					Case Series	17						
55	Rapoport	2022	America	Serious adverse events of RCDI			5099	40-84	NA	NR	Yes	8
					Cohort studies	27						
					SATs	1						
					RCTs	11						
					SATs	6						
56	Elhusein	2022	Sudan	IBS			1421	35.8-54.2	59	4w-1y	Yes	12
					Case series	1						
					cohort studies	1						
					RCTs	1						
57	Song	2022	Canada	Severe or Fulminant CDI	Case-control studies	1	240	62.9-74.87	NR	8-65.7w	Yes	13
					case series	8						
58	Wu	2022	China	IBS	RCTs	7	472	NR	NR	6-12m	Yes	13
59	Tang	2020	China	UC	RCTs	7	431	NR	NR	7-48w	Yes	10
60	Fang	2021	China	Functional constipation	RCTs	5	409	44.27-72.10	52.57	4-12w	Yes	12
61	Tariq	2021	America	CDI with IBD	Cohorts	25	598	Children and adults	NR	1m-1.9y	Yes	12
62	Aslam	2021	America	Graft versus host disease	Case reports/case series	11	87	NR	NR	NR	Yes	NA

Note: RCDI, recurrent *Clostridioides difficile* infection; IBD, inflammatory bowel disease; UC, ulcerative colitis; CD, Crohn's disease; AMR, antimicrobial resistance; IBS, irritable bowel syndrome; CDAD, *Clostridioides difficile*-associated diarrhea; GVHD, graft versus host disease; RCTs, randomised clinical trials; SATs, single arm trials; NR, not reported; NA, not applicable.

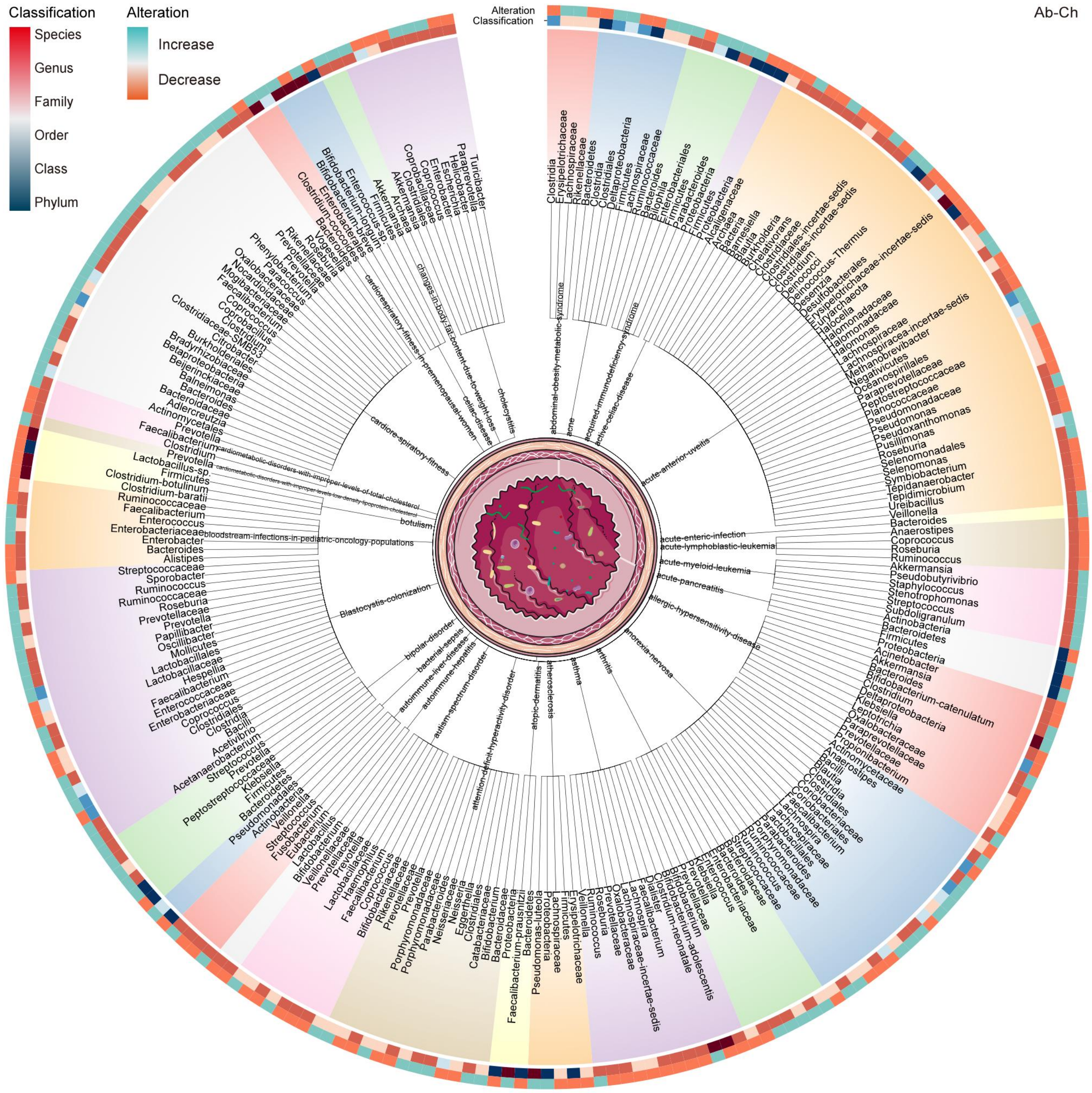
Classification
Species
Genus
Family
Order
Class
Phylum
Alteration
Increase
Decrease
Ab-Ch
Alteration
Classification
abdominal-obesity-metabolic-syndrome
acne
acquired-immunodeficiency-syndrome
active-celiac-disease
acute-anterior-uveitis
acute-enteric-infection
acute-lymphoblastic-leukemia
acute-myeloid-leukemia
acute-pancreatitis
allergic-hypersensitivity-disease
anorexia-nervosa
arthritis
asthma
atherosclerosis
atopic-dermatitis
attention-deficit-hyperactivity-disorder
autism-spectrum-disorder
autoimmune-hepatitis
autoimmune-liver-disease
bacterial-sepsis
bipolar-disorder
Blastocystis-colonization
bloodstream-infections-in-pediatric-oncology-populations
botulism
cardiometabolic-disorders-with-improper-levels-low-density-lipoprotein-cholesterol
cardiometabolic-disorders-with-improper-levels-of-total-cholesterol
cardiore-spiratory-fitness
cardiorespiratory-fitness-in-premenopausal-women
celiac-disease
changes-in-body-fat-content-due-to-weight-loss
cholecystitis

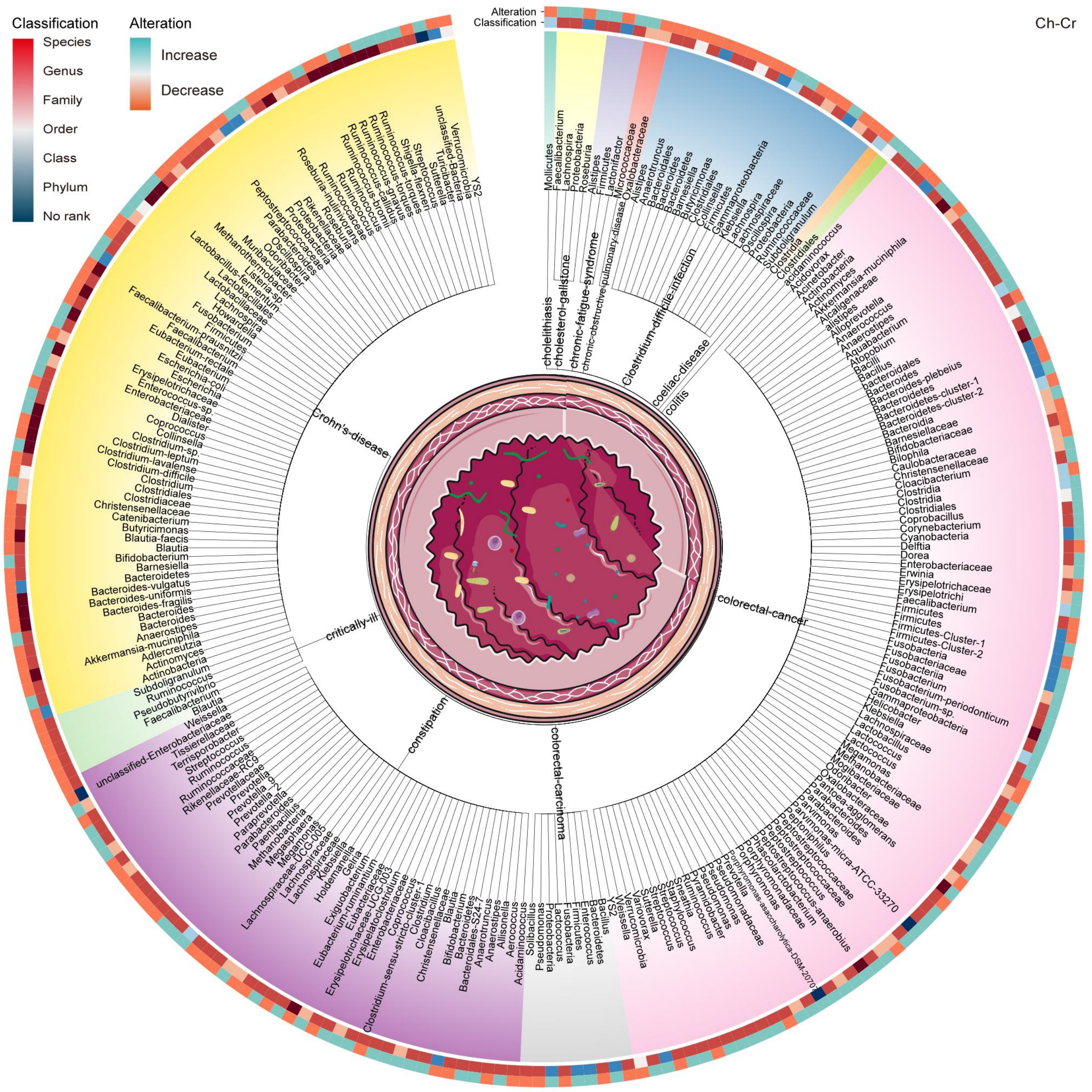

Classification
Species
Genus
Family
Order
Class
Phylum
No rank
Alteration
Increase
Decrease
Alteration
Classification
Ch-Cr
cholelithiasis
cholesterol-gallstone
chronic-fatigue-syndrome
chronic-obstructive-pulmonary-disease
Clostridium-difficile-infection
coeliac-disease
colitis
colorectal-cancer
colorectal-carcinoma
constipation
critically-ill
Crohn's-disease

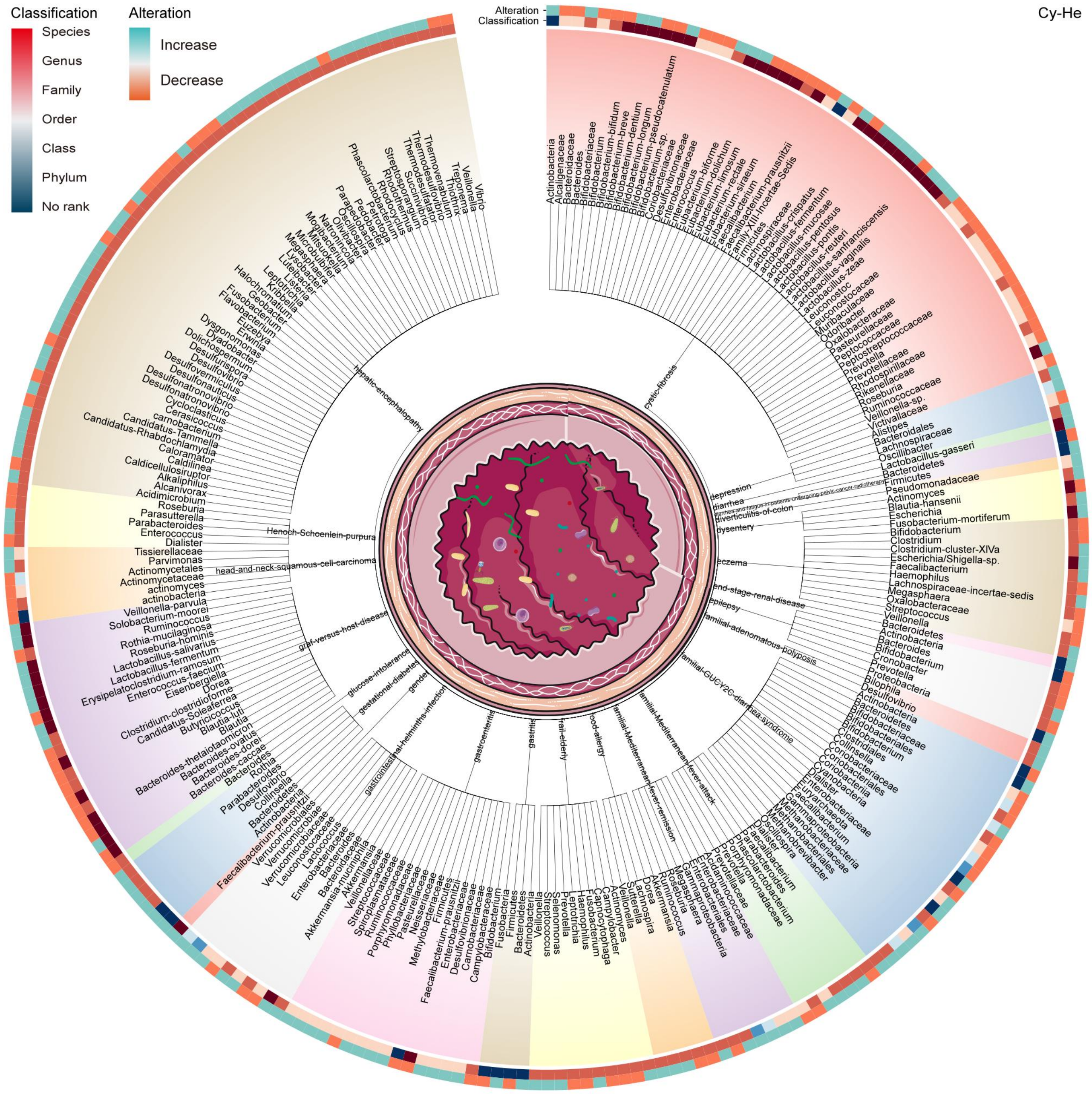

Classification
Species
Genus
Family
Order
Class
Phylum
No rank
Alteration
Increase
Decrease
Cy-He
Alteration
Classification
cystic-fibrosis
depression
diarrhea
diverticulitis-of-colon
dysentery
eczema
end-stage-renal-disease
epilepsy
familial-adenomatous-polyposis
familial-GUCY2C-diarrhea-syndrome
familial-Mediterranean-fever-attack
familial-Mediterranean-fever-remission
food-allergy
frail-elderly
gastritis
gastroenteritis
gastrointestinal-helminths-infection
gestational-diabetes
glucose-intolerance
graf-versus-host-disease
head-and-neck-squamous-cell-carcinoma
Henoch-Schoenlein-purpura
hepatic-encephalopathy

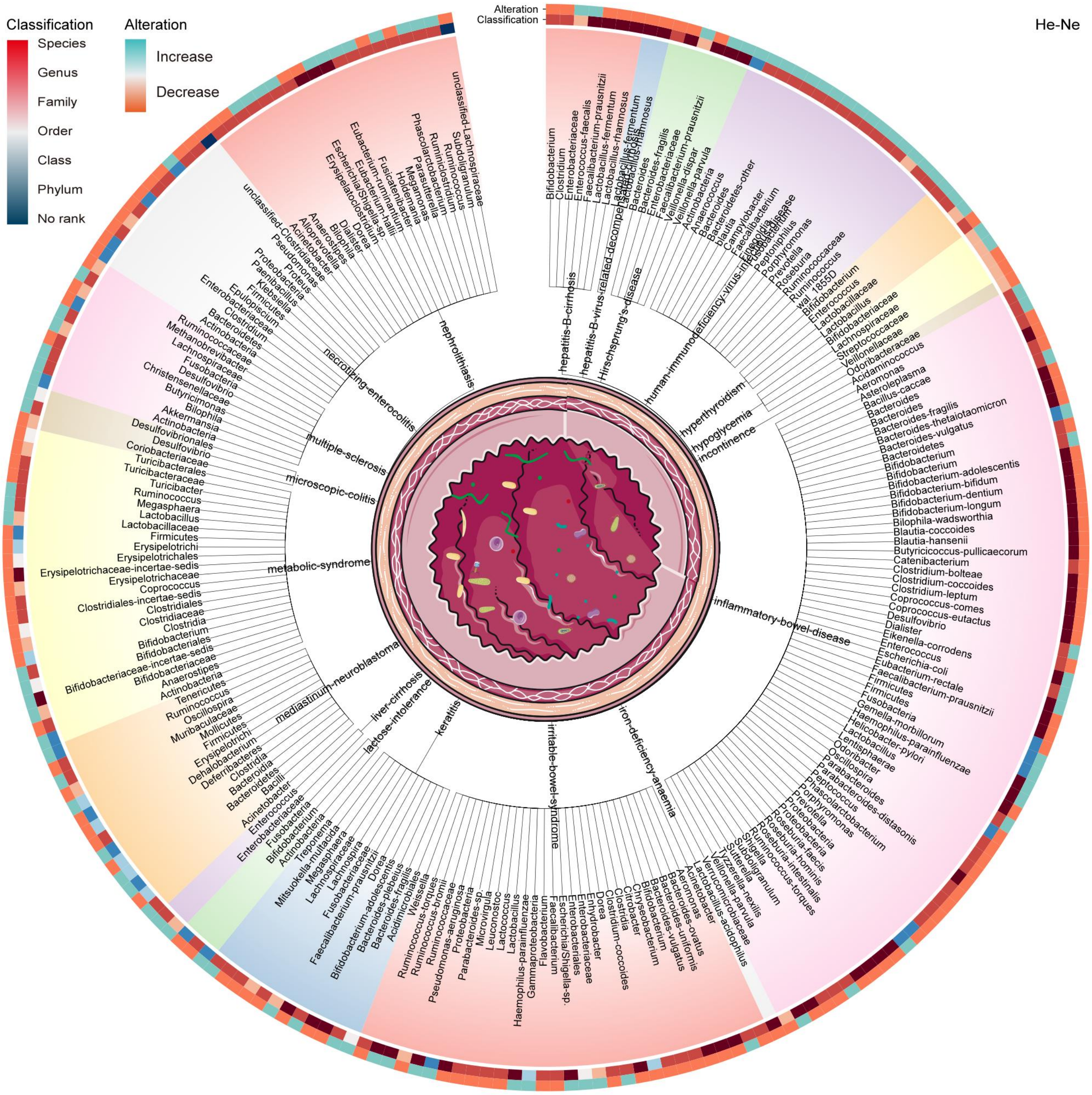

Classification
Species
Genus
Family
Order
Class
Phylum
No rank
Alteration
Increase
Decrease
He-Ne
Alteration
Classification
nephrolithiasis
hepatitis-B-cirrhosis
hepatitis-B-virus-related-decompensated-cirrhosis
Hirschsprung's-disease
human-immunodeficiency-virus-infected-disease
hyperthyroidism
hypoglycemia
incontinence
inflammatory-bowel-disease
iron-deficiency-anaemia
irritable-bowel-syndrome
keratitis
lactose-intolerance
liver-cirrhosis
mediastinum-neuroblastoma
metabolic-syndrome
microscopic-colitis
multiple-sclerosis
necrotizing-enterocolitis

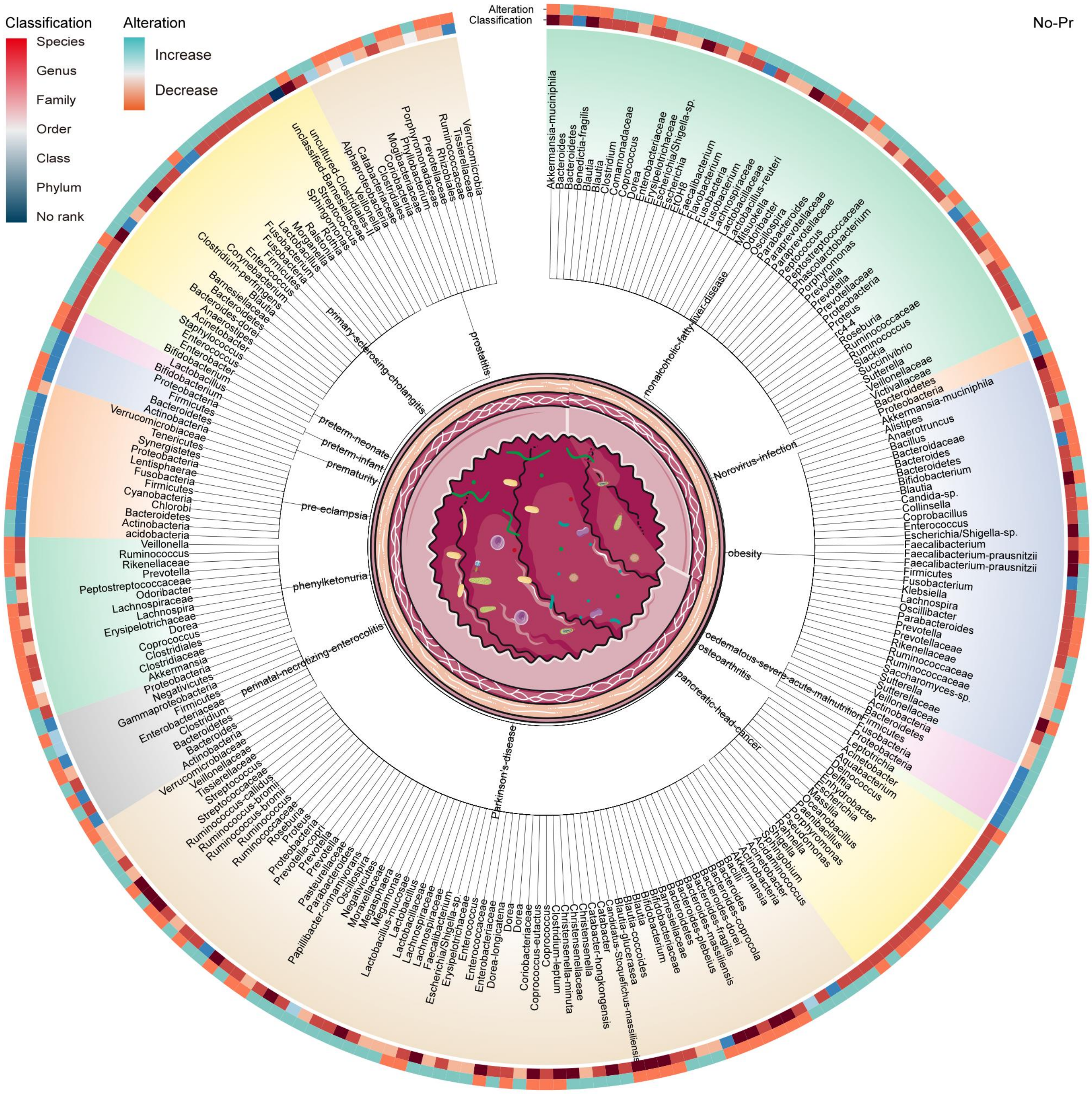
Classification
Species
Genus
Family
Order
Class
Phylum
No rank
Alteration
Increase
Decrease
Alteration
Classification
No-Pr
prostatitis
primary-sclerosing-cholangitis
preterm-neonate
preterm-infant
prematurity
pre-eclampsia
phenylketonuria
perinatal-necrotizing-enterocolitis
Parkinson's-disease
pancreatic-head-cancer
osteoarthritis
oedematous-severe-acute-malnutrition
obesity
Norovirus-infection
nonalcoholic-fatty-liver-disease

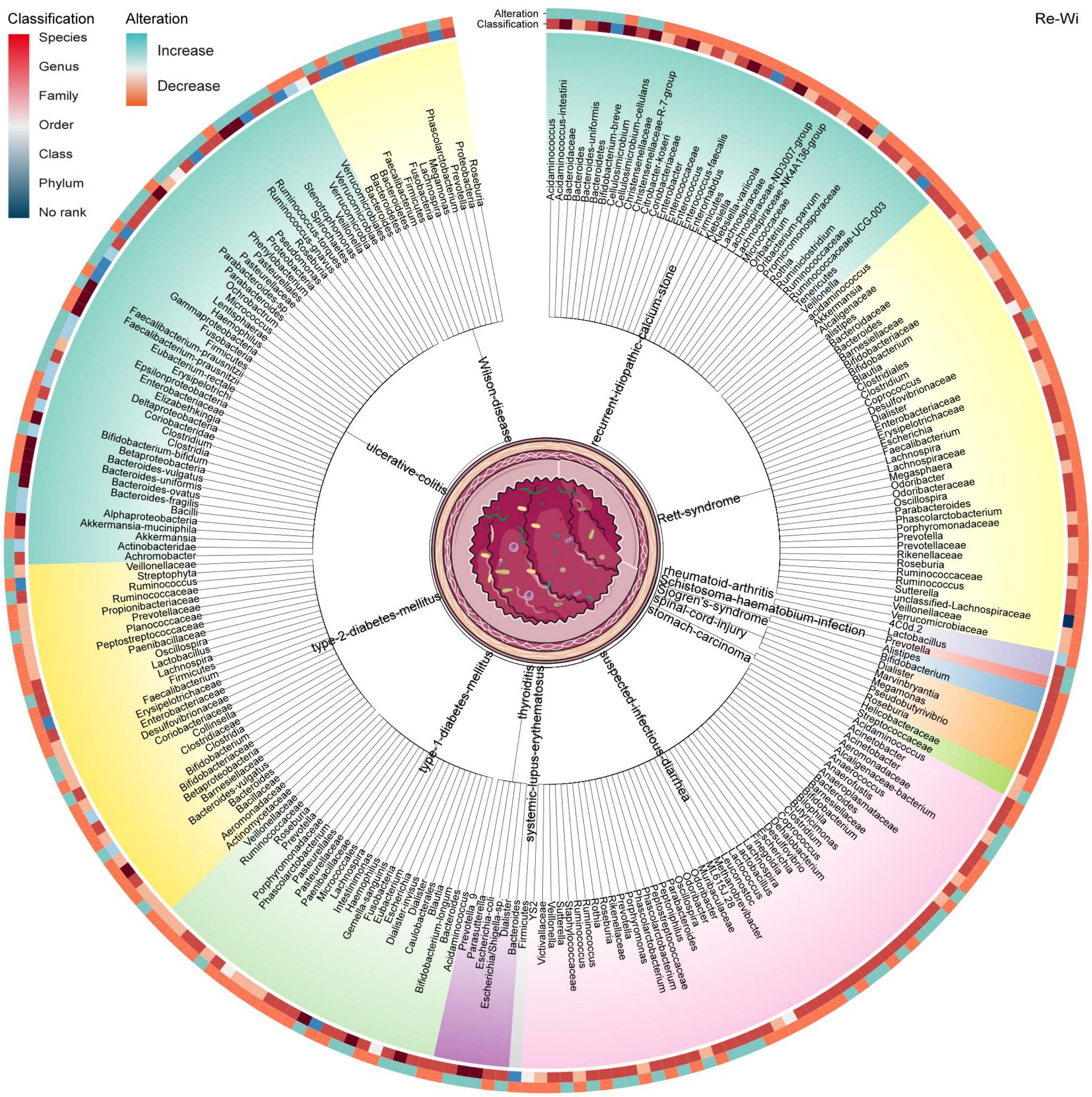

Appendix 3. Figure S1. Dysbiosis of the gut microbes in disease conditions, clustered by disease. Note: Due to the needs of the code written, some spaces in the names of the microbes have been replaced with hyphens.

4C-Ba

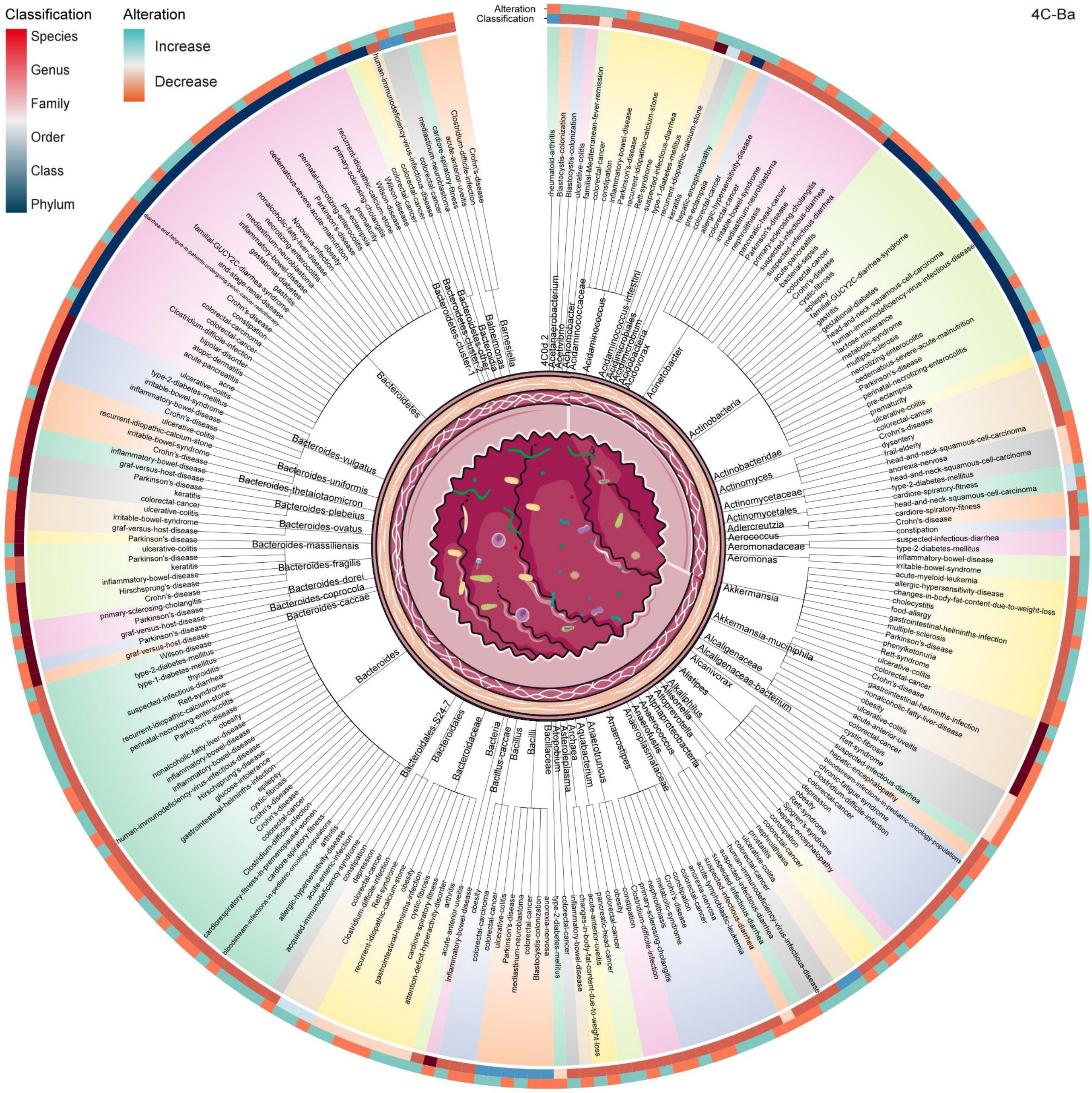
Classification
Species
Genus
Family
Order
Class
Phylum
Alteration
Increase
Decrease
Alteration
Classification
Bacteroidetes
Bacteroides-vulgatus
Bacteroides-uniformis
Bacteroides-thetaiotaomicron
Bacteroides-plebeius
Bacteroides-ovatus
Bacteroides-massiliensis
Bacteroides-fragilis
Bacteroides-dorei
Bacteroides-coprocola
Bacteroides-caccae
Bacteroides
Bacteroidales-S24-7
Bacteroidales
Bacteroidaceae
Bacteria
Bacillus-caccae
Bacillus
Bacilli
Bacillaceae
Atopobium
Asteroleplasma
Arthrobacter
Anaerotruncus
Anaerostipes
Anaeroplasmataceae
Alkaliphilus
Alistipes
Alcanivorax
Alcaligenaceae-bacterium
Alcaligenaceae
Alcaligenes
Akkermansia-muciniphila
Akkermansia
Aeromonas
Aeromonadaceae
Aerococcus
Adlercreutzia
Actinomycetales
Actinomycetaceae
Actinomyces
Actinobacteridae
Actinobacteria
Acinetobacter
Acidovorax
Acidaminococcus
Acidaminococcaceae
Acetivibrio
Acetanaerobacterium
4C0d-2
Barnesiella

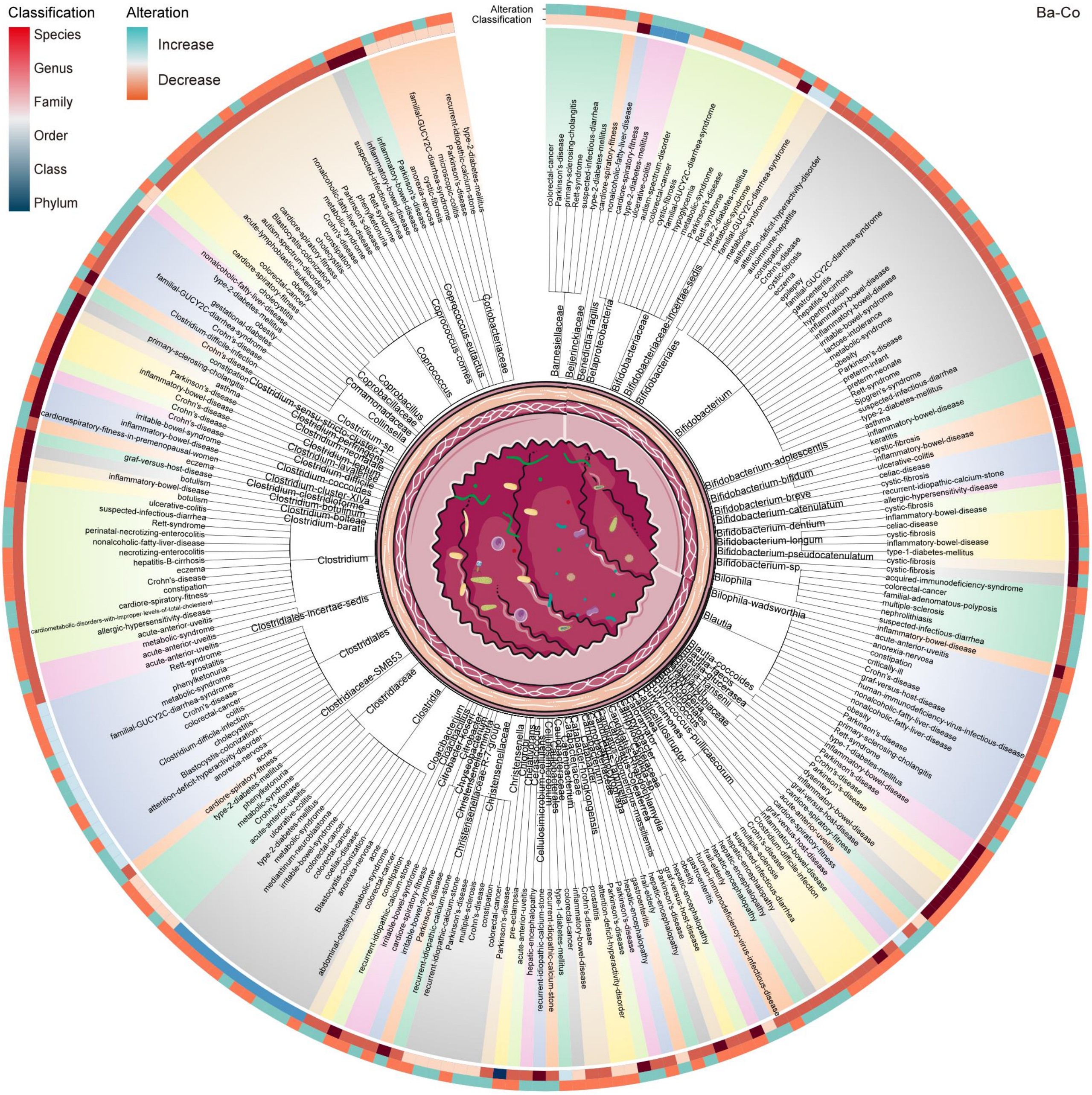

Classification
Species
Genus
Family
Order
Class
Phylum
Alteration
Increase
Decrease
Alteration
Classification
Ba-Co
Barnesiellaceae
Beijerinckiaceae
Betaproteobacteria
Bifidobacteriaceae
Bifidobacteriaceae-incertae-sedis
Bifidobacteriales
Bifidobacterium
Bifidobacterium-adolescentis
Bifidobacterium-bifidum
Bifidobacterium-breve
Bifidobacterium-catenulatum
Bifidobacterium-dentium
Bifidobacterium-longum
Bifidobacterium-pseudocatenulatum
Bifidobacterium-sp.
Bilophila
Bilophila-wadsworthia
Blautia
Coriobacteriaceae
Coprococcus-eutactus
Coprococcus-comes
Coprococcus
Coprobacillus
Coprobacillaceae
Comamonadaceae
Collinsella
Clostridium-sp.
Clostridium-perfringens
Clostridium-difficile
Clostridium-coccoides
Clostridium-cluster-XIVa
Clostridium-clostridioforme
Clostridium-botulinum
Clostridium-bolteae
Clostridium-baratii
Clostridium
Clostridiales-incertae-sedis
Clostridiales
Clostridiaceae-SMB53
Clostridiaceae
Clostridia
Christensenellaceae

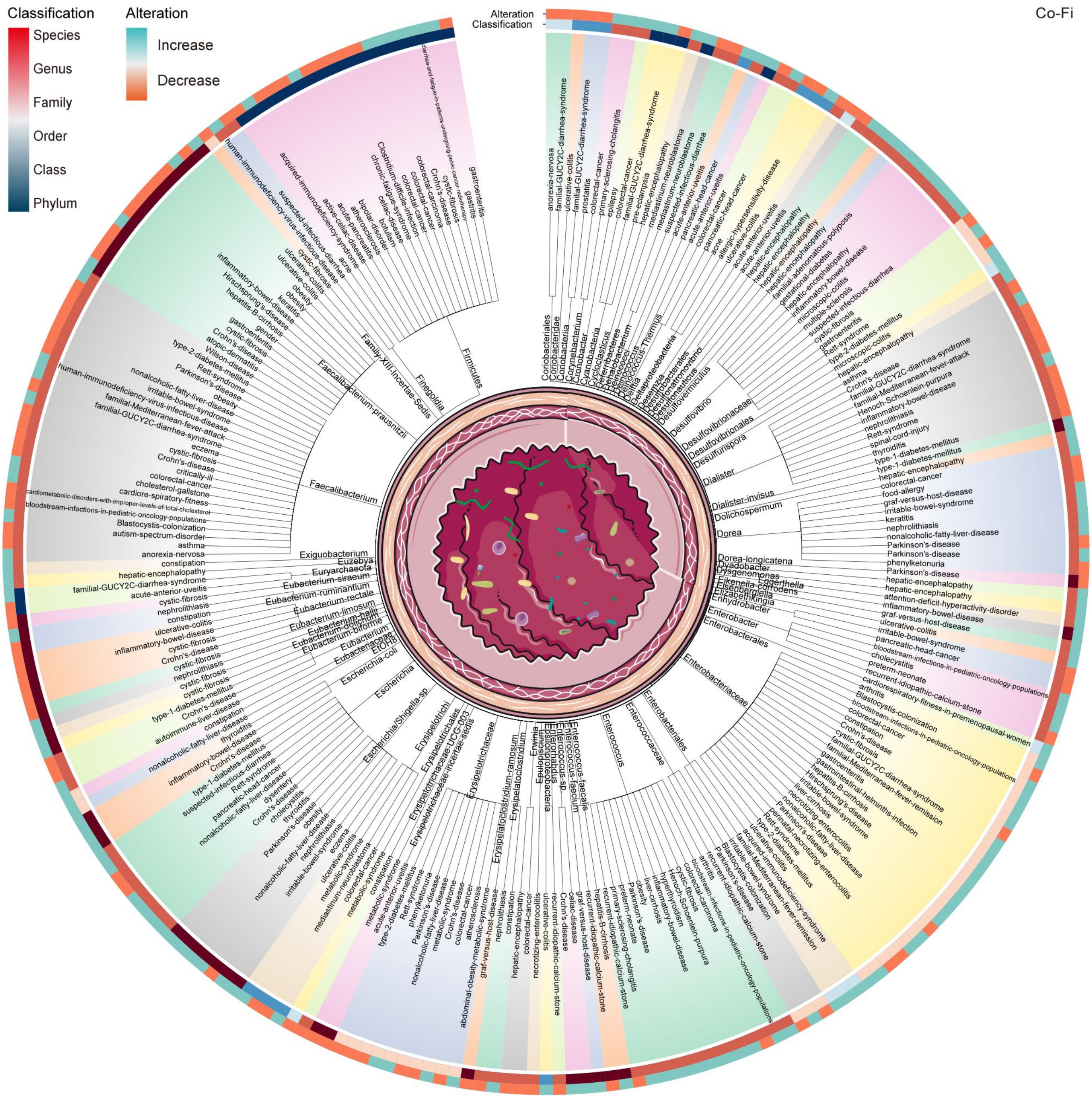
Classification
Species
Genus
Family
Order
Class
Phylum
Alteration
Increase
Decrease
Alteration
Classification
Co-Fi

Fi-Mu

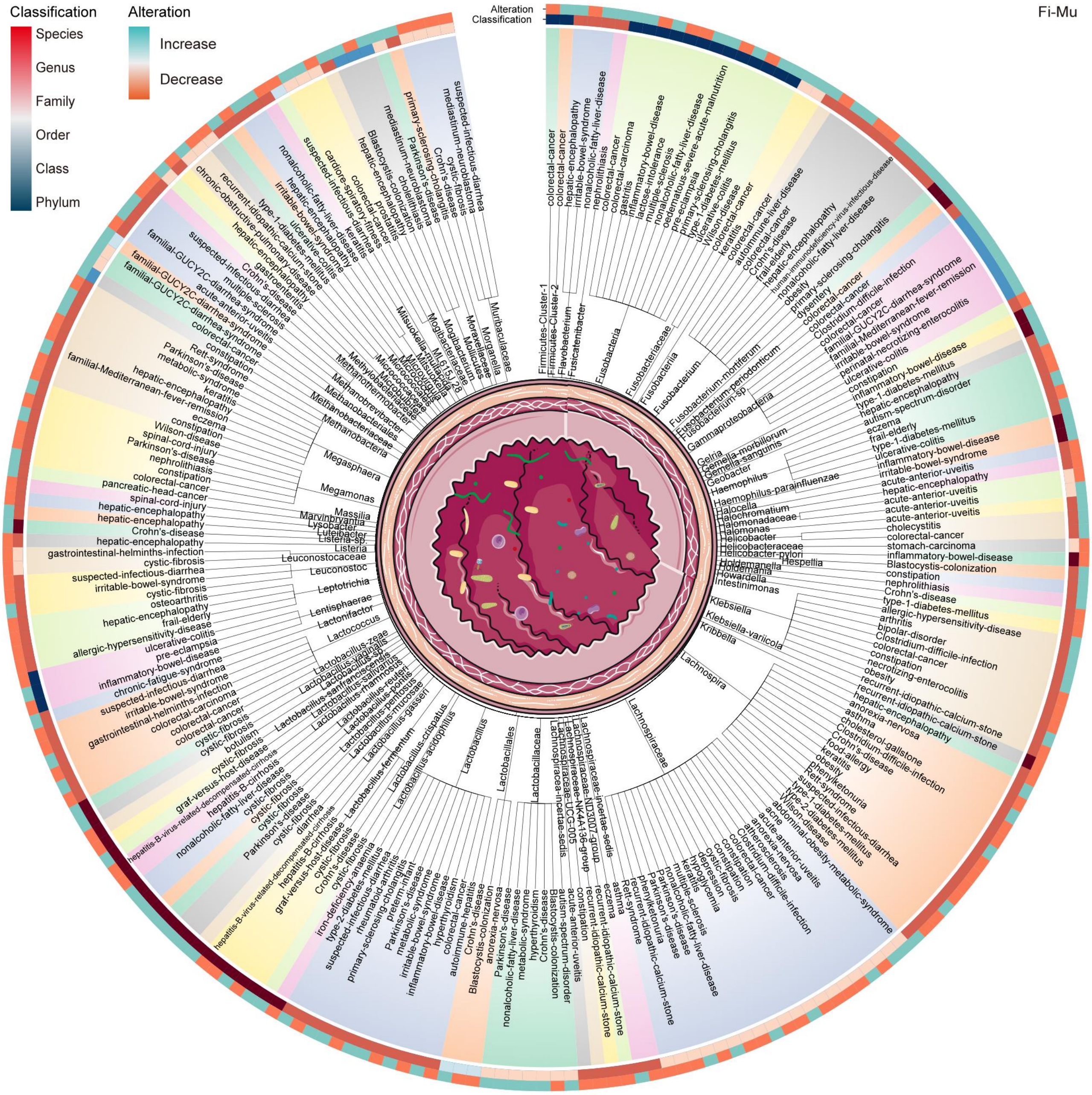

Classification
Species
Genus
Family
Order
Class
Phylum
Alteration
Increase
Decrease
Alteration
Classification
Fusobacteria
Fusobacteriaceae
Fusobacterium
Fusobacterium-nucleatum
Lachnospiraceae
Lachnospira
Lactobacillus
Lactobacillaceae
Lactobacillales
Helicobacter
Helicobacter-pylori
Klebsiella
Methanobrevibacter
Megasphaera
Megamonas
Leuconostoc
Listeria

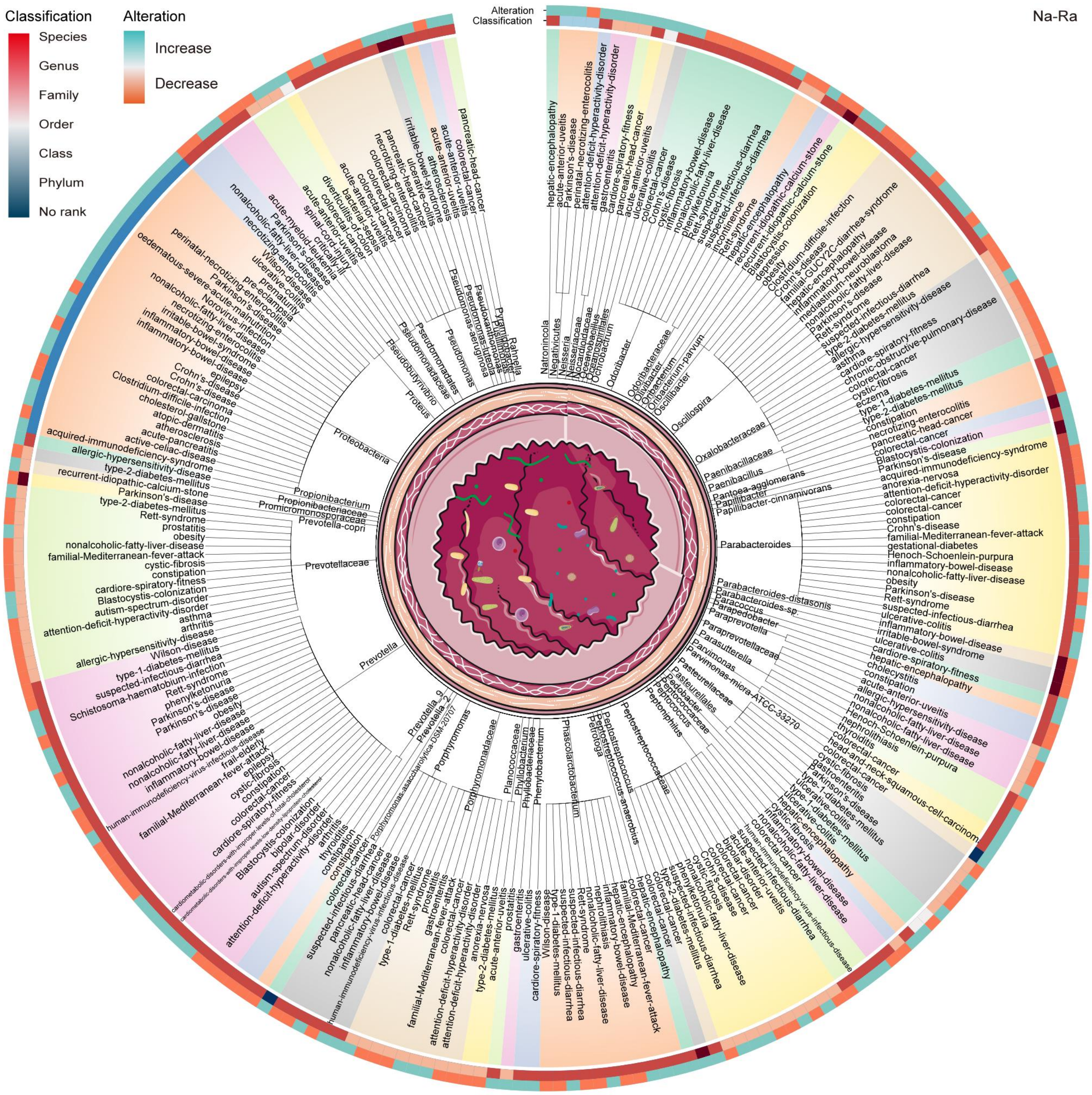

Classification
Species
Genus
Family
Order
Class
Phylum
No rank
Alteration
Increase
Decrease
Na-Ra
Alteration
Classification

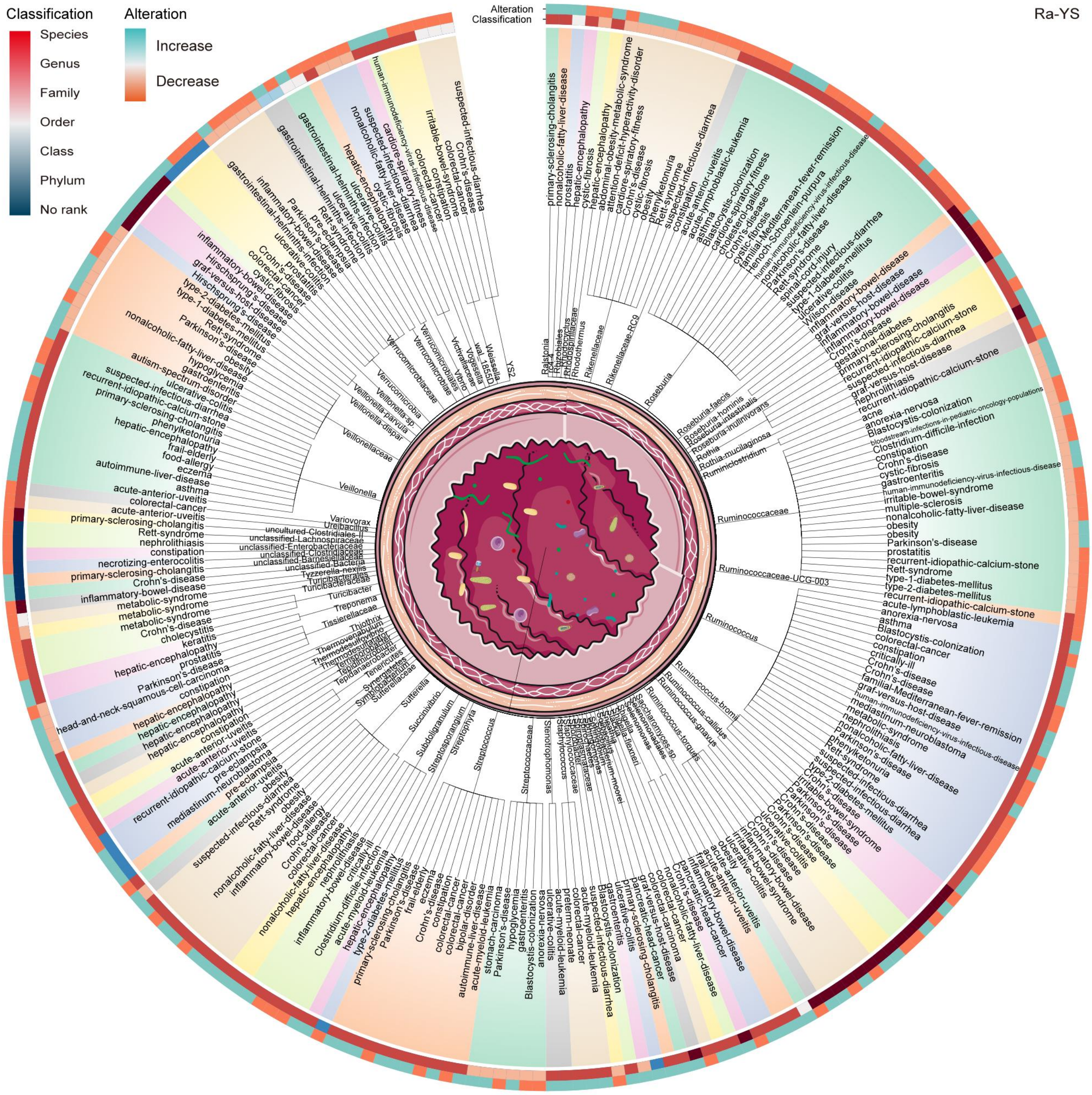

Appendix 4. Figure S2. Dysbiosis of the gut microbes and associated diseases, clustered by the gut microbes. Note: Due to the needs of the code written, some spaces in the names of the microbes have been replaced with hyphens.

Appendix 5. Table S2. Subgroup analysis of FMT in disease conditions.

Study	Pulication year	Disease conditions	Overall/ subgroup	Subgroup setting	Treatment	Control	Event/T	Event/C	Event	Participants	No. of studies	Study design	Outcomes	Measure	Estimate	95% CI	I2 (%)	P
**CDI**																		
21	2019	CDI	Overall		FMT				NR	4609	132	RCTs/non-RCTs	Primary cure rate	Event rate	0.8772	0.8443-0.9074	NR	NR
			Overall		FMT				NR	4609	132	RCTs/non-RCTs	Final cure rate	Event rate	0.9556	0.9388-0.9705	NR	NR
			Deliver way	Upper gastrointestinal tract	FMT				NR	824	37	RCTs/non-RCTs	Primary cure rate	Event rate	0.8806	0.8305-0.9248	NR	NR
				Upper gastrointestinal tract	FMT				NR	824	37	RCTs/non-RCTs	Final cure rate	Event rate	0.9290	0.8925-0.9600	NR	NR
				Lower gastrointestinal tract	FMT				NR	2359	63	RCTs/non-RCTs	Primary cure rate	Event rate	0.8897	0.8422-0.9314	NR	NR
				Lower gastrointestinal tract	FMT				NR	2359	63	RCTs/non-RCTs	Final cure rate	Event rate	0.9665	0.9434-0.9848	NR	NR
				both upper and lower GI and/or unknown routes	FMT				NR	1426	32	RCTs/non-RCTs	Primary cure rate	Event rate	0.8392	0.7492-0.9158	NR	NR
				both upper and lower GI and/or unknown routes	FMT				NR	1426	32	RCTs/non-RCTs	Final cure rate	Event rate	0.9545	0.9185-0.9824	NR	NR
23	2016	CDI	Age	Younger (<65 years)	FMT				NR	NR	9	Cohorts/case series	Primary cure rate	Event rate	0.994	0.969-100.0	NR	0.0003
				Older (≥65 years)	FMT				NR	NR	10	Cohorts/case series	Primary cure rate	Event rate	0.870	0.816-0.916	NR	NR
			Donor type	Related	FMT				NR	NR	10	Cohorts/case series	Primary cure rate	Event rate	0.926	0.862-0.974	NR	0.8225
				Random	FMT				NR	NR	5	Cohorts/case series	Primary cure rate	Event rate	0.926	0.862-0.974	NR	NR
			No. of prior CDI episodes	Less prior episodes of CDI (<4)	FMT				NR	NR	8	Cohorts/case series	Primary cure rate	Event rate	0.940	0.838-0.993	NR	0.8629
				More prior episodes of CDI (≥4)	FMT				NR	NR	8	Cohorts/case series	Primary cure rate	Event rate	0.950	0.824-100.0	NR	NR
24	2021	CDI	Method of administration	Colonoscopy	FMT				NR	483	16	retrospective studies +prospective studies	overall cure rate	Event rate	0.95	0.92-0.97	0.16	0.28
				Capsule	FMT				NR	301	4	retrospective studies +prospective studies	overall cure rate	Event rate	0.92	0.89-0.95	0.07	0.36
				Enema	FMT				NR	252	4	retrospective studies +prospective studies	overall cure rate	Event rate	0.87	0.83-0.90	0	0.65
				Nasogastric tube OR nasoduodenal tube	FMT				NR	165	6	retrospective studies +prospective studies	Cure rate	Event rate	0.78	0.72-0.84	0	0.6
22	2021	CDI	CDI type	Primary and RCDI	Single FMT	MT	75/107	52/131		238	7	Open label RCT +Open label pilot RCT+Open label phase trial	Clinical response rate	RR	1.52	0.90-2.58	77	0.0002
				Primary and RCDI	Repeat FMT	MT	84/107	52/131		238	7	Open label RCT +Open label pilot RCT+Open label phase trial	Clinical response rate	RR	1.68	0.96-2.94	82	<0.00001
				RCDI	FMT	MT	63/76	30/97		173	4	Open label RCT +Open label pilot RCT+Open label phase trial	Clinical response rate	RR	2.41	1.20-4.83	78	0.003
				Primary CDI	FMT	MT	21/31	22/34		65	3	Open label RCT +Open label pilot RCT+Open label phase trial	Clinical response rate	RR	1.00	0.72-1.39	0	0.50
26	2017	recurrent and refractory CDI	Overall		FMT				NR	1973	37	RCT+case series	response rate	Event rate	0.92	0.89-0.94	0.59	0
			infusion times	Single infusions	FMT				NR	1697	34	RCT+case series	response rate	Event rate	0.84	0.79-0.89	0.84	0
				Multiple infusions	FMT				NR	1973	37	RCT+case series	response rate	Event rate	0.92	0.89-0.94	0.59	0
			Deliver way, infusion times	lower GI-delivered, multiple infusions	FMT				NR	1035	22	RCT+case series	response rate	Event rate	0.95	0.92-0.97	48	0.1

				upper GI-delivered, multiple infusions	FMT				NR	507	11	RCT+case series	response rate	Event rate	0.88	0.82-0.94	0.39	0.9
				lower GI-delivered, single infusions	FMT				NR	1059	23	RCT+case series	response rate	Event rate	0.87	0.79-0.94	0.895	0
				upper GI-delivered, single infusions	FMT				NR	447	9	RCT+case series	response rate	Event rate	0.81	0.73-0.88	0.25	0.23
			Bacterial fluid status/infusion times	fresh FMT, multiple infusions	FMT				NR	1400	30	RCT+case series	response rate	Event rate	0.92	0.89-0.95	0.54	0
				frozen FMT, multiple infusions	FMT				NR	192	4	RCT+case series	response rate	Event rate	0.93	0.87-0.97	0.19	0.29
				fresh FMT, single infusions	FMT				NR	1410	30	RCT+case series	response rate	Event rate	0.85	0.79-0.90	0.83	0
				frozen FMT, single infusions	FMT				NR	164	3	RCT+case series	response rate	Event rate	0.68	0.47-0.86	0.77	0.01
86	2017	recurrent and refractory CDI	colonic lavage or not before FMT	colonic lavage before FMT	FMT		56		NR	56	3	RCTs+cohort studies	first effective rate	Event rate	0.63	0.56-0.69	0	0.71
				not colonic lavage before FMT	FMT		212		NR	212	3	RCTs+cohort studies	first effective rate	Event rate	0.73	0.61-0.84	0	0.5
				colonic lavage before FMT	FMT		56		NR	56	3	RCTs+cohort studies	second effective rate	Event rate	0.96	0.93-0.98	0	0.71
				not colonic lavage before FMT	FMT		212		NR	212	3	RCTs+cohort studies	second effective rate	Event rate	0.92	0.84-0.99	0	0.5
30	2020	RCDI	Delivery method (Superiority of one delivery method) FMT	Overall (CDAD week 8, Single FMT)	FMT				NR	2743	41	Observational studies	Anticipated absolute effect	Event rate	0.84	0.80-0.87	85	<0.01
			the 8 week effect for single faecal microbiota transplantation (FMT)	Lower GI endoscopy	FMT				NR	1654	28	Observational studies	Anticipated absolute effect	Event rate	0.90	0.87-0.92	44	<0.01
				Upper administration	FMT				NR	277	6	Observational studies	Anticipated absolute effect	Event rate	0.75	0.70-0.80	0	0.82
				Capsule	FMT				NR	426	8	Observational studies	Anticipated absolute effect	Event rate	0.83	0.78-0.87	22	0.26
				Enema	FMT				NR	397	5	Observational studies	Anticipated absolute effect	Event rate	0.57	0.42-0.70	83	<0.01
			Delivery method (Superiority of one delivery method)	Overall (CDAD week 8, Repeat FMT)	FMT				NR	1513	22	Observational studies	Anticipated absolute effect	Event rate	0.92	0.89-0.94	53	<0.01
			the week 8 effect for repeat faecal microbiota transplantation (FMT)	Lower GI endoscopy	FMT				NR	725	12	Observational studies	Anticipated absolute effect	Event rate	0.95	0.92-0.98	42	0.06
				Upper administration	FMT				NR	114	5	Observational studies	Anticipated absolute effect	Event rate	0.86	0.78-0.92	0	0.65
				Capsule	FMT				NR	327	5	Observational studies	Anticipated absolute effect	Event rate	0.89	0.84-0.94	36	0.18
				Enema	FMT				NR	347	3	Observational studies	Anticipated absolute effect	Event rate	0.88	0.83-0.93	37	0.2
			Frequency	CDAD week 8, Single FMT	FMT	Vancomycin	NR	NR	NR	151	4	RCTs	Anticipated absolute effect	RR	1.95	0.93-4.0	NR	NR
				CDAD week 8, Repeat FMT	FMT	Vancomycin	NR	NR	NR	117	3	RCTs	Anticipated absolute effect	RR	3.33	2.2-5.0	NR	NR
			Frequency	Single	FMT	Vancomycin	76	75	NR	151	4	RCTs	Anticipated absolute effect	Risk Difference MH, Random	0.35	0.03-0.67	81	<0.01

				Repeat	FMT	Vancomycin	60	61	NR	121	3	RCTs	Anticipated absolute effect	Risk Difference MH, Random	0.65	0.52-0.78	0	0.98
			Frequency	Single	FMT				NR	2937	43	Observational studies/RCTs	Clinical effect week 8	Event rate	0.84	0.80-0.88	0.86	NR
				Repeat	FMT				NR	1855	24	Observational studies/RCTs	Clinical effect week 8	Event rate	0.91	0.89-0.94	53	NR
30	2021	RCDI	Frequency	Single	FMT				596	723	16	RCTs/Cohorts/case reports/case series	Primary cure rate	Event rate	0.85	0.82-0.88	0	0.57
				Repeat	FMT				667	743	17	RCTs/Cohorts/case reports/case series	Cure rate	Event rate	0.93	0.88-0.96	60	<0.01
			Donor type	Single-donor capsules	FMT				NR	696	13	RCTs/Cohorts/case reports/case series	Primary cure rate	Event rate	0.833	0.802-0.864	NR	0.21
				Multi-donor capsules	FMT				NR	22	2	RCTs/Cohorts/case reports/case series	Primary cure rate	Event rate	0.911	0.793-1.000	NR	NR
				Lyophilisation	FMT				NR	158	3	RCTs/Cohorts/case reports/case series	Primary cure rate	Event rate	0.805	0.743-0.867	NR	0.26
				No lyophilisation	FMT				NR	565	13	RCTs/Cohorts/case reports/case series	Primary cure rate	Event rate	0.846	0.811-0.889	NR	NR
			Storage temperature	(-80°C)	FMT				NR	581	11	RCTs/Cohorts/case reports/case series	Primary cure rate	Event rate	0.824	0.789-0.859	NR	NR
				(-70°C)	FMT				NR	70	1	RCTs/Cohorts/case reports/case series	Primary cure rate	Event rate	0.901	0.832-0.971	NA	NA
				(-20°C)	FMT				NR	9	1	RCTs/Cohorts/case reports/case series	Primary cure rate	Event rate	0.889	0.684-1.000	NA	NA
				(4°C)	FMT				NR	63	2	RCTs/Cohorts/case reports/case series	Primary cure rate	Event rate	0.826	0.733-0.919	NR	NR
				Bowel cleansing	FMT				NR	88	3	RCTs/Cohorts/case reports/case series	Primary cure rate	Event rate	0.879	0.812-0.947	NR	0.14
				No bowel cleansing	FMT				NR	448	10	RCTs/Cohorts/case reports/case series	Primary cure rate	Event rate	0.822	0.787-0.856	NR	NR
				Aerobic processing	FMT				NR	551	11	RCTs/Cohorts/case reports/case series	Primary cure rate	Event rate	0.844	0.804-0.885	NR	0.47
				Anaerobic processing	FMT				NR	104	2	RCTs/Cohorts/case reports/case series	Primary cure rate	Event rate	0.803	0.727-0.879	NR	NR
			Duration of treatment	One day	FMT				NR	250	7	RCTs/Cohorts/case reports/case series	Primary cure rate	Event rate	0.823	0.776-0.869	NR	0.31
				Two days	FMT				NR	464	11	RCTs/Cohorts/case reports/case series	Primary cure rate	Event rate	0.85	0.817-0.883	NR	NR
				Three days	FMT				NR	9	1	RCTs/Cohorts/case reports/case series	Primary cure rate	Event rate	0.889	0.684-1.000	NA	NA
			Study type	RCT	FMT				NR	101	3	RCTs/Cohorts/case reports/case series	Primary cure rate	Event rate	0.887	0.826-0.948	NR	0.12
				Non RCT	FMT				NR	633	13	RCTs/Cohorts/case reports/case series	Primary cure rate	Event rate	0.805	0.755-0.856	NR	NR
			Total number of capsules	Below 30	FMT				NR	170	5	RCTs/Cohorts/case reports/case series	Primary cure rate	Event rate	0.794	0.734-0.853	NR	0.11
				30	FMT				NR	407	8	RCTs/Cohorts/case reports/case series	Primary cure rate	Event rate	0.842	0.807-0.877	NR	NR
				Above 30	FMT				NR	146	6	RCTs/Cohorts/case reports/case series	Primary cure rate	Event rate	0.878	0.826-0.930	NR	NR
27	2021	multiple recurrent	network meta-analysis		Vancomycin+FMT						6	RCTs	sustained clinical cure	SUCRA	0.83	NR	NR	NR

		CDI											
				Fidaxomicin		6	RCTs	sustained clinical cure	SUCRA	0.6	NR	NR	NR
				Clostridioides difficile immune whey		6	RCTs	sustained clinical cure	SUCRA	0.52	NR	NR	NR
				Metronidazol		6	RCTs	sustained clinical cure	SUCRA	0.51	NR	NR	NR
				Vancomycin		6	RCTs	sustained clinical cure	SUCRA	0.47	NR	NR	NR
				Tolevamer		6	RCTs	sustained clinical cure	SUCRA	0.3	NR	NR	NR
				Vancomycin+lavage		6	RCTs	sustained clinical cure	SUCRA	0.27	NR	NR	NR
28	2019	RCDI	network meta-analysis	donor fecal microbiota transplantation	vancomycin	2	RCTs	Cure rate	OR	15.42	4.47-53.18	0	0.49
				vancomycin	vancomycin+bowel lavage	1	RCTs	Cure rate	OR	1.48	0.60-8.50	NR	0.25
				donor fecal microbiota transplantation	vancomycin+bowel lavage	1	RCTs	Cure rate	OR	14.44	2.39-87.4	NR	NA
				donor fecal microbiota transplantation	autologous fecal microbiota transplantation	1	RCTs	Cure rate	OR	6.00	1.13-31.94	NR	NA
				vancomycin+donor fecal microbiota transplantation	vancomycin	2	RCTs	Cure rate	OR	2.11	0.09-48.36	88.45	0.003
				donor fecal microbiota transplantation	placebo	1	RCTs	Cure rate	OR	1.88	0.79-4.45	NR	NA
				vancomycin	fidaxomicin	1	RCTs	Cure rate	OR	2.17	0.48-9.86	NR	NA
				vancomycin+donor fecal microbiota transplantation	fidaxomicin	1	RCTs	Cure rate	OR	4.86	1.43-16.5	NR	NA
				vancomycin+bowel lavage	fidaxomicin	NR	RCTs	Cure rate	OR	0.97	0.16-8.32	NR	NR
				vancomycin	fidaxomicin	NR	RCTs	Cure rate	OR	1.06	0.32-3.49	NR	NR
				vancomycin	vancomycin+bowel lavage	NR	RCTs	Cure rate	OR	1.15	0.22-5.27	NR	NR
				vancomycin+donor fecal microbiota transplantation	autologous fecal microbiota transplantation	NR	RCTs	Cure rate	OR	1.51	0.15-26.70	NR	NR
				placebo	vancomycin+donor fecal microbiota transplantation	NR	RCTs	Cure rate	OR	2.05	0.37-15.08	NR	NR
				donor fecal microbiota transplantation	placebo	NR	RCTs	Cure rate	OR	2.29	1-5.12	NR	NR
				placebo	autologous fecal microbiota transplantation	NR	RCTs	Cure rate	OR	2.92	0.39-28.61	NR	NR
				autologous fecal microbiota transplantation	vancomycin	NR	RCTs	Cure rate	OR	3.00	0.2-22.06	NR	NR
				autologous fecal microbiota transplantation	fidaxomicin	NR	RCTs	Cure rate	OR	3.26	0.16-30.93	NR	NR
				autologous fecal microbiota transplantation	vancomycin+bowel lavage	NR	RCTs	Cure rate	OR	3.34	0.22-41.15	NR	NR
				vancomycin+donor fecal microbiota transplantation	vancomycin	NR	RCTs	Cure rate	OR	4.29	.51-12.22	NR	NR
				vancomycin+donor fecal microbiota transplantation	fidaxomicin	NR	RCTs	Cure rate	OR	4.68	1.65-13.25	NR	NR
				donor fecal microbiota transplantation	vancomycin+donor fecal microbiota transplantation	NR	RCTs	Cure rate	OR	4.69	1.04-25.22	NR	NR
				vancomycin+donor fecal microbiota transplantation	vancomycin+bowel lavage	NR	RCTs	Cure rate	OR	5.25	0.60-27.77	NR	NR
				donor fecal microbiota transplantation	autologous fecal microbiota transplantation	NR	RCTs	Cure rate	OR	6.42	1.28-57.74	NR	NR
				placebo	vancomycin	NR	RCTs	Cure rate	OR	9.22	2.21-39.86	NR	NR
				placebo	fidaxomicin	NR	RCTs	Cure rate	OR	9.57	1.54-70.82	NR	NR
				placebo	vancomycin+bowel lavage	NR	RCTs	Cure rate	OR	9.8	1.66-60.57	NR	NR

					donor fecal microbiota transplantation	vancomycin					NR	RCTs	Cure rate	OR	20.02	7.05-70.03	NR	NR
					donor fecal microbiota transplantation	fidaxomicin					NR	RCTs	Cure rate	OR	22.01	4.38-109.63	NR	NR
					donor fecal microbiota transplantation	vancomycin+bowel lavage					NR	RCTs	Cure rate	OR	22.77	4.34-131.63	NR	NR
33	2021	CDAD	Overall		FMT				860	1003	15	Clinical trials/cohorts	Treatment effctiveness	SMD	0.82	0.75-0.89	20	NR
			Deliver way		Colonoscopy	Enema			NR	NR	NR	Clinical trials/cohorts	Treatment effctiveness	SMD	-0.74	-0.90–0.58	NR	NR
					Colonoscopy	Capsule			NR	NR	NR	Clinical trials/cohorts	Treatment effctiveness	SMD	0.44	0.20-0.69	NR	NR
					Colonoscopy	esophagogastroduodenoscopy			NR	NR	NR	Clinical trials/cohorts	Treatment effctiveness	SMD	-2.28	-2.63–1.93	NR	NR
31	2021	RCDI	Overall		Fresh	Control	203/251	153/236		487	8	RCTs	Cure rate	OR	6.7	1.64-27.43	82	<0.00001
			Frequency		Single FMT	Repeat FMT	101/151	145/151		302	4	RCTs	Cure rate	OR	0.09	0.04-0.21	0	0.49
87	2021	RCDI	Overall	Capsules	FMT				619	763	15	RCTs/case series	Efficacy	Event rate	0.82	0.76-0.87	64	<0.01
			Bacterial fluid status	Frozen	FMT				163	201	5	RCTs/case series	Efficacy	Event rate	0.86	0.78-0.92	NR	NR
				Lyophilized	FMT				387	460	7	RCTs/case series	Efficacy	Event rate	0.82	0.76-0.87	NR	NR
			Stool type	Capsules	FMT	Colonoscopy	107/116	151/173		289	4	RCTs/case series	Efficacy	RR, relative rate	1.01	0.95-1.08	NR	0.40
34	2019	RCDI	Frequency	Single infusions	FMT		610		439	610	13	RCTs	clinical resolution rate	Event rate	0.76	0.64-0.86	0.91	0.001
				Multiple infusions	FMT		610		539	610	13	RCTs	clinical resolution rate	Event rate	0.89	0.84-0.94	0.8	0.001
			Study type	Overall in Controlled Trials With Control Arm	FMT	placebo or standard-of-care antibiotic	155	61	139	155	6	RCTs	clinical resolution rate	Event rate	0.68	0.54-0.81	0.79	0.001
				Overall in Trials With No Comparator Group	FMT		394		300	394	8	RCTs	clinical resolution rate	Event rate	0.83	0.71-0.94	0.93	0.001
25	2022	Severe or Fulminant CDI	Overall		FMT				211	240	10	RCTs/Case-control studies/case series	successful resolution	Event rate	0.88	0.83-0.91	0	0.45
57	2021	Severe or Fulminant CDI	Mortality		FMT		444			444	11	case series+RCTs+cohort studies	pooled mortality rate	Event rate	0.16	0.08-0.25	0.78	0.01
			Colectomy		FMT		425			425	9	case series+RCTs+cohort studies	pooled colectomy rate	Event rate	0.08	0-0.24	0.89	0.01
			Overall		FMT		150			150	6	case series+RCTs+cohort studies	serious adverse event rate	Event rate	0.11	0-0.30	0.87	0.01
56	2021	CDAD	Bacterial fluid status	Freeze-dried	FMT	Control	86	84		170	2	RCT	Event rate	OR	1.76	0.66-4.72	0	0.43
				Fresh	FMT	Control	8	4		12	1	RCT	Event rate	OR	2.14	0.63-7.33	NA	NA
				Frozen	FMT	Control	44	28		72	5	RCT	Event rate	OR	1.62	0.92-2.86	0	0.85
54	2022	RCDI	Overall		FMT				NR	5099	61	RCTs/Cohorts/SATs/case series	Serious adverse events	Overall rates	0.0065	0.0045-0.0089	NR	<0.001
23	2016	CDI	Overall		FMT				NR	611	17	Cohorts/case series	Recurrence rate	Event rate	0.055	0.022-0.103	NR	NR
			Age	Younger (<65 years)	FMT				NR	330	9	Cohorts/case series	Late recurrence rate	Event rate	0.040	0.010-0.089	NR	0.4509
				Older (≥65 years)	FMT				NR	NR	10	Cohorts/case series	Late recurrence rate	Event rate	0.022	0.005-0.051	NR	NR
			Donor type	Related	FMT				NR	NR	10	Cohorts/case series	Late recurrence rate	Event rate	0.029	0.005-0.072	NR	0.3065
				Random	FMT				NR	NR	5	Cohorts/case series	Late recurrence rate	Event rate	0.014	0.000-0.051	NR	NR
			Delivery route	Upper gastrointestinal delivery	FMT				NR	NR	5	Cohorts/case series	Late recurrence rate	Event rate	0.005	0.000-0.047	NR	0.2040
				Lower gastrointestinal delivery	FMT				NR	NR	10	Cohorts/case series	Late recurrence rate	Event rate	0.035	0.009-0.078	NR	NR
			No. of prior CDI episodes	Less prior episodes of CDI (<4)	FMT				NR	NR	8	Cohorts/case series	Late recurrence rate	Event rate	0.037	0.009-0.081	NR	0.7860
				More prior episodes of CDI	FMT						8	Cohorts/case series	Late recurrence rate	Event rate	0.021	0.002-0.059	NR	NR

				(≥4)														
58	2019	RCDI	Method of administration	Fresh	FMT	Van/placebo	6/93	39/84		177	4	RCTs	Recurrence rate	RR	0.17	0.08-0.37	0	0.69
				Enema	FMT	Van/frozen	22/103	20/103		206	2	RCTs	Recurrence rate	RR	1.07	0.64-1.80	0	0.45
				Colonoscopy	FMT	Frozen/capsule	2/77	6/77		154	2	RCTs	Recurrence rate	RR	0.42	0.05-3.94	43	0.18
			Overall		FMT	Control	30/273	65/264		537	8	RCTs	Recurrence rate	RR	0.38	0.16-0.87	87.1	0.004
			Frequency		Multiple	Single	5/120	21/104		224	5	RCTs	Recurrence rate	RR	0.24	0.10-0.58	0	0.64
**IBD**																		
35	2020	IBD	Overall		FMT	Placebo	179	176	NR	355	6	RCTs	Clinical remission	RR M-H, Random	1.79	1.12-2.56	45	0.11
			Overall		FMT	Placebo	140	137	NR	277	4	RCTs	Clinical response	RR M-H, Random	1.68	1.04-2.72	55	0.08
			Stool type	Blank	FMT				NR	NR	2	quasi-experimental studies	Clinical remission	Event rate	0.35	0.053-0.838	NR	0.59
				Capsules	FMT				NR	NR	2	quasi-experimental studies	Clinical remission	Event rate	0.665	0.427-0842	NR	0.17
				Fresh	FMT				NR	NR	21	quasi-experimental studies/RCT	Clinical remission	Event rate	0.291	0.203-0.399	NR	0
				Fresh/frozen	FMT				NR	NR	3	quasi-experimental studies/RCT	Clinical remission	Event rate	0.572	0.259-0.836	NR	0.673
				Frozen	FMT				NR	NR	6	quasi-experimental studies/RCT	Clinical remission	Event rate	0.442	0.339-0.550	NR	0.291
				Overall	FMT				NR	NR	34	quasi-experimental studies/RCT	Clinical remission	Event rate	0.406	0.337-0.479	NR	0.012
			Donor type	Blank	FMT				NR	NR	2	quasi-experimental studies	Clinical remission	Event rate	0.104	0.030-0.301	NR	0.001
				Inespecific	FMT				NR	NR	7	quasi-experimental studies/RCT	Clinical remission	Event rate	0.364	0.210-0.552	NR	0.155
				Relative/acquaintance	FMT				NR	NR	9	quasi-experimental studies/RCT	Clinical remission	Event rate	0.248	0.115-0.454	NR	0.019
				Universal	FMT				NR	NR	16	quasi-experimental studies/RCT	Clinical remission	Event rate	0.451	0.333-0.576	NR	0.443
				Overall	FMT				NR	NR	34	quasi-experimental studies/RCT	Clinical remission	Event rate	0.36	0.281-0.448	NR	0.002
			IBD subtype	Any IBD	FMT				NR	NR	3	quasi-experimental studies/RCT	Clinical remission	Event rate	0.41	0.027-0.947	NR	0.826
				Chronic pouchitis	FMT				NR	14	2	quasi-experimental studies	Clinical remission	Event rate	0.074	0.010-0.382	NR	0.015
				Crohn's disease	FMT				NR	NR	10	quasi-experimental studies/RCT	Clinical remission	Event rate	0.476	0.309-0.649	NR	0.792
				Ulcerative colitis	FMT				NR	NR	22	quasi-experimental studies/RCT	Clinical remission	Event rate	0.35	0.260-0.452	NR	0.004
				Overall	FMT				NR	NR	37	quasi-experimental studies/RCT	Clinical remission	Event rate	0.368	0.289-0.454	NR	0.003
			Stool type	Blank	FMT				NR	NR	1	quasi-experimental studies	Clinical response	Event rate	0.1	0.006-0.674	NR	0.14
				Capsules	FMT				NR	NR	2	quasi-experimental studies	Clinical response	Event rate	0.949	0.713-0.993	NR	0.004
				Fresh	FMT				NR	NR	21	quasi-experimental studies/RCT	Clinical response	Event rate	0.521	0.414-0.627	NR	0.699
				Fresh/frozen	FMT				NR	NR	2	quasi-experimental studies/RCT	Clinical response	Event rate	0.566	0.261-0.828	NR	0.688
				Frozen	FMT				NR	NR	7	quasi-experimental studies/RCT	Clinical response	Event rate	0.528	0.416-0.638	NR	0.625
				Overall	FMT				NR	NR	33	quasi-experimental studies/RCT	Clinical response	Event rate	0.536	0.462-0.609	NR	0.339
			Donor type	Blank	FMT				NR	NR	2	quasi-experimental studies	Clinical response	Event rate	0.368	0.053-0.858	NR	0.65
				Inespecific	FMT				NR	NR	7	quasi-experimental	Clinical response	Event rate	0.589	0.439-0.724	NR	0.245

												studies/RCT						
				Relative/acquaintance	FMT				NR	NR	8	quasi-experimental studies/RCT	Clinical response	Event rate	0.371	0.215-0.560	NR	0.178
				Universal	FMT				NR	NR	16	quasi-experimental studies/RCT	Clinical response	Event rate	0.597	0.475-0.708	NR	0.118
				Overall	FMT				NR	NR	33	quasi-experimental studies/RCT	Clinical response	Event rate	0.546	0.461-0.628	NR	0.287
			IBD subtype	Any IBD	FMT				NR	NR	3	quasi-experimental studies/RCT	Clinical response	Event rate	0.581	0.150-0.915	NR	0.757
				Chronic pouchitis	FMT				NR	32	3	quasi-experimental studies/RCT	Clinical response	Event rate	0.218	0.037-0.668	NR	0.205
				Crohn's disease	FMT				NR	NR	7	quasi-experimental studies/RCT	Clinical response	Event rate	0.579	0.418-0.725	NR	0.336
				Ulcerative colitis	FMT				NR	NR	21	quasi-experimental studies/RCT	Clinical response	Event rate	0.546	0.440-0.648	NR	0.393
				Overall	FMT				NR	NR	34	quasi-experimental studies/RCT	Clinical response	Event rate	0.545	0.459-0.629	NR	0.301
71	2018	IBD	Overall		FMT	Control	56	57		113	2	RCTs	ESR at the longest follow up	MD	-0.85	-4.86-3.17	0	0.44
			Overall		FMT	Control	56	57		113	2	RCTs	CRP at the longest follow-up	MD	-3.35	-14.67-7.98	80.15	0.02
			Overall		FMT	Control	41	40		81	1	RCTs	Fecal calprotectin at the longest follow-up	MD	-156	-535.39-223.39	NA	NA
			Overall		FMT	Control	79	77		156	2	RCTs	Quality of life score: IBDQ	MD	16.00	0.09-31.91	59.75	0.11
			Clinical relapse at 12 weeks		FMT	Control	0/7	2/10		17	1	RCTs	Clinical relapse at 12 weeks	RR	0.28	0.02-4.98	NA	NA
			Serious adverse events		FMT	Control	10/140	7/137		277	4	RCTs	Serious adverse events	RR	1.40	0.55-3.58	0	0.99
			Deliver way	Overall	FMT	Control	10/140	7/137		277	4	RCTs	Serious adverse events	RR	1.40	0.55-3.58	0	0.99
				Upper gastrointestinal tract	FMT	Control	2/23	2/25		48	1	RCTs	Serious adverse events	RR	1.09	0.17-7.10	NA	NA
				Colonoscopy	FMT	Control	8/117	5/112		229	3	RCTs	Serious adverse events	RR	1.52	0.51-4.50	0	0.97
			Donor type	Overall	FMT	Control	10/140	7/137		277	4	RCTs	Serious adverse events	RR	1.40	0.55-3.58	0	0.99
				Single donor	FMT	Control	5/61	4/62		123	2	RCTs	Serious adverse events	RR	1.28	0.36-4.55	0	0.82
				Multidonor	FMT	Control	5/79	3/75		154	2	RCTs	Serious adverse events	RR	1.56	0.39-6.29	0	0.82
			Sensitivity analysis using fixed-effect model		FMT	Control	10/140	7/137		277	4	RCTs	Serious adverse events	RR	1.41	0.55-3.59	0	0.99
				Any adverse events	FMT	Control	50/64	49/65		129	2	RCTs	Any adverse events	RR	1.03	0.81-1.31	31.47	0.23
21	2019	UC	Overall		FMT					545	29	RCTs/non-RCTs	Primary cure rate	Event rate	0.3403	0.2133-0.4779	NR	NR
					FMT					545	29	RCTs/non-RCTs	Final cure rate	Event rate	0.3960	0.2539-0.5461	NR	NR
			Deliver way	Upper gastrointestinal tract	FMT					135	10	RCTs/non-RCTs	Primary cure rate	Event rate	0.4333	0.2184-0.6601	NR	NR
				Upper gastrointestinal tract	FMT					135	10	RCTs/non-RCTs	Final cure rate	Event rate	0.5049	0.2412-0.7673	NR	NR
				both upper and lower GI and/or unknown routes	FMT					334	14	RCTs/non-RCTs	Primary cure rate	Event rate	0.3708	0.1754-0.5870	NR	NR
				both upper and lower GI and/or unknown routes	FMT					334	14	RCTs/non-RCTs	Final cure rate	Event rate	0.3625	0.1832-0.5603	NR	NR
				both upper and lower GI and/or unknown routes	FMT					76	5	RCTs/non-RCTs	Primary cure rate	Event rate	0.1281	0.0029-0.3424	NR	NR
				both upper and lower GI and/or unknown routes	FMT					76	5	RCTs/non-RCTs	Final cure rate	Event rate	0.2952	0.0050-0.7231	NR	NR
36	2020	UC	Overall		FMT				86	258	23	uncontrolled cohort studies	clinical remission rate	Event rate	0.359	0.245-0.49.2	61.2	<0.001
			Overall		FMT				144	254	17	uncontrolled cohort studies	clinical response rate	Event rate	0.552	0.413-0.683	38.71	<0.001
			Overall		FMT				48	106	6	uncontrolled cohort studies	endoscopic remission rate	Event rate	0.422	0.230-0.641	67.66	0.009
			Overall		FMT	autologous FMT, sham FMT,	126	106		232	6	controlled cohort	Clinical and endoscopic improvements	OR	3.728	2.082-6.675	0	0.469

			water enemas, isotonic courses of therapy, sulfasalazine tablets, or antibiotics alone saline, standard						studies+RCTs						
Overall		FMT	autologous FMT, sham FMT, water enemas, isotonic courses of therapy, sulfasalazine tablets, or antibiotics alone saline, standard	207	269		476	6	controlled cohort studies+RCTs	Clinical remission rate	OR	3.011	1.566-5.790	0	0.981
Overall		FMT	autologous FMT, sham FMT, water enemas, isotonic courses of therapy, sulfasalazine tablets, or antibiotics alone saline, standard	333	375		708	12	controlled cohort studies+RCTs	Disease improvement	OR	3.392	2.196-5.240	0	0.981
Frequency	single donor	FMT	autologous FMT, sham FMT, water enemas, isotonic courses of therapy, sulfasalazine tablets, or antibiotics alone saline, standard			NR	NR	20	controlled or uncontrolled cohort studies + RCTs	eclinical improvement rate	Event rate	0.49	0.34-0.57	0.71	0.001
	rmultiple donors	FMT	autologous FMT, sham FMT, water enemas, isotonic courses of therapy, sulfasalazine tablets, or antibiotics alone saline, standard			NR	NR	4	controlled or uncontrolled cohort studies + RCTs	eclinical improvement rate	Event rate	0.42	0.23-0.64	0.69	0.02
	Overall	FMT	autologous FMT, sham FMT, water enemas, isotonic courses of therapy, sulfasalazine tablets, or antibiotics alone saline, standard			NR	NR	24	controlled or uncontrolled cohort studies + RCTs	eclinical improvement rate	Event rate	0.44	0.34-0.55	0.7	0.001
Bacterial fluid status	fresh samples	FMT	autologous FMT, sham FMT, water enemas, isotonic courses of therapy, sulfasalazine tablets, or antibiotics alone saline, standard			NR	NR	22	controlled or uncontrolled cohort studies + RCTs	clinical improvement rate	Event rate	0.34	0.25-0.46	NR	NR
	frozen samples	FMT	autologous FMT, sham FMT, water enemas, isotonic courses of therapy, sulfasalazine tablets, or antibiotics alone saline, standard			NR	NR	3	controlled or uncontrolled cohort studies + RCTs	clinical improvement rate	Event rate	0.47	0.36-0.58	0.01	0.36
	Overall	FMT	autologous FMT, sham FMT, water enemas, isotonic courses of therapy, sulfasalazine tablets, or antibiotics alone saline,			NR	NR	25	controlled or uncontrolled cohort studies + RCTs	clinical improvement rate	Event rate	0.41	0.33-0.49	0.61	0.001

						standard												
			total dosage of FMT	less than 275 g	FMT	autologous FMT, sham FMT, water enemas, isotonic courses of therapy, sulfasalazine tablets, or antibiotics alone saline, standard			NR	NR	17	controlled or uncontrolled cohort studies + RCTs	clinical improvement rate	Event rate	0.29	0.20-0.41	0.58	0.01
				more than 275 g	FMT	autologous FMT, sham FMT, water enemas, isotonic courses of therapy, sulfasalazine tablets, or antibiotics alone saline, standard			NR	NR	12	controlled or uncontrolled cohort studies + RCTs	clinical improvement rate	Event rate	0.52	0.38-0.64	0.6	0.004
				Overall	FMT	autologous FMT, sham FMT, water enemas, isotonic courses of therapy, sulfasalazine tablets, or antibiotics alone saline, standard			NR	NR	29	controlled or uncontrolled cohort studies + RCTs	clinical improvement rate	Event rate	0.39	0.31-0.48	0.63	0.001
			Deliver way	lower gastrointestinal tract for FMT	FMT	autologous FMT, sham FMT, water enemas, isotonic courses of therapy, sulfasalazine tablets, or antibiotics alone saline, standard			NR	NR	22	controlled or uncontrolled cohort studies + RCTs	clinical improvement rate	Event rate	0.44	0.35-0.54	0.69	0.001
				upper gastrointestinal tract	FMT	autologous FMT, sham FMT, water enemas, isotonic courses of therapy, sulfasalazine tablets, or antibiotics alone saline, standard			NR	NR	8	controlled or uncontrolled cohort studies + RCTs	clinical improvement rate	Event rate	0.32	0.13-0.72	0.56	0.026
				Overall	FMT	autologous FMT, sham FMT, water enemas, isotonic courses of therapy, sulfasalazine tablets, or antibiotics alone saline, standard			NR	NR	30	controlled or uncontrolled cohort studies + RCTs	clinical improvement rate	Event rate	0.42	0.33-0.51	0.67	0.001
			Pretreatment with antibiotics	yes	FMT	utologous FMT, sham FMT, water enemas,			NR	NR	9	controlled or uncontrolled cohort studies + RCTs	clinical improvement rate	Event rate	0.34	0.23-0.49	0.36	0.13
				no	FMT	utologous FMT, sham FMT, water enemas,			NR	NR	25	controlled or uncontrolled cohort studies + RCTs	clinical improvement rate	Event rate	0.43	0.32-0.56	0.74	0.001
				Overall	FMT	utologous FMT, sham FMT, water enemas,			NR	NR	34	controlled or uncontrolled cohort studies + RCTs	clinical improvement rate	Event rate	0.39	0.31-0.49	0.69	0.001
			Overall		FMT	utologous FMT, sham FMT, water enemas,			NR	NR	32	controlled or uncontrolled cohort studies + RCTs	clinical improvement rate	Event rate	0.06	0.04-0.085	0	0.99
40	2020	AUC	Overall		FMT	Control conditions	49/174	16/166		340	6	RCTs	Clinical remission	OR	3.634	1.940-6.808	0	<0.001
			Overall		FMT	Control conditions	93/174	53/166		340	6	RCTs	Clinical response	OR	2.634	1.441-4.815	33	0.002
			Overall		FMT	Control conditions	32/157	7/147		304	5	RCTs	Endoscopic remission, Mayo=0,1	OR	4.431	1.901-10.324	0	0.001
			Overall		FMT	Control conditions	13/64	13/65		129	2	RCTs	Endoscopic response	OR	1.065	0.432-2.625	0	0.892
41	2021	AUC	Delivery route	Total	FMT	Control	105/147	132/145		292	5	RCTs	combined clinical remission with endoscopic remission/response	RR	0.79	0.70-0.88	0	0.86
				Lower gastrointestinal tract	FMT	Control	85/117	104/112		229	3	RCTs	combined clinical remission with	RR	0.79	070-0.89	0	0.89

												endoscopic remission/response					
				Upper gastrointestinal tract	FMT	Control	20/30	28/33	63	2	RCTs	combined clinical remission with endoscopic remission/response	RR	0.79	0.58-1.09	8	0.30
			Number of donors	Total	FMT	Control	105/147	132/145	292	5	RCTs	combined clinical remission with endoscopic remission/response	RR	0.79	0.70-0.88	0	0.86
				Pooled donor	FMT	Control	60/86	77/83	169	3	RCTs	combined clinical remission with endoscopic remission/response	RR	0.76	0.65-0.89	0	0.69
				Single donor	FMT	Control	45/61	55/62	123	2	RCTs	combined clinical remission with endoscopic remission/response	RR	0.82	0.70-0.97	0	0.69
			Frequency of FMT administration	Total	FMT	Control	105/147	132/145	292	5	RCTs	combined clinical remission with endoscopic remission/response	RR	0.79	0.70-0.88	0	0.86
				Highter frequency	FMT	Control	63/86	80/85	171	3	RCTs	combined clinical remission with endoscopic remission/response	RR	0.79	0.69-0.90	0	0.66
				Lower frequency	FMT	Control	42/61	52/60	121	2	RCTs	combined clinical remission with endoscopic remission/response	RR	0.79	0.65-0.96	0	0.47
			Delivery route	Total	FMT	Control	87/147	113/145	292	5	RCTs	Clinical remission	RR	0.77	0.65-0.90	0	0.43
				Lower gastrointestinal tract	FMT	Control	66/117	89/112	229	3	RCTs	Clinical remission	RR	0.71	0.59-0.86	0	0.61
				Upper gastrointestinal tract	FMT	Control	21/30	24/33	63	2	RCTs	Clinical remission	RR	0.95	0.70-1.29	0	0.50
			Number of donors	Total	FMT	Control	87/147	113/145	292	5	RCTs	Clinical remission	RR	0.77	0.65-0.90	0	0.43
				Pooled donor	FMT	Control	48/86	68/83	169	3	RCTs	Clinical remission	RR	0.69	0.56-0.85	0	0.73
				Single donor	FMT	Control	39/61	45/62	123	2	RCTs	Clinical remission	RR	0.88	0.69-1.13	0	0.33
			Frequency of FMT administration	Total	FMT	Control	87/147	113/145	292	5	RCTs	Clinical remission	RR	0.77	0.65-0.90	0	0.43
				Highter frequency	FMT	Control	51/86	67/85	171	3	RCTs	Clinical remission	RR	0.76	0.62-0.93	0	0.81
				Lower frequency	FMT	Control	36/61	46/60	121	2	RCTs	Clinical remission	RR	0.80	0.50-1.28	70	0.07
			Delivery route	Total	FMT	Control	124/147	137/145	292	5	RCTs	Endoscopic remission	RR	0.91	0.84-0.99	6	0.37
				Lower gastrointestinal tract	FMT	Control	99/117	106/112	229	3	RCTs	Endoscopic remission	RR	0.90	0.83-0.98	0	0.55
				Upper gastrointestinal tract	FMT	Control	25/30	31/33	63	2	RCTs	Endoscopic remission	RR	0.82	0.47-1.45	69	0.07
			Number of donors	Total	FMT	Control	124/147	137/145	292	5	RCTs	Endoscopic remission	RR	0.91	0.84-0.99	6	0.37
				Pooled donor	FMT	Control	74/86	80/83	169	3	RCTs	Endoscopic remission	RR	0.91	0.82-1.01	13	0.32
				Single donor	FMT	Control	50/61	57/62	123	2	RCTs	Endoscopic remission	RR	0.91	0.76-1.10	49	0.16
			Frequency of FMT administration	Total	FMT	Control	124/147	137/145	292	5	RCTs	Endoscopic remission	RR	0.91	0.84-0.99	6	0.37
				Highter frequency	FMT	Control	69/86	79/85	171	3	RCTs	Endoscopic remission	RR	0.87	0.74-1.03	36	0.21
				Lower frequency	FMT	Control	55/61	58/60	121	2	RCTs	Endoscopic remission	RR	0.93	0.84-1.02	0	0.34
39	2017	IBD	Age	Paediatric UC	FMT				34	6	Cohorts	Clinical remission	Event rate	0.23	0.07-0.51	35	0.171
				Adults UC	FMT				34	6	Cohorts	Clinical remission	Event rate	0.34	0.24-0.46	58	0.001
				Paediatric CD	FMT				13	2	Cohorts	Clinical remission	Event rate	0.54	0.28-0.78	0	0.853
				Adults CD	FMT				13	2	Cohorts	Clinical remission	Event rate	0.46	0.18-0.77	71	0.017
			Study type	Cohorts UC	FMT				NR	24	Cohorts	Clinical remission	Event rate	0.325	0.234-0.432	54.135	0.001
				Cohorts CD	FMT				NR	6	Cohorts	Clinical remission	Event rate	0.518	0.311-0.719	52.230	0.063
				RCT UC	FMT				NR	4	RCTs	Clinical remission	OR	2.885	1.359-6.127	37.288	0.188
				RCT UC	FMT				NR	3	RCTs	Clinical remission	OR	4.052	2.082-7.885	0.000	0.783
				Cohorts UC	FMT				NR	20	Cohorts	Clinical response	Event rate	0.521	0.398-0.641	58.140	0.783
				Cohorts CD	FMT				NR	4	Cohorts	Clinical response	Event rate	0.632	0.297-0.875	71.093	0.016
				RCT UC	FMT				NR	4	RCTs	Clinical response	OR	2.481	1.182-5.209	51.654	0.102
				RCT UC	FMT				NR	3	RCTs	Clinical response	OR	3.389	1.904-6.035	0.000	0.442
			Age	Cohorts UC	Adult population				NR	18	Cohorts	Clinical remission	Event rate	0.344	0.243-0.462	58.421	0.001
				Cohorts UC	Paediatric population				NR	6	Cohorts	Clinical remission	Event rate	0.225	0.074-0.513	35.391	0.171
				Cohorts CD	Adult population				NR	4	Cohorts	Clinical remission	Event rate	0.455	0.177-0.765	70.671	0.017
				Cohorts CD	Paediatric population				NR	2	Cohorts	Clinical remission	Event rate	0.281	0.281-0.777	0.000	0.853
			Deliver way	Cohorts UC	Upper gastrointestinal tract				NR	4	Cohorts	Clinical remission	Event rate	0.174	0.084-0.324	0.000	0.604

				Cohorts UC	Lower gastrointestinal tract					NR	14	Cohorts	Clinical remission	Event rate	0.357	0.237-0.499	57.216	0.004
				Cohorts CD	Upper gastrointestinal tract					NR	3	Cohorts	Clinical remission	Event rate	0.533	0.195-0.844	70.329	0.034
			Bacterial fluid status	Cohorts UC	Fresh					NR	14	Cohorts	Clinical remission	Event rate	0.280	0.153-0.457	63.076	0.001
				Cohorts UC	Frozen					NR	4	Cohorts	Clinical remission	Event rate	0.360	0.134-0.671	62.680	0.045
				Cohorts CD	Fresh					NR	4	Cohorts	Clinical remission	Event rate	0.360	0.140-0.660	29.278	0.236
			Frequency	Cohorts UC	Number of infusions (1)					NR	11	Cohorts	Clinical remission	Event rate	0.234	0.108-0.434	69.297	0.000
				Cohorts UC	Number of infusions (2-4)					NR	3	Cohorts	Clinical remission	Event rate	0.276	0.123-0.509	0.000	0.508
				Cohorts UC	Number of infusions (5-10)					NR	4	Cohorts	Clinical remission	Event rate	0.352	0.173-0.584	48.206	0.122
				Cohorts UC	Number of infusions (<10)					NR	18	Cohorts	Clinical remission	Event rate	0.271	0.171-0.401	58.220	0.001
				Cohorts UC	Number of infusions (>10)					NR	3	Cohorts	Clinical remission	Event rate	0.487	0.208-0.774	28.751	0.246
				Cohorts CD	Number of infusions (1)					NR	5	Cohorts	Clinical remission	Event rate	0.588	0.408-0.748	36.692	0.177
				Cohorts CD	Number of infusions (<10)					NR	6	Cohorts	Clinical remission	Event rate	0.518	0.311-0.719	52.230	0.063
			Bowel lavage	Cohorts UC	Bowel lavage - Yes					NR	16	Cohorts	Clinical remission	Event rate	0.299	0.185-0.445	57.162	0.002
				Cohorts UC	Bowel lavage - No					NR	3	Cohorts	Clinical remission	Event rate	0.471	0.324-0.623	9.400	0.332
				Cohorts CD	Bowel lavage - Yes					NR	6	Cohorts	Clinical remission	Event rate	0.518	0.311-0.719	52.230	0.063
			Pretreatment with antibiotics	Cohorts UC	Pre-antibiotic-Yes					NR	7	Cohorts	Clinical remission	Event rate	0.329	0.170-0.540	58.246	0.026
				Cohorts UC	Pre-antibiotic-No					NR	13	Cohorts	Clinical remission	Event rate	0.278	0.156-0.444	60.781	0.002
				Cohorts CD	Pre-antibiotic-Yes					NR	3	Cohorts	Clinical remission	Event rate	0.476	0.246-0.717	0.000	0.420
				Cohorts CD	Pre-antibiotic-No					NR	3	Cohorts	Clinical remission	Event rate	0.535	0.224-0.822	73.806	0.022
			Donor type	Cohorts UC	Related donor					NR	2	Cohorts	Clinical remission	Event rate	0.245	0.000-0.995	90.299	0.001
				Cohorts UC	Unrelated donor					NR	8	Cohorts	Clinical remission	Event rate	0.362	0.201-0.561	56.449	0.024
				Cohorts CD	Unrelated donor					NR	2	Cohorts	Clinical remission	Event rate	0.398	0.103-0.792	40.661	0.194
			Stage	Cohorts UC	Moderate					NR	17	Cohorts	Clinical remission	Event rate	0.330	0.218-0.465	57.241	0.002
				Cohorts UC	Severe					NR	3	Cohorts	Clinical remission	Event rate	0.337	0.074-0.763	62.788	0.068
				Cohorts CD	Moderate					NR	4	Cohorts	Clinical remission	Event rate	0.629	0.478-0.758	18.291	0.299
38	2020	UC	Overall	Single-dose	FMT	Placebo	104/217	67/214		431	7	RCTs	Clinical remission rate	OR	2.29	1.48-3.53	30	0.20
			Deliver way	Lower gastrointestinal tract	FMT	Placebo	95/187	57/181		368	5	RCTs	Clinical remission rate	OR	2.70	1.67-4.37	32	0.21
				Upper gastrointestinal tract	FMT	Placebo	9/30	10/33		63	2	RCTs	Clinical remission rate	OR	0.98	0.33-2.89	0	0.85
				Total	FMT	Placebo	104/217	67/214		431	7	RCTs	Clinical remission rate	OR	2.29	1.48-3.53	30	0.20
			Donor type	Multi-donor	FMT	Placebo	53/140	24/137		277	4	RCTs	Clinical remission rate	OR	2.93	1.67-5.17	37	0.19
				Single-donors	FMT	Placebo	29/38	22/38		76	2	RCTs	Clinical remission rate	OR	2.64	0.87-8.01	0	0.44
				Total	FMT	Placebo	82/178	46/175		353	6	RCTs	Clinical remission rate	OR	2.87	1.73-4.75	7	0.37
			Bacterial fluid status	Frozen	FMT	Placebo	71/133	42/130		263	4	RCTs	Clinical remission rate	OR	2.76	1.59-4.79	29	0.24
				Fresh	FMT	Placebo	11/45	4/45		90	2	RCTs	Clinical remission rate	OR	3.47	0.99-12.23	11	0.29
				Total	FMT	Placebo	82/178	46/175		353	6	RCTs	Clinical remission rate	OR	2.87	1.73-4.75	7	0.37
			Overall		FMT	Placebo	26/210	23/206		416	6	RCTs	Serious adverse events	OR	1.37	0.63-2.96	0	0.85
21	2019	CD	CD		FMT				NR	115	10	RCTs/non-RCTs	Primary cure rate	Event rate	0.5227	0.3349-0.7080	NR	NR
					FMT				NR	115	10	RCTs/non-RCTs	Final cure rate	Event rate	0.4746	0.2939-0.6581	NR	NR
			Deliver way	Upper gastrointestinal tract	FMT				NR	67	6	RCTs/non-RCTs	Primary cure rate	Event rate	0.6629	0.5294-0.7868	NR	NR
				Upper gastrointestinal tract	FMT				NR	67	6	RCTs/non-RCTs	Final cure rate	Event rate	0.6206	0.3997-0.8224	NR	NR
				Both upper and lower GI and/or unknown routes	FMT				NR	48	4	RCTs/non-RCTs	Primary cure rate	Event rate	0.2706	0.0547-0.5474	NR	NR
				Both upper and lower GI and/or unknown routes	FMT				NR	48	4	RCTs/non-RCTs	Final cure rate	Event rate	0.2706	0.0547-0.5474	NR	NR
37	2021	CD	Overall		FMT				109	217	7	Cohorts	Clinical remission	Event rate	0.506	0.354-0.656	56.718	0.021
					FMT				145	202	5	Cohorts	Clinical response	Event rate	0.717	0.572-0.827	33.723	0.197
			Age	Adults	FMT				40	73	4	Cohorts	Clinical response	Event rate	0.384	0.152-0.684	70.448	0.017
				Children	FMT				9	16	2	Cohorts	Clinical response	Event rate	0.545	0.125-0.909	71.458	0.061
			Deliver way	Upper administration	FMT				NR	NR	2	Cohorts	Clinical response	Event rate	0.387	0.015-0.963	80.812	0.022
				Lower GI endoscopy	FMT				NR	NR	3	Cohorts	Clinical response	Event rate	0.322	0.116-0.632	55.529	0.106

			Bacterial fluid status	Fresh	FMT				NR	NR	4	Cohorts	Clinical response	Event rate	0.5	0.218-0.782	75.061	0.045
				Frozen	FMT				NR	NR	2	Cohorts	Clinical response	Event rate	0.299	0.044-0.798	63.416	0.042
			Pre-antibiotic-Yes	Yes	FMT				NR	NR	3	Cohorts	Clinical response	Event rate	0.368	0.076-0.805	73.422	0.023
				No	FMT				NR	NR	4	Cohorts	Clinical response	Event rate	0.564	0.437-0.683	39.107	0.177
			Country	America	FMT				NR	NR	4	Cohorts	Clinical response	Event rate	0.429	0.184-0.715	62.296	0.047
				China	FMT				NR	NR	2	Cohorts	Clinical response	Event rate	0.584	0.507-0.656	0	0.345
42	2020	CD	Overall		FMT				NR	256	7	SATs	Clinical remission rate	OR	2.57	1.95-3.40	0	0.47
			Overall		FMT				NR	256	7	SATs	Clinical response rate	OR	1.50	1.03-2.18	0.33	0.18
			Age	adult	FMT				NR	240	5	SATs	Clinical response rate	OR	2.60	1.77-3.83	0.27	0.24
				children	FMT				NR	16	2	SATs	Clinical response rate	OR	2.98	0.96-9.27	0.08	0.77
				adult	FMT				NR	240	5	SATs	Clinical remission rate	OR	1.51	1.07-2.13	0.26	0.25
				children	FMT				NR	16	2	SATs	Clinical remission rate	OR	1.20	1.14-10.04	0.71	0.06
			Bacterial fluid status	Frozen stool	FMT				NR	188	3	SATs	Clinical response rate	OR	2.64	1.35-5.19	0.69	0.09
				Fresh stool	FMT				NR	68	4	SATs	Clinical response rate	OR	2.75	1.60-4.71	0	0.86
				Frozen stool	FMT				NR	188	3	SATs	Clinical remission rate	OR	1.60	0.71-2.75	0.36	0.19
				Fresh stool	FMT				NR	68	4	SATs	Clinical remission rate	OR	1.39	0.71-2.75	0.38	0.19
**CDI patients with IBD**																		
44	2018	CDI patients with IBD	Overall		FMT				284	346	9	Cohorts	Initial cure rate	Event rate	0.81	0.76-0.85	0	0.47
			Overall		FMT				144	160	4	Cohorts	Overall cure rate	Event rate	0.89	0.83-0.93	2	0.38
			Overall		FMT				37	213	6	Cohorts	Recurrence rate	Event rate	0.19	0.13-0.27	33	0.19
				IBD patients	FMT	Without IBD patients	149/188	678/774		962	6	Cohorts	Cure rate	RR	0.92	0.81-1.05	53	0.06
			IBD subtype	Crohn's disease	FMT				97	124	5	Cohorts	Initial cure rate	Event rate	0.78	0.70-0.84	0	0.79
				Ulcerative colitis	FMT				96	109	5	Cohorts	Initial cure rate	Event rate	0.85	0.77-0.91	0	0.43
				Overall	FMT				193	233	10	Cohorts	Initial cure rate	Event rate	0.8	0.74-0.85	0	0.54
43	2021	CDI patients with IBD	Age	adult, single FMT	FMT				363	457	11	Cohorts	Clinical CDI Cure Rate	Event rate	0.78	0.73-0.83	39	NR
				pediatric, single FMT	FMT				106	141	6	Cohorts	Clinical CDI Cure Rate	Event rate	0.78	0.58-0.93	59	NR
				adult, FMT	FMT				141	527	13	Cohorts	IBD Flare After FMT	Event rate	0.268	0.225-0.315	9	NR
				pediatric, FMT	FMT				13	120	3	Cohorts	IBD Flare After FMT	Event rate	0.108	0.057-0.185	43	NR
				adult, FMT	FMT				78	231	7	Cohorts	IBD Symptom Improvement	Event rate	0.338	0.267-0.421	64	NR
				pediatric, FMT	FMT				10	31	3	Cohorts	IBD Symptom Improvement	Event rate	0.322	0.154-0.593	37	NR
				adult, FMT	FMT				27	371	12	Cohorts	Rate of Colectomy After FMT	Event rate	0.073	0.047-0.105	56	NR
				pediatric, FMT	FMT				3	29	4	Cohorts	Rate of Colectomy After FMT	Event rate	0.103	0.021-0.302	23	NR
**IBS**																		
47	2019	IBS	Method of administration	Oral capsule	FMT	Placebo capsules	32/50	16/50		100	2	RCTs	failing to respond to therapy	RR	1.96	1.19-3.20	14	0.28
				Colonoscopy	FMT	Autologous stool	26/66	24/37		103	2	RCTs	failing to respond to therapy	RR	0.63	0.43-0.93	0	0.71
				Nasojejunal tube	FMT	Autologous stool	21/42	16/22		64	1	RCTs	failing to respond to therapy	RR	0.69	0.46-1.02	NA	NA
				Overall	FMT	Placebo	79/158	56/109		267	5	RCTs	failing to respond to therapy	RR	0.98	0.58-1.66	78	0.001
			Deliver way	Upper gastrointestinal tract	FMT	Placebo	NR	NR		164	3	RCTs	No benefit	RR	1.35	0.58-3.14	85	0.001
				Lower gastrointestinal tract	FMT	Placebo	NR	NR		103	2	RCTs	No benefit	RR	0.63	0.43-0.93	0	0.71
			Type of placebo used	Inactive placebo	FMT	Placebo	NR	NR		100	2	RCTs	failing to respond to therapy	RR	1.96	1.19-3.20	14	0.28
				Autologous stool	FMT	Placebo	NR	NR		167	3	RCTs	failing to respond to therapy	RR	0.66	0.50-0.87	0	0.89
46	2019	IBS	Overall		FMT	Patients' own feces or placebo	75/152	52/102		254	4	RCTs	clinical response rate	RR	0.93	0.48-1.79	0.79	0.01
			Frequency	Single-dose	FMT	Patients' own feces or placebo	57/102	18/52		154	2	RCTs	clinical response rate	RR	1.59	1.06-2.39	0	0.66
				Multiple-dose	FMT	Patients' own feces or placebo	18/50	34/50		100	2	RCTs	clinical response rate	RR	0.54	0.34-0.85	0.13	0.28
45	2022	IBS	Follow-up time		FMT	Placebo	93/130	26/81		211	2	RCTs	IBS improvement symptoms 4 weeks	RR	1.33	0.22-7.89	92	0.0003

					FMT	Placebo	160/272	75/176		448	6	RCTs	IBS improvement symptoms 12 weeks	RR	1.19	0.67-2.13	84	<0.0001
			Follow-up time		FMT	Placebo	227	143		370	5	RCTs	IBS-SSS 4 weeks	MD	-20	−71.3-30.63	82	0.0001
					FMT	Placebo	217	142		359	5	RCTs	IBS-SSS 12 weeks	MD	−30.79	−99.45-37.96	90	<0.0001
					FMT	Placebo	112	93		205	4	RCTs	IBS-SSS 24 weeks	MD	6.49	−74.81-87.79	90	<0.0001
			Follow-up time		FMT	Placebo	131	78		209	2	RCTs	IBS-QOL 4 weeks	MD	7.47	2.05-12.89	0	0.86
					FMT	Placebo	195	122		317	4	RCTs	IBS-QOL 12 weeks	MD	9.99	5.78-14.19	77	0.005
					FMT	Placebo	35	42		77	2	RCTs	IBS-QOL 24 weeks	MD	8.49	0.47–16.52	4	0.31
					FMT				198	341	12	SATs	IBS improvement symptoms	Event rate	0.578	0.456-0.699	80.94	<0.001
					FMT				NR	NR	10	SATs	IBS-SSS	MD	−74	−101.7-(−46.3)	83.75	<0.001
88	2022	IBS	Total		FMT	Placebo	105/290	111/186		476	7	RCTs	global symptom not improving at 12 weeks	RR	0.75	0.43-1.31	87	<0.00001
			Route of delivery	oral capsules	FMT	Placebo	32/51	16/49		100	2	RCTs	global symptom not improving at 12 weeks	RR	1.88	1.06-3.35	36	0.21
				gastroscope	FMT	Placebo	38/153	56/74		227	2	RCTs	global symptom not improving at 12 weeks	RR	0.37	0.14-0.99	91	0.001
				Colonoscopy	FMT	Placebo	35/86	39/63		149	3	RCTs	global symptom not improving at 12 weeks	RR	0.70	0.51-0.96	0	0.44
			Mixed or single donor sample	Mixed	FMT	Placebo			NR	NR	2	RCTs	global symptom not improving at 12 weeks	RR	1.22	0.30-5.04	91	NR
				Single	FMT	Placebo			NR	NR	5	RCTs	global symptom not improving at 12 weeks	RR	0.62	0.33-1.17	86	NR
			Bacterial fluid status	Frozen stool	FMT	Placebo			NR	NR	4	RCTs	global symptom not improving at 12 weeks	RR	0.91	0.31–2.68	94	NR
				Fresh stool	FMT	Placebo			NR	NR	2	RCTs	global symptom not improving at 12 weeks	RR	0.59	0.41–0.85	0	NR
				Both	FMT	Placebo			NR	NR	1	RCTs	global symptom not improving at 12 weeks	RR	0.60	0.37–0.98	NA	NA
			Placebo type	Inactive solution	FMT	Placebo			NR	NR	2	RCTs	global symptom not improving at 12 weeks	RR	1.88	1.06–3.35	36	NR
				Autologous stool	FMT	Placebo			NR	NR	5	RCTs	global symptom not improving at 12 weeks	RR	0.52	0.32–0.86	79	NR
			Center site	Single center	FMT	Placebo			NR	NR	4	RCTs	global symptom not improving at 12 weeks	RR	0.46	0.27–0.77	77	NR
				Two centers	FMT	Placebo			NR	NR	1	RCTs	global symptom not improving at 12 weeks	RR	2.57	1.30–5.09	NA	NA
				Three centers	FMT	Placebo			NR	NR	2	RCTs	global symptom not improving at 12 weeks	RR	1.10	0.70–1.72	21	NR
			Study setting	Primary care	FMT	Placebo			NR	NR	1	RCTs	global symptom not improving at 12 weeks	RR	0.60	0.37–0.98	NA	NA
				Tertiary care	FMT	Placebo			NR	NR	2	RCTs	global symptom not improving at 12 weeks	RR	1.10	0.70–1.72	21	NR
				Primary and tertiary care	FMT	Placebo			NR	NR	4	RCTs	global symptom not improving at 12 weeks	RR	0.65	0.25–1.66	92	NR
			IBS criteria	Rome III	FMT	Placebo			NR	NR	6	RCTs	global symptom not improving at 12 weeks	RR	0.91	0.59–1.42	73	NR
				Rome IV	FMT	Placebo			NR	NR	1	RCTs	global symptom not improving at 12 weeks	RR	0.23	0.15–0.35	NA	NA
			IBS subtype	Non-constipation subtype	FMT	Placebo			NR	NR	3	RCTs	global symptom not improving at 12 weeks	RR	0.77	0.47–1.28	66	NR
				All subtype	FMT	Placebo			NR	NR	4	RCTs	global symptom not improving at 12 weeks	RR	0.73	0.26–2.04	92	NR
			IBS severity	All	FMT	Placebo			NR	NR	2	RCTs	global symptom not improving at 12 weeks	RR	0.77	0.49–1.20	8	NR
				Moderate to severe	FMT	Placebo			NR	NR	4	RCTs	global symptom not improving at 12 weeks	RR	0.82	0.29–2.33	93	NR
				Refractory	FMT	Placebo			NR	NR	1	RCTs	global symptom not improving at 12 weeks	RR	0.60	0.39–0.92	NA	NA
			FMT dosage	≤30 g	FMT	Placebo			NR	NR	2	RCTs	global symptom not improving at 12 weeks	RR	0.77	0.49–1.20	8	NR
				>30 g	FMT	Placebo			NR	NR	4	RCTs	global symptom not improving at 12 weeks	RR	0.82	0.29–2.33	93	NR
				Not specified	FMT	Placebo			NR	NR	1	RCTs	global symptom not improving at 12 weeks	RR	0.60	0.39–0.92	NA	NA
			IBS-QOL 12 weeks		FMT	Placebo	206	131		337	5	RCTs	IBS-QOL 12 weeks	MD	9.39	3.86-14.91	38	0.17
			Overall		FMT	Placebo	126/222	47/144		366	5	RCTs	Adverse events	RR	1.20	0.59-2.47	83	<0.0001
			Global symptom not improving at 1 year		FMT	Placebo	80/121	55/73		194	3	RCTs	Global symptom not improving at 1 year	RR	0.90	0.72-1.12	48	0.15
**Functional gut disorders**																		
40	2020	Functional gut disorders	Overall		FMT	treatment as usual (TAU)	149/219	59/138			5	RCTs	Clinical response	OR	1.699	0.273-10.588	92	0.739
					FMT	treatment as usual (TAU)	NR	NR			3	RCTs	IBS-SSS	Hedge's g	0.282	-1.373-1.937	97	0.739
**Psychiatric outcomes**																		
40	2020	Psychiatric outcomes	Overall	30g FMT 3 month	FMT	Baseline	54	55		109	3	RCTs	Fatigue Assessment Scale (FAS) at 3 mouth	MD	-1.9	-2.7-(-1.1)	NA	NA
				60g FMT 3 month	FMT	Baseline	55	55		110	3	RCTs	FAS)at 3 mouth	MD	-2.3	-3.1-(-1.5)	NA	NA
**AMR**																		
51	2021	AMR	Overall		FMT				57	102	9	RCTs/Cohorts/case-control	Decolonization success at 1 month	Event rate	0.58	0.42-0.74	NR	NR

											studies						
				FMT	Treatment Naive	21/57	42/54		101	4	RCTs/Cohorts/case-control studies	AMR remission at 1 mouth	Event rate	0.44	0.20-0.99	65	0.03
				FMT	Treatment Naive	8/26	26/30		56	2	RCTs	AMR remission at 1 mouth	Event rate	0.37	0.18-0.79	23	0.25
52	2019	AMR	Overall	FMT		50		24	50	3	case series	Decolonization success at 1 month	Event rate	0.45	0.20-0.74		
**Metabolic syndrome and obesity**																	
53	2020	Metabolic syndrome	Follow-up time	FMT	Placebo	80	67		147	6	RCTs	Early (2-6 weeks) HbA1c	MD	-1.69	-2.81-(-0.56)	46	0.1
				FMT	Placebo	80	66		146	6	RCTs	Early HDL cholesterol	MD	0.09	0.02-0.15	5	0.39
				FMT	Placebo	80	66		146	6	RCTs	Early LDL cholesterol	MD	0.19	0.05-0.34	0	0.6
				FMT	Placebo	78	66		144	6	RCTs	Early Fasting glucose	MD	-0.09	-0.22- (0.04)	19	0.29
				FMT	Placebo	66	54		120	6	RCTs	Early triglycerides	MD	-0.02	-0.52-(0.49)	91	0.000001
				FMT	Placebo	69	55		124	5	RCTs	Early total cholesterol	MD	0.00	-0.17-(0.16)	0	0.44
				FMT	Placebo	59	46		105	4	RCTs	Early HOMA-IR	MD	-0.35	-1.56-(0.86)	69	1
				FMT	Placebo	22	21		43	2	RCTs	Late (12 weeks) HbA1c	MD	-0.05	-2.17-(2.06)	0	0.91
				FMT	Placebo	22	21		43	2	RCTs	Late LDL cholesterol	MD	-0.32	-0.92-(0.29)	0	0.91
				FMT	Placebo	22	21		43	2	RCTs	Late HDL cholesterol	MD	0.22	-0.04-(0.48)	0	0.76
				FMT	Placebo	22	21		43	2	RCTs	Late triglycerides	MD	0.09	-0.24-(0.42}	0	0.71
53	2020	Obesity	Follow-up time	FMT	Placebo	46	32		78	3	RCTs	Early BMI	MD	-0.85	-2.90-(1.20)	86	0.0001
				FMT	Placebo	56	44		100	4	RCTs	Early Weight	MD	1.67	-9.50-(12.84)	86	0.0001
				FMT	Placebo	23	15		38	2	RCTs	Late BMI	MD	-0.34	-1.81-(1.13)	0.71	0.07
				FMT	Placebo	23	15		38	2	RCTs	Late hip width	MD	-0.83	-4.68-(3.03)	0	0.56
				FMT	Placebo	35	30		65	3	RCTs	Late weight	MD	0.32	-6.81-(7.45)	0	0.95
**Functional constipation**																	
49	2021	Functional constipation	Overall	FMT+laxatives	laxatives	74/82	54/81		163	2	RCTs	total effective rate	RR	1.35	1.14-1.60	13	0.28
				FMT+laxatives	laxatives	103	103		206	3	RCTs	Bristol stool form scale score	WMD	1.04	0.57-1.51	76	0.02
				FMT+laxatives	laxatives	73	73		146	2	RCTs	Wexner constipation scale	WMD	−3.25	−5.58-(−0.92)	92	0.0003
				FMT+laxatives	laxatives	80	80		160	2	RCTs	Knowles-eccersley-scott-symptom score	WMD	−5.65	−7.62-(−3.69)	0	0.38
				FMT+laxatives	laxatives	123	123		246	3	RCTs	Patient assessment of constipation quality of life score	WMD	−18.56	−26.43-(−10.68)	78	0.01
**GVHD**																	
50	2021	GVHD	Overall	FMT				NR	87	11	Case series/case reports	complete remission	Event rate	0.437	NR	NR	NR
			Overall	FMT				NR	87	11	Case series/case reports	partial remission	Event rate	0.207	NR	NR	NR
			Overall	FMT				NR	87	11	Case series/case reports	overall response rate	Event rate	0.644	NR	NR	NR

Note: 95% CI, 95% confidence interval; RCDI, recurrent *Clostridioides difficile* infection; IBD, inflammatory bowel disease; UC, Ulcerative colitis; AUC, active UC; CD, Crohn's disease; AMR, antimicrobial resistance; IBS, irritable bowel syndrome; CDAD, *Clostridioides difficile*-associated diarrhea; GVHD, graft versus host disease; RCTs, randomised clinical trials; SATs, single arm trials; NR, not reported; NA, not applicable; MT, medical therapy; SUCRA, the surface under the cumulative ranking

86. Tang, G., Yin, W. & Liu, W. Is frozen fecal microbiota transplantation as effective as fresh fecal microbiota transplantation in patients with recurrent or refractory Clostridium difficile infection: A meta-analysis? *Diagnostic microbiology and infectious disease* **88**, 322-329, doi:10.1016/j.diagmicrobio.2017.05.007 (2017).
87. Du, C., Luo, Y., Walsh, S. & Grinspan, A. Oral Fecal Microbiota Transplant Capsules Are Safe and Effective for Recurrent Clostridioides difficile Infection: A Systematic Review and Meta-Analysis. *Journal of clinical gastroenterology* **55**, 300-308, doi:10.1097/mcg.0000000000001495 (2021).
88. Wu, J., Lv, L. & Wang, C. Efficacy of Fecal Microbiota Transplantation in Irritable Bowel Syndrome: A Meta-Analysis of RCTs. *Front Cell Infect Microbiol* **12**, 827395, doi:10.3389/fcimb.2022.827395 (2022).

Appendix 6. Clinical practice guidelines and consensus on the fecal microbiota transplantation

[1]. Cammarota G, Ianiro G, Kelly CR, et al. International consensus conference on stool banking for faecal microbiota transplantation in clinical practice. Gut. 2019;68(12):2111-2121. doi:10.1136/gutjnl-2019-319548.

[2]. van Prehn J, Reigadas E, Vogelzang EH, et al. European Society of Clinical Microbiology and Infectious Diseases: 2021 update on the treatment guidance document for Clostridioides difficile infection in adults. Clin Microbiol Infect. 2021;27 Suppl 2:S1-S21. doi:10.1016/j.cmi.2021.09.038.

[3]. Mullish BH, Quraishi MN, Segal JP, et al. The use of faecal microbiota transplant as treatment for recurrent or refractory Clostridium difficile infection and other potential indications: joint British Society of Gastroenterology (BSG) and Healthcare Infection Society (HIS) guidelines. Gut. 2018;67(11):1920-1941. doi:10.1136/gutjnl-2018-316818.

[4]. Clostridioides difficile infection: antimicrobial prescribing. London: National Institute for Health and Care Excellence (NICE); July 23, 2021.

[5]. McDonald LC, Gerding DN, Johnson S, et al. Clinical Practice Guidelines for Clostridium difficile Infection in Adults and Children: 2017 Update by the Infectious Diseases Society of America (IDSA) and Society for Healthcare Epidemiology of America (SHEA). Clin Infect Dis. 2018;66(7):987-994. doi:10.1093/cid/ciy149.

[6]. Aira A, Arajol C, Casals-Pascual C, et al. Recommendations for stool donor selection for fecal microbiota transplant. Consensus document endorsed by the Catalan Society of Digestology, Catalan Society of Infectious diseases and Clinical Microbiology and the GEMBIOTA group from Spanish Society of Infectious Diseases and Clinical Microbiology. Enferm Infecc Microbiol Clin (Engl Ed). 2022;40(3):142-146. doi:10.1016/j.eimce.2021.12.001.

[7]. Keller JJ, Ooijevaar RE, Hvas CL, et al. A standardised model for stool banking for faecal microbiota transplantation: a consensus report from a multidisciplinary UEG working group. United European Gastroenterol J. 2021;9(2):229-247. doi:10.1177/2050640620967898.

[8]. Parenteral and Enteral Nutrition Branch of Chinese Medical Association; Enhanced Recovery After Surgery Branch of China International Health Care Promotion and Exchange Association; China Microecological Treatment Innovation Alliance; Microecology Committee of Shanghai Preventive Medicine Association. Zhonghua Wei Chang Wai Ke Za Zhi. 2020;23(Z1):5-13. doi:10.3760/cma.j.cn.441530-20200420-00231.

[9]. Parenteral and Enteral Nutrition Branch of Chinese Medical Association; Enhanced Recovery After Surgery Branch of China International Health Care Promotion and Exchange Association; China Microecological Treatment Innovation Alliance; Microecology Committee of Shanghai Preventive Medicine Association. Zhonghua Wei Chang Wai Ke Za Zhi. 2020;23(Z1):14-20. doi:10.3760/cma.j.cn.441530-20200420-00228.

[10]. Fecal Microbiota Transplantation-standardization Study Group. Nanjing consensus on methodology of washed microbiota transplantation. Chin Med J 2020;133:2330–2332. doi: 10.1097/CM9.0000000000000954.

[11]. Baunwall SMD, Dahlerup JF, Engberg JH, et al. Danish national guideline for the treatment of Clostridioides difficile infection and use of faecal microbiota transplantation (FMT). Scand J Gastroenterol. 2021;56(9):1056-1077. doi:10.1080/00365521.2021.1922749.

[12]. Piekarska A, Panasiuk A, Stępień PM. Clinical practice guidelines for Clostridioides (Clostridium) difficile infection and fecal microbiota transplant protocol - recommendations of the Polish Society od Epidemiology and Infectious Diseases. Przegl Epidemiol. 2020;74(1):69-87. doi:10.32394/pe.74.06.
[13]. Tacconelli E, Mazzaferri F, de Smet AM, et al. ESCMID-EUCIC clinical guidelines on decolonization of multidrug-resistant Gram-negative bacteria carriers. Clin Microbiol Infect. 2019;25(7):807-817. doi:10.1016/j.cmi.2019.01.005.
[14]. Ianiro G, Mullish BH, Kelly CR, et al. Reorganisation of faecal microbiota transplant services during the COVID-19 pandemic. Gut. 2020;69(9):1555-1563. doi:10.1136/gutjnl-2020-321829.
